# Rapid Screening of Diverse Biotransformations for Enzyme Evolution

**DOI:** 10.1021/jacsau.1c00027

**Published:** 2021-04-08

**Authors:** Emily E. Kempa, James L. Galman, Fabio Parmeggiani, James R. Marshall, Julien Malassis, Clement Q. Fontenelle, Jean-Baptiste Vendeville, Bruno Linclau, Simon J. Charnock, Sabine L. Flitsch, Nicholas J. Turner, Perdita E. Barran

**Affiliations:** † School of Chemistry, University of Manchester, Manchester Institute of Biotechnology, 131 Princess Street, Manchester M1 7DN, United Kingdom; ‡ Department of Chemistry, Materials and Chemical Engineering “G. Natta”, Politecnico di Milano, Via Mancinelli 7, 20131 Milano, Italy; § School of Chemistry, University of Southampton, Highfield, SO17 1BJ Southampton, United Kingdom; ∥ Prozomix Ltd., Building 4, West End Ind. Estate, Haltwhistle, Northumberland NE49 9HA, United Kingdom

**Keywords:** mass spectrometry, desorption electrospray ionization, high-throughput screening, biotransformation, enzyme evolution, biocatalysis

## Abstract

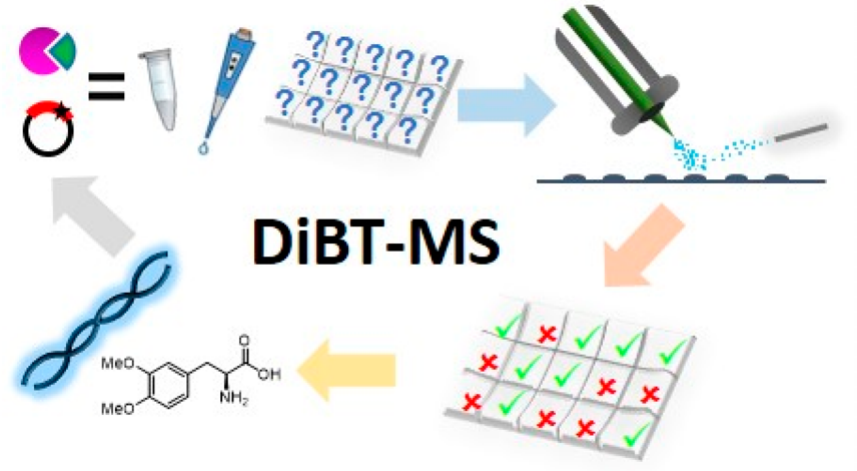

The lack of label-free high-throughput screening technologies presents a major bottleneck in the identification of active and selective biocatalysts, with the number of variants often exceeding the capacity of traditional analytical platforms to assess their activity in a practical time scale. Here, we show the application of direct infusion of biotransformations to the mass spectrometer (DiBT-MS) screening to a variety of enzymes, in different formats, achieving sample throughputs equivalent to ∼40 s per sample. The heat map output allows rapid selection of active enzymes within 96-well plates facilitating identification of industrially relevant biocatalysts. This DiBT-MS screening workflow has been applied to the directed evolution of a phenylalanine ammonia lyase (PAL) as a case study, enhancing its activity toward electron-rich cinnamic acid derivatives which are relevant to lignocellulosic biomass degradation. Additional benefits of the screening platform include the discovery of biocatalysts (kinases, imine reductases) with novel activities and the incorporation of ion mobility technology for the identification of product hits with increased confidence.

## Introduction

Biocatalysis provides an alternative and increasingly attractive sustainable pathway for the production of high-value chemical building blocks and intermediates. However, the application of biocatalysts in chemical synthesis is limited when using naturally occurring enzymes because of narrow substrate tolerance, low activity, and poor operational stability. During the past 10 years, advances in protein engineering and directed evolution have demonstrated that many of these important parameters can be altered to position many more biocatalysts for preparative organic synthesis. Despite the increasing use of directed evolution for improving biocatalyst performance, a major bottleneck remains in the laborious processes required for screening libraries of variant enzymes to identify candidates with improved properties.^1^ The majority of *in vitro* techniques that are available for analyzing enzyme libraries deploy chromatographic methods which provide high-quality data regarding chemical identity but are not suitable for libraries in excess of 10^4^ enzyme variants.

**Figure 1:**
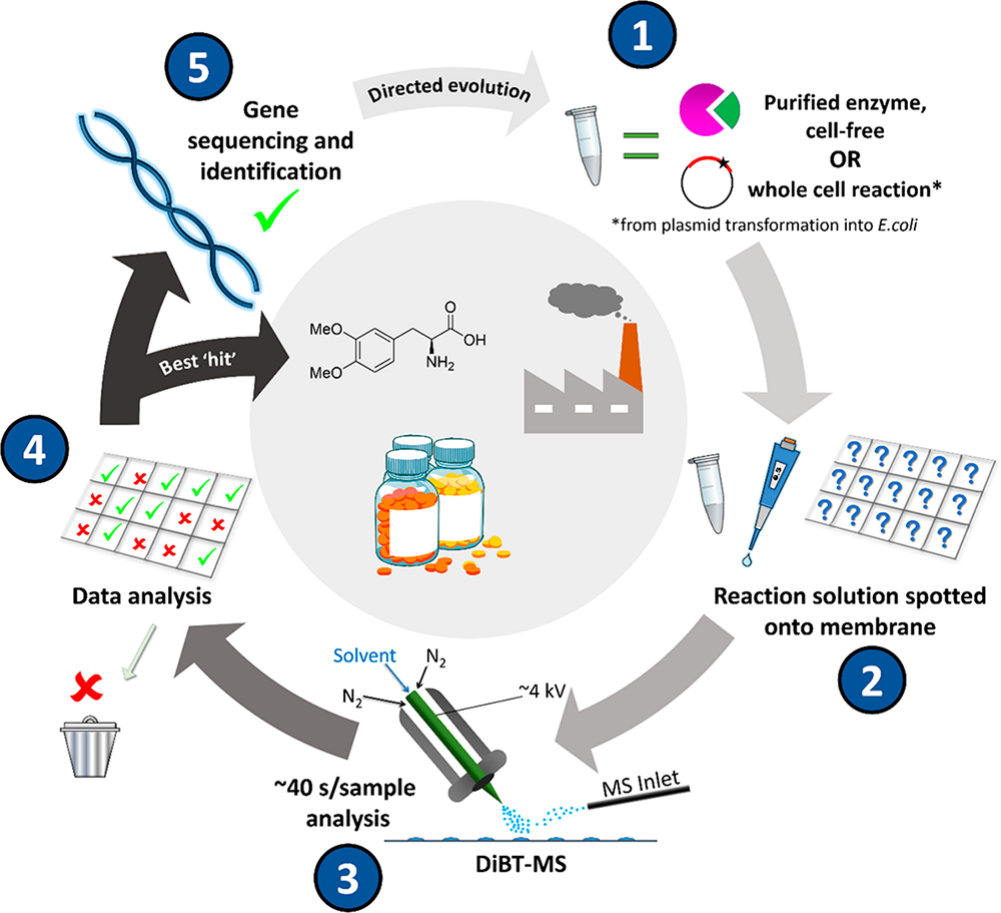
Overview of the DiBT-MS workflow for screening biocatalytic reactions and identifying improved enzyme variants. Initially, the reactions (whole cell or purified enzyme) are performed within Eppendorf tubes or 96/384-well plates (1) before spotting (0.5 μL) of the quenched reaction onto a nylon membrane grid (see photograph, Figure S1) prepared for DiBT-MS analysis (2). The membrane containing dried spotted reactions is subjected to DiBT-MS analysis where necessary with ion mobility (IM) separation (3), and the resulting mass- and mobility-resolved heat maps were assessed for wells with the highest product detection (4). The sample remaining in the corresponding 96/384-well plate or Eppendorf tube can then be extracted for DNA sequencing to identify the mutations responsible for improved activity (5).

Applying desorption electrospray ionization (DESI) MS to crude reaction mixtures permits the analysis of products *in situ*(2) (Figure 1), which has significant benefits for monitoring biocatalytic reactions compared to more traditional chromatographic MS methods. This approach shortens the analysis time and removes the need for much of the solvent. Further, the ability to correlate the spatial positions of spotted reactions and colonies with products provides a visual reference on the identified enzyme “hits”, followed by DNA extraction/PCR amplification to determine the sequence.^3^ Coupling the direct infusion of biotransformations to the mass spectrometer (DiBT-MS) high throughput screening method, which provides semiquantitative chemical information, to a protein engineering strategy accelerates the evolution of enzymes as demonstrated here.

## Results

### Kinase Screening (Purified Enzymes)

Selective phosphorylation of monosaccharides, mediated by sugar kinases, is an important reaction to access natural and modified glycans. Anomeric sugar kinases (galactokinases, GalKs,^4^ and *N*-acetylhexosamine kinases, NahKs^5^) are efficient enzymes for the conversion of their natural substrates but tolerate only limited modifications to the structure of the sugar although homologues can often display a considerably different substrate spectrum.^6,7^ These kinases therefore present attractive targets for rapid screening of various substrate/enzyme combinations to identify successful phosphorylation reactions.

**Figure 2:**
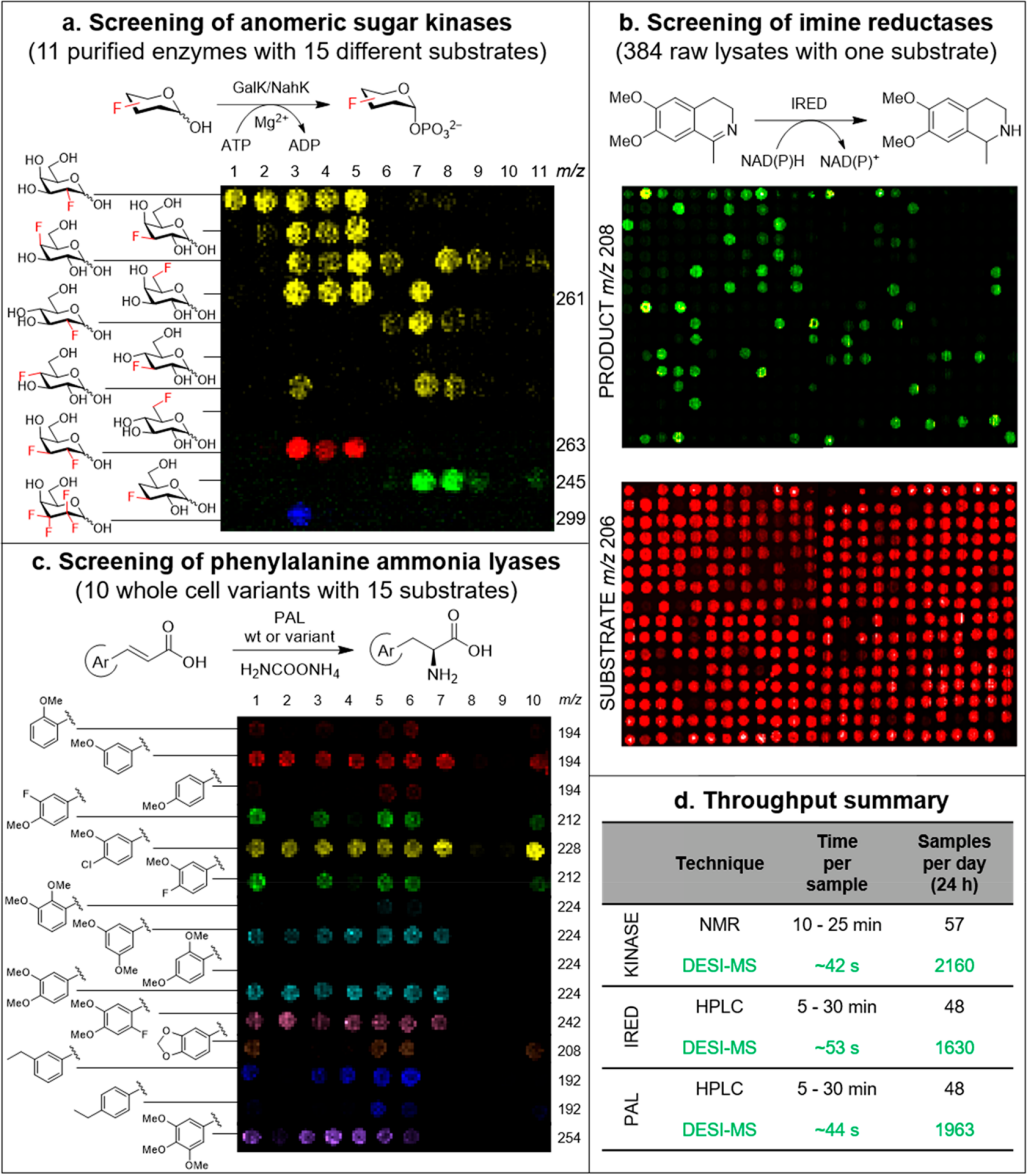
Representative results of DiBT-MS screening of diverse biotransformations. (a) DiBT-MS heat map obtained upon screening 11 purified kinase enzymes with 11 monosaccharide substrates. In total 15 substrates were screened, with the full results including reaction conversions as determined by ^19^F-NMR given in Figures S3 and S4. (b) Resulting heat maps for imine reductase (IRED) reactions performed in a 384-well metagenomic plate. The heat maps indicate areas in which starting material (red) and product (green) are present on the membrane with each *m*/*z* value detected simultaneously. (c) DiBT-MS heat map results for PAL whole cell reaction screening. 10 enzyme variants were screened against 15 cinnamic acid substrates for conversion to the corresponding phenylalanine derivative. Reaction conversions obtained by HPLC-(UV) analysis for each of these reactions are given in Figure S12. (d) Table to show the increase in throughput achieved for three specific reaction types when employing DiBT-MS as the primary screening technique in comparison to alternative techniques, wherein the longer time denotes discovery, and the shorter time indicates an optimized assay.

A panel of 11 different wild-type kinases (5 GalKs and 6 NahKs) were tested against 15 variously substituted deoxyfluorinated monosaccharides (**1a**–**1o**, Figure S2), in order to compare our DiBT-MS screening protocol against a previously reported ^19^F-NMR method to determine reaction conversions.^8^ The output from this screen (Figure 2a) is an *m*/*z* selected heat map of the location of the product ion of interest. Each reaction mixture is deposited in a grid format (Figure S1) which provides mass-resolved heat maps allowing easy visual identification of enzyme–substrate pairs with high activity (brightest colored pixels). Due to the isomeric nature of some of the substrates tested in this screen, extracting mass spectral data for a single *m*/*z* value (M-1) (**2a**–**2h**, Table S1, 261.1 Da) is sufficient to view results from 88 reactions (yellow pixels). Alternatively, if substrates (and consequently products) are different in mass (**1i**–**1o**, Table S1), multiple *m*/*z* values may be selected, and the resulting heat maps can be overlaid to generate a single, easy to interpret, image (red, green, and blue pixels). When we compared DiBT-MS heat maps with the results of quantitative ^19^F-NMR analysis (Table S2), we found a semiquantitative correspondence between the two methods (Supporting Information, Figures S3 and S4, with the calculated reaction conversions (%) indicated below the corresponding DiBT-MS spot).

**Figure 3:**
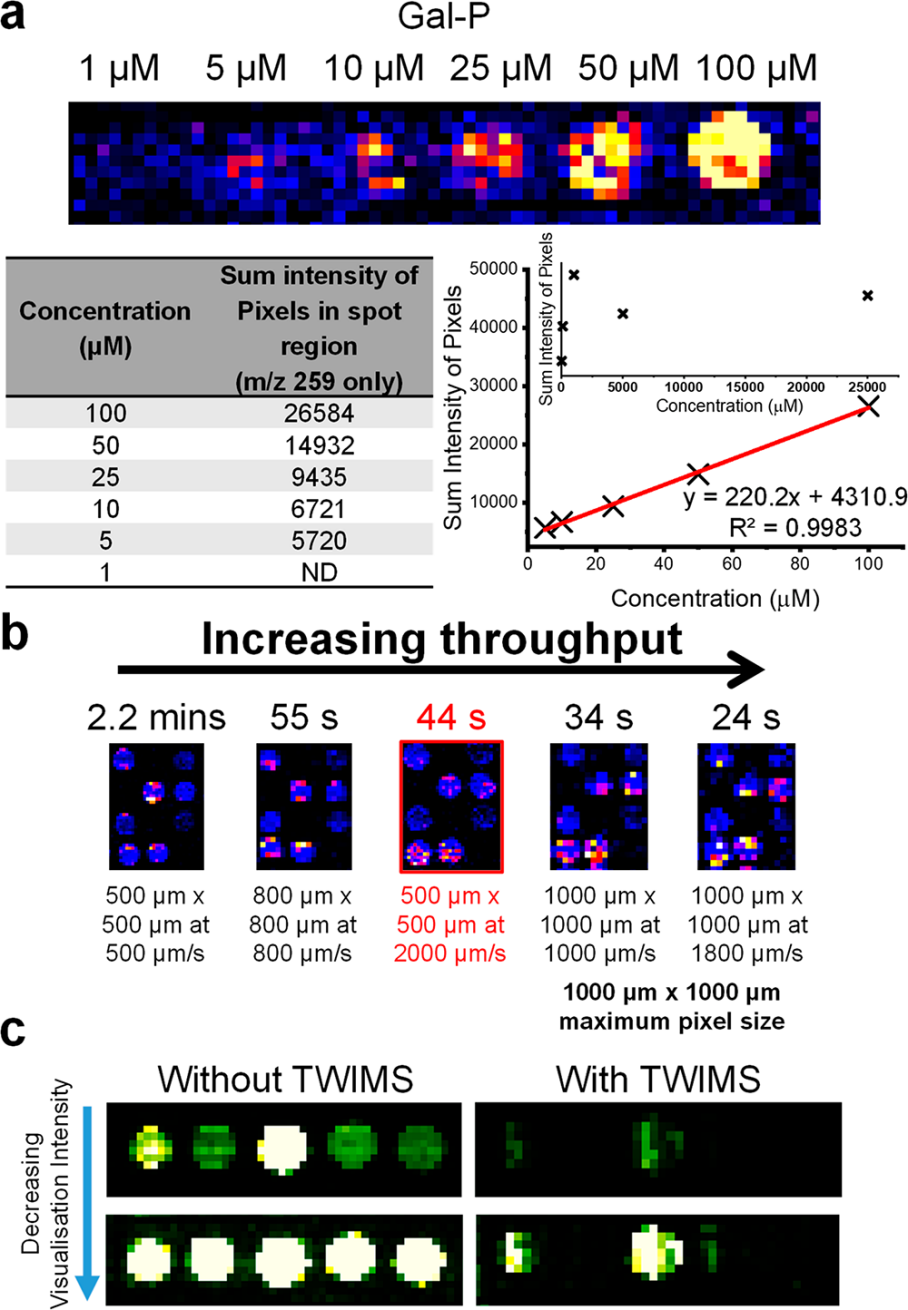
(a) Mass-resolved heat map indicating the limit of detection of six different concentrations of galactose-1-phosphate using a pixel size of 500 μm × 500 μm and a rate of 2000 μm/s. The intensity of each pixel within each concentration spot has been summed and plotted versus the concentration of that spot. The graph shows good linearity between the concentration of each spot and the summed pixel intensity over the range 5–100 μM. Graph insert illustrates saturation of the compound. (b) DiBT-MS analysis of 12 kinase biotransformations at varying pixel sizes and stage speeds. The resulting throughput per sample has been noted. A 44 s/sample throughput has been highlighted as being the optimal conditions for high-throughput screening without compromising data quality. (c) Heat maps obtained when analyzing IRED reactions in 5 different wells of a metagenomic plate both with (right) and without (left) traveling wave ion mobility (TWIMS) enabled within the SYNAPT mass spectrometer. Two visualizations of each heat map are given to show IM filtering of the substrates’ second ^13^C isotope ion from the major product ion (*m*/*z* 208) within the heat maps.

The technique is also amenable to other enzyme classes (Figure 2b,c) with results discussed below. Optimization of the DiBT-MS method gives a screen of superior throughput compared to other analytical techniques (Figure 2d). Further investigation into quantification gave a good linearity of response over 1.5 orders of magnitude and sensitivity down to 5 μM (Figure 3a,b). These limits are in part determined by the stage raster speed and pixel size: higher throughput will decrease analyte sensitivity (Figure 3b). The linearity afforded from summing pixel signal intensities across a sample deposition region gave a high *R*^2^ value of 0.9983 (obtained across a range from 5 to 100 μM, Figure 3a), with saturation occurring for this compound (galactose-1-phosphate) above ∼1 mM (Figure S5).

### Screening of Metagenomic Imine Reductases (Cell-Free Extract)

Among the toolbox of enzymes available for the generation of chiral amines, NADPH-dependent imine reductases (IREDs) have emerged as practical and versatile biocatalysts for both asymmetric imine reduction and reductive amination.^9,10^ Access to these biocatalysts has recently been enhanced by exploring metagenomic sequence space to construct the largest panel of IREDs available to date.^11^

A panel of 384 IREDs was screened for the reduction of dehydrosalsolidine **3** (Figure S6). Formation of salsolidine **4** was observed in *m*/*z* selected heat maps where both substrate and product ions are shown (Figure 2b, see the Methods section for dimensions and grid preparation). The reverse reaction, i.e., oxidation of **4** to **3**, was also assessed using our recently developed colorimetric screen^11^ with good correlation to the DiBT-MS method (Supporting Information, Figure S7). Following DiBT-MS and colorimetric screening, five enzymes were selected for analytical biotransformations in the reductive direction (see Table S3). All enzymes showed excellent conversions (>99%) and excellent enantioselectivities (>99%) where metagenomic IREDs afforded both (*S*)- and (*R*)*-***4**.^10^

### Incorporation of Ion Mobility

From IRED DiBT-MS screening (Figure 2b and Figure S8), the second ^13^C isotope *m*/*z* of the substrate (major ion *m*/*z* 206.1) is *m*/*z* coincident with the major product ion observed (*m*/*z* 208.1). Since this IRED imine to amine biotransformation results in a product only two mass units higher than the substrate, a low-intensity positive hit was identified in every well-plate position “beneath” the genuine biotransformation result. To remove these false positives, traveling wave ion mobility (TWIMS)^12,13^ was employed to “filter” the ^13^C isotope from the product ion. Results from such a DiBT-IM-MS screen are presented in Figure 3c, in which 5 wells of an IRED reaction from a metagenomic plate were analyzed by this method. Removal is possible as the major product ion (amine) and the substrate (imine) present with differing drift times (Figure S9). The heat map data obtained without ion mobility separation are shown alongside one another in Figure 3c to illustrate the removal of the underlying false positive.

### PAL Screening and Directed Evolution Case Study (Whole Cell)

Phenylalanine ammonia lyases (PALs) catalyze the enantioselective addition of ammonia to cinnamic acids to yield l-phenylalanine derivatives. Currently, the available suite of PALs is limited for substrates with multiple electron-donating substituents on the phenyl ring of both amino acid and acrylic acid substrates.^14,15^ As such, we sought to use DiBT-MS to identify PALs able to accept electron-rich cinnamic acid derivatives (Figure S10 and Table S4) that are present in lignocellulosic biomass degradation (e.g., *p-*coumaric, ferulic, sinapic acid). These phenylalanine derivatives are key building blocks for biologically active molecules and active pharmaceutical ingredients (APIs) such as the anti-Parkinson drug L-DOPA.^16^

Initially, the activities of a panel of wild-type PALs were compared by HPLC (Table 1, full data set in Table S5), including enzymes from published sources and metagenomic origin, against a broad panel of methoxy-substituted arylacrylic acids **5a**–**5u** (Supporting Information, Data Set S1). PbPAL from *Planctomyces brasiliensis*(17) and metagenomic AL-11 (accession number: MW026687) accepted substrates **5a**–**5k** similarly; however, AL-11 showed much greater substrate conversions to the amino acid products **6a**, **6d**, **6f**, **6g**, and **6h**. It is worth noting that AL-11 revealed low activity with an electron-donating group solely at the *para* position (**5c**, **5j**) compared to PbPAL.

**Table 1:**
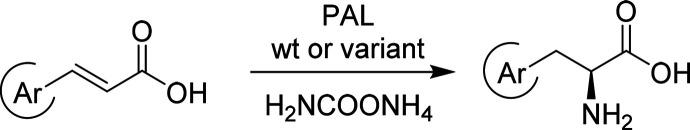
AL-Catalyzed Hydroamination of Various Electron-Rich Substituted Cinnamic Acids

substrate	Ar substituents	PbPAL^a^ conversion (%)	AL-11^a^ conversion (%)
**5a**	2-MeO	27	68
**5b**	3-MeO	92	61
**5c**	4-MeO	8	5
**5d**	3-MeO-4-F	79	91
**5e**	3-MeO-4-Cl	91	59
**5f**	3-F-4-MeO	40	81
**5g**	3-Cl-4-MeO	38	87
**5h**	2-Et	48	91
**5i**	3-Et	30	76
**5j**	4-Et	48	5
**5k**	2,3-(OCH_2_O)	92	98
**5l**	3,4-(OCH_2_O)	3	11
**5m**	2,3-(MeO)_2_	<1	5
**5n**	2,4-(MeO)_2_	<1	<1
**5o**	3,4-(MeO)_2_	<1	92
**5p**	3,4-(MeO)_2_-6-F	<1	96
**5q**	3,5-(MeO)_2_	<1	98
**5r**	2,4,6-(MeO)_3_	<1	<1
**5s**	3,4,5-(MeO)_3_	<1	97
**5t**	2,3,4-(MeO)_3_	<1	4
**5u**	2,4,5-(MeO)_3_	<1	73

a Determined on nonchiral reverse-phase HPLC.

Moreover, AL-11 exhibited remarkable activity with di- or trimethoxycinnamic acids (**5o**–**5q**, **5s**), which are generally inactive in the known PAL sequence space.^18^ It is reasonable to suppose that the low activity is due to not only the presence of electron-donating groups but also a complex network of stereoelectronic effects which mean that the substituents are more weakly bound in the enzyme active site.

Although the hydroamination reaction does not proceed with naturally occurring lignin monomers such as ferulic acid, to our surprise, excellent conversion (92%) and perfect enantioselectivity (>99% ee) could be achieved with the alkylated 3,4-dimethoxycinnamic acid **5o** as a substrate, to generate l-veratrylglycine (a key building block for a variety of biologically active molecules such as L-DOPA and anticancer agents^19^). Closer inspection of the sequence alignments revealed slight active-site deviation from previously identified “selectivity residues” such as the otherwise conserved amino acid residue L90 in PbPAL which corresponds to A80 in AL-11. This variation was predicted to allow accommodation of the large *m*-MeO substituents in the active site of AL-11. Similar evidence was discovered that introducing a leucine to alanine point mutation in a related phenylalanine aminomutase enzyme from *Taxus canadensis* (TcPAM) increased the activity of *m*-Me compounds.^20^

**Figure 4:**
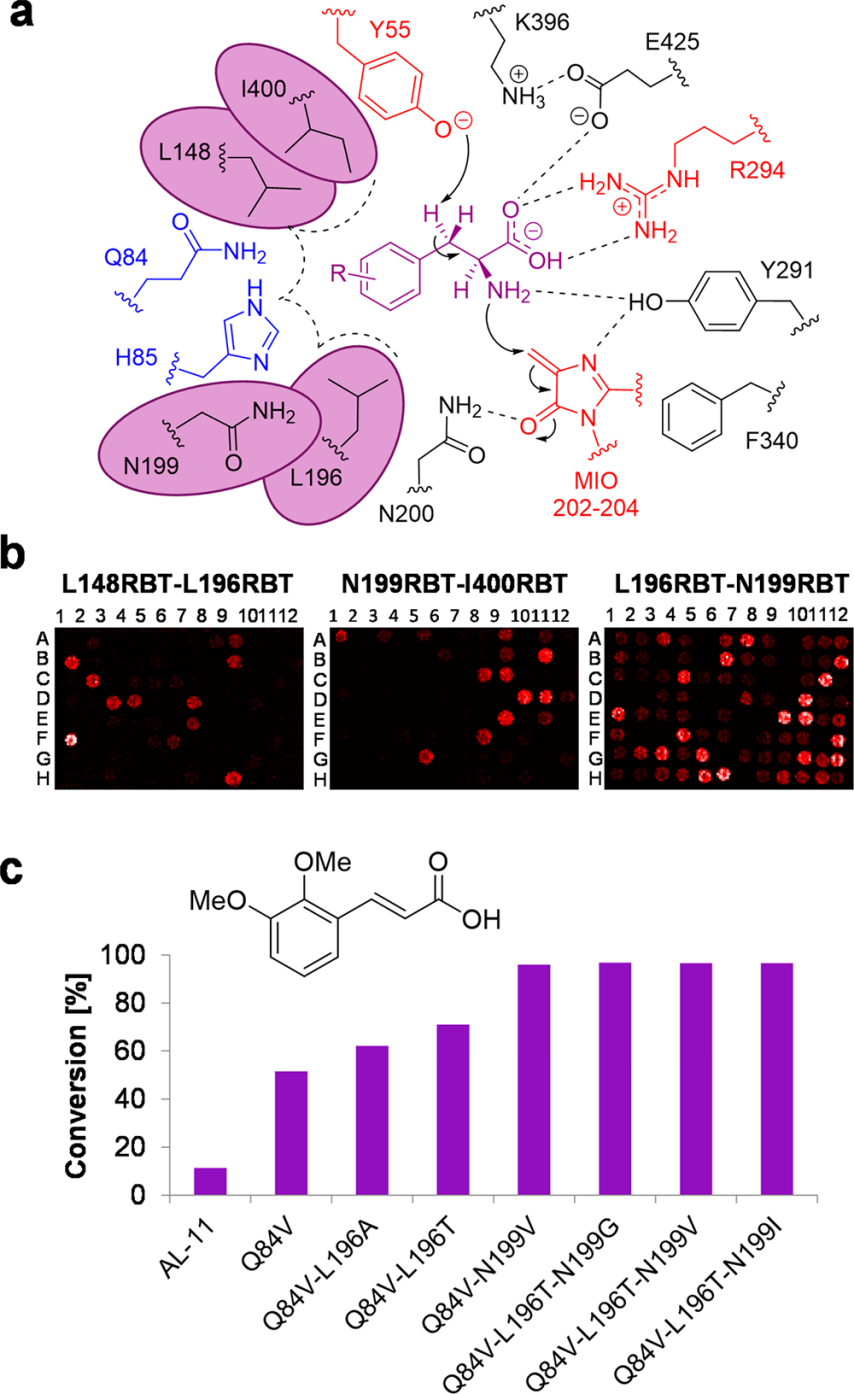
(a) Homology-based active site model of AL-11 enzyme highlighting the amino acid residues conferring selectivity and reactivity from the structure of AvPAL (PDB: 5LTM)^15^ with selectivity residues highlighted (blue). The post-translationally modified 3,5-dihyro-5-methylene-4*H*-imidazol-4-one (MIO) ring and the neighboring polar side chains are shown (black). Residues circled (purple) were selected for reduced degenerate codon library (RBT) creation. (b) DiBT-MS screening results for library A (L148RBT/L196RBT), library B (N199RBT/I400RBT), and library C (N196RBT/N199RBT) in a 96-well plate format as indicated. (c) Analytical scale biotransformations using **5m** as a substrate with AL-11 and variants identified during DiBT-MS screening. Conversion values (%) determined by reverse-phase HPLC.

To improve the activity of the PAL enzymes with *o*-MeO substituents (**5m**, **5r**, **5t**), which gave an overall conversion of <5%, we performed a blast sequence alignment of AL-11 against previously reported PAL homologues and found highly conserved active site residues surrounding the electrophilic MIO catalytic ring moiety. An energy-minimized homology model of AL-11 was constructed from *Anabaena variabilis* AvPAL^15,21^ (PDB: 5LTM) with a 3,4-dimethoxycinnamic acid **5o** ligand docked in the hydrophobic enzyme active site (Figure 4a and Figure S11).

The first engineering approach involved the point mutation of the large polar amino acid residue Q84 to smaller hydrophobic groups, which widens the substrate cavity in close proximity to the *p*-substituents that gave rise to variants such as Q84A, Q84I, and Q84V. The second approach attempted to mimic the activities of the related aromatic ammonia lyase family members^23,24^ PALs/TALs/HALs that are known to contain large hydrophobic groups (Q84F, Q84Y) or a positively charged side chain (Q84H). Of the variants tested under the same condition as WT using the selected test substrate **5m**, the variant Q84V had greater than 7-fold conversion (37%) to the unnatural amino acid product. Other point mutations were tested on the amino acid residue H85, which proved to be overall detrimental to product conversions, even abolishing WT activity with all substrates screened (Figure 2c and Figure S12).

In addition to the Q84V variant and to limit the screening efforts, we created combinatorial libraries targeting neighboring active site residues N199, L196, L148, and I400 surrounding the cinnamic acid aromatic moiety using a degenerate codon set RBT (coding for the 6 amino acids T, S, I, G, A, V). Double mutation libraries were designed and gave rise to a total number of 36 possible amino acid combinations; this required screening approximately 106 clones to achieve 95% coverage.^25^

DiBT-MS screening of library A (L148RBT/L196RBT) (Figure 4b) gave few hits and revealed the crucial importance of conserving the L148 amino acid. In accordance with a previous report using PcPAL a large decrease in product conversion was shown in *o-*MeO substituents with the complementary conserved L206 amino acid residue mutated to valine.^14^ By contrast, the mutational changes within the L196RBT library gave rise to conversions up to 62% and 70% from variants L196A and L196T, respectively.

The screening of library B (N199RBT/I400RBT) (Figure 4b) detected hits where point mutations were present at the conserved amino acid residue N199. Activity was determined with mutated variants to the smaller hydrophobic side chain residues (G, V, I, T), but the N199V mutation was the most prevalent and noticeable in the DiBT-MS screening. Interestingly, other homologues in the aromatic ammonia lyase family exhibit this corresponding valine amino acid residue.^23,27^ In the same manner, variants of the highly conserved I400 residue were not detected presumably due to the critical structural role of this residue in the enzyme active site.

After identifying amino acid hotspots from our two previous mutagenic libraries, we sought to recombine L196RBT and N199RBT to create library C (Figure 4b) containing the best possible combinations. Most hits were detected in library C with the predominantly featured L196T mutation in combination with mutated residue N199I, N199G, or N199V which gave >95% conversion of **5m** to the corresponding product (Figure 4c). In addition, we further explored trisubstituted cinnamic acids **5t** and **5u** containing the disubstitution motif of **5m** against our selected “hits” and found variants that gave >99% conversion to the corresponding l-phenylalanine derivatives (Figure S13).

To demonstrate the practical applicability of these enzymes, we performed preparative scale biotransformations with the best variants for 7 of the most challenging substrates considered in the panel (**5m**, **5o–q**, and **5s–u**). The corresponding amino acid products were isolated in pure form by adsorption on ion-exchange resin in good isolated yields (68–90%) and >97% ee (Supporting Information, Data Set S2).

## Discussion and Outlook

DiBT-MS screening is found to be highly effective across different enzyme classes (kinases, imine reductases, and phenylalanine ammonia lyases) presented in different formats (purified enzymes, cell-free lysates, and whole cell reactions) with a screening time of 42–50 s per sample (Figure 2d). This suggests that the method will be readily adaptable to many more reaction families, as long as the products are detectable as ions, and we here discuss this in the context of traditional and other emerging techniques.

Although full quantification by DESI-MS has not been performed within this work, the capability for semiquantitative measurement is certainly apparent when visually comparing heat map intensities with reaction conversion data (%) obtained from alternative analytical techniques (^19^F-NMR and HPLC-(UV)). This method of obtaining data from the positional mass spectra could be further optimized by applying automated and ML approaches now emerging in mass spectrometry imaging;^28,29^ this would likely improve both the linearity of response and the sensitivity—and therefore the throughput. Full reaction conversion data sets for both the kinase and PAL reactions can be found in Figures S3, S4, and S12, respectively. In each case, a brighter spot corresponds with higher reaction conversion allowing mutants with increased activity to be identified for each substrate. The method can detect products with as low as 9% conversion for kinase purified enzyme reactions and down to 3% for the PAL whole cell reactions. Although it may appear that intersubstrate assessments can be made, it should be noted that, due to substrates’ differing ionization efficiencies and affinity to the nylon membrane, care should be taken when making such comparisons.

The fastest throughput afforded by this method corresponds to less than ∼50 s per sample without compromising data quality (Figure 3b), more than 30 times faster than an equivalent NMR screen and with much lower solvent consumption. Some of the fastest colorimetric and fluorescent well plate readers can afford throughputs of 1 well plate every 20 s (76.8 samples/s for a 1536 well plate) but do not have the label-free capability that mass spectrometry affords. If the products (or reactants) of interest are not inherently fluorescent or UV-active, these methods will be inappropriate. Other mass spectrometry platforms that are capable of high-throughput screening include those coupled to fast chromatographic systems and solid-phase extraction to “clean up” the sample before it reaches the mass spectrometer. Although this “clean up” may be desired to optimize the MS data output, it often utilizes excessive amounts of solvent and additional consumables (such as columns and cartridges) which is avoided with DiBT-MS.

Emerging ultra-high-throughput MS coupled technologies, such as droplet microfluidics^30^ and acoustic mist ionization platforms,^31^ are beginning to offer subsecond sample throughputs; however, commercially available instrumentation is still limited and in its infancy in comparison to DESI-MS. We note that the throughput of this method could be further improved through the implementation of robotic pipetting or acoustic dispensing systems to perform the solution preparation steps as demonstrated by Morato et al.^32^ The addition of a slide changer within the DESI-MS instrumentation would also increase the level of automation and throughput within the workflow, allowing more samples to be analyzed consecutively without user intervention.

The inclusion of ion mobility within this workflow (Figure 3c) increases identification confidence with an additional “drift time” feature, characteristic of the product ion. This is of high utility in reduction reactions in which the mass shift between starting material and product differs by an increase in 2 *m*/*z* units. The drift time is also diagnostic and can separate isomeric and isobaric ions, for example, to determine the products of a regioselective reaction.^33^

## Conclusion

The DiBT-MS screening platform offers a facile and rapid method for screening biocatalysts generated via directed evolution and protein engineering strategies. It provides product identification in a mobility-separated readout of activity, in a heat map format which enables quantitative analysis from crude reaction mixtures. The screen is performed without the need for additional purification, solvent extraction, or chemical derivatization for product quantification, which offers advantages over traditional techniques. In particular, this method facilitates screening for *in vitro* activity under harsh reaction conditions, such as the PAL-catalyzed ammonia addition, which requires 4 M ammonium carbamate to thermodynamically drive the hydroamination reaction to the desired products.

## Methods

### Materials

Commercially available reagents were used without further purification. Aldehydes, malonic acid, piperidine, and all other reagents were purchased from Sigma-Aldrich (St Louis, MO) AlfaAesar, or Fisher Scientific. Restriction enzymes, T4 polynucleotide kinase, T4 DNA ligase, Q5 high-fidelity DNA polymerase, and broad range protein marker (12–250 kDa) were purchased from New England Biolabs (Ipswich, MA). *Escherichia coli* DH5α and BL21 (*DE3*) cells were purchased from New England Biolabs (Ipswich, MA). Expression vector pET-28b was purchased from Novagen (Darmstadt, Germany) and was used for gene expression. HPLC filter vials 0.45uM PVDF with a pre-slit cap were bought from Thomson (California, USA). LB broth base including trace elements was supplied by Formedium (Norfolk, UK). Synthesized oligonucleotides were purchased from Eurofins Genomics (Ebersberg, Germany).

### General Methods

^1^H and ^13^C NMR spectra were recorded on a Bruker Avance 400 spectrometer (400.1 MHz) without an additional internal standard. Chemical shifts are reported as δ in parts per million (ppm) and are calibrated against the residual solvent signal.

Reverse-phase HPLC was performed on an Agilent 1200 Series LC system equipped with a G1379A degasser, a G1312A binary pump, a G1329 autosampler unit, a G1316A temperature-controlled column compartment, and a G1315B diode array detector.

### Analysis Surface Preparation

Nylon membranes (Roche, 82 mm diameter) were cut to size, allowing for a 25 mm^2^ area per sample. Cut membranes were fixed to a glass slide with double-sided tape ensuring a flat surface, and sample areas (5 mm × 5 mm) were marked with a scalpel in a grid formation (Figure S1). Excess tape was removed from around the membrane.

### Kinase Biotransformations and ^19^F NMR Analysis

Reactions were carried out in Tris buffer (50 μL, 100 mM, pH 8.0) at 37 °C for 24 h. The mixture contained monosaccharide (8 mM), ATP (10 mM), MgCl_2_ (5 mM), and GalK or NahK (6 μg, final kinase concentration 0.12 mg mL^–1^). The presence of the sugar-1-phosphate product was determined by HRMS, and the conversion was determined quantitatively by ^19^F NMR, according to the following method. Samples (50 μL) were diluted with MeOH/H_2_O (1:1, 450 μL) and centrifuged (10 000*g*, 5 min) to remove any insoluble matter. The solution was transferred to a 5 mm NMR tube, with a sealed glass capillary tube containing D_2_O for locking and referencing. ^19^F NMR spectra were recorded at 25 °C on a Bruker Avance 500 MHz spectrometer (operating at 470 MHz) equipped with a QCI-F cryoprobe. Conversions were determined by taking the relative integration of the corresponding resonances of the starting material and product, which were typically baseline-separated.^8^

For DiBT screening, reactions were first diluted with MeOH/H_2_O (1:1, 300–450 μL). 0.5 μL of each solution was transferred onto a preprepared nylon membrane grid mounted on a glass slide as detailed above and allowed to air-dry before analysis.

### Whole-Cell Biotransformations (PALs)

Wild-type AL-11 and variants were transformed into *E. coli* BL21 (*DE3*) for yielding *E. coli* BL21 (*DE3*) pET28b-AL-11. A single colony was selected and grown in a 3 mL overnight starter culture supplemented with 50 μg mL^–1^ kanamycin at 37 °C at 200 rpm. The freshly prepared starter culture was used to inoculate 500 mL of LB-based autoinduction media containing 50 μg mL^–1^ kanamycin in 2 L baffled flasks at a rotary shaking rate of 200 rpm at 18 °C for 72 h. The cells were harvested by centrifugation (4 °C, 3250*g*, 20 min). The cells were resuspended with KP_i_ buffer (100 mM, pH 8.0) and harvested again by centrifugation (4 °C, 3250*g*, 20 min). The cell pellet was subsequently aliquoted and stored at −20 °C.

The whole cell biotransformation was conducted by resuspending 50 mg mL^–1^ cell paste in an appropriate volume of ammonium carbamate solution (4 M, pH ∼ 9.9, unadjusted) supplemented with the relevant arylacrylic acid **5a**–**5u** at the required concentration (5 mM for screening, 10–20 mM for preparative applications). All biotransformations were performed at 30 °C with agitation of 250 rpm. After a 24 h incubation period, the samples were centrifuged (5 min, 13 000 rpm) to remove cell debris. The crude supernatant was then dissolved in 50% MeOH (v/v), and 500 μL was transferred to a 0.45 mm filter vial for analysis by HPLC with the remaining solution reserved for analysis.

Conversion values for the PAL reactions of arylacrylic acids **5a**–**5u** were determined by HPLC on a nonchiral reverse-phase Zorbax C-18 Extend column (50 mm × 4.6 mm × 3.5 μm, Agilent), flow rate 1.0 mL min^–1^, temperature 40 °C, detection wavelength 210 nm. Mobile phase: aq NH_4_OH 0.1 M pH 10.0/MeOH, 9:1. For DESI-MS analysis, 0.5 μL of each solution was transferred onto a preprepared nylon membrane grid mounted on a glass slide as detailed above and allowed to air-dry before analysis.

Enantiomeric excess (ee) values for the isolated PAL reaction products were determined by HPLC on a chiral reverse-phase Crownpak CR(+)column (150 mm × 4 mm × 3.5 μm, Daicel), flow rate 1.0 mL min^–1^, temperature 40 °C, detection wavelength 210 nm. Mobile phase: aq HClO_4_ 1.14% w/v/MeOH, 85:15.

Preparative scale PAL reactions were performed as described above for analytical scale reactions, with an increased substrate concentration (10–20 mM) and addition of DMSO (5% v/v) as a cosolvent. Products were isolated by adsorption on ion exchange Dowex 50WX8 resin and elution with ammonium hydroxide solution (as described previously^17^).

### Screening of Metagenomic IREDs

A 384-well flat-bottomed plate containing a 0.5 mg CFE plate was acclimatized to room temperature. Each well was then resuspended to 10 mg mL^–1^ in 0.1 M Tris-HCl pH 8.0. To a 384-well deep-well plate (Corning), the following solution was dispensed: 10 mM 6,7-dimethoxy-1-methyl-3,4-dihydroisoquinoline, 50 mM glucose, 0.5 mM NADP^+^, 0.5 mg mL^–1^ GDH (CDX-901), and 4 mg mL^–1^ IRED lysate, made up to a total reaction volume of 50 μL in 0.1 M Tris-HCl pH 8.0. The plate was then sealed and centrifuged (1000 rpm, 1 min). The 384-deep-well plate was then incubated at 30 °C for 24 h at 1000 rpm. The reaction was then quenched with the addition of 50:50 MeOH/H_2_O and centrifuged (1000 rpm, 1 min) before DiBT-MS screening. 0.5 μL from each well was transferred onto a preprepared nylon membrane grid mounted on a glass slide as detailed above and allowed to air-dry before DiBT-MS analysis.

### Singular IRED Biotransformations

Stock solutions of the IREDs were prepared (pIR-2, pIR-9, pIR-170, pIR-204, and pIR-364) by weighing out crude lyophilized cell-free extract and rehydrated to a concentration of 10 mg mL^–1^ in 0.1 M Tris-HCl buffer pH 8.0. Biotransformations were performed on a 500 μL scale. The reaction mixture contained 10 mM dehydrosalsolidine **3** (6,7-dimethoxy-1-methyl-1,2,3,4-dihydroisoquinoline), 50 mM d-glucose, 0.5 mM NADP^+^, 0.5 mg mL^–1^ GDH (Codexis-901), 4 mg mL^–1^ IRED lysate, or 1 mg mL^–1^*Asp*RedAm (IMAC grade purified); the reaction volume was made up to 500 μL in 0.1 M Tris-HCl buffer pH 8.0. Reactions were incubated at 30 °C for 24 h. Reactions were quenched by the addition of 50 μL of 10 M NaOH and extracted twice with 750 μL of *tert*-butyl methyl ester. The organic fractions were combined and dried by MgSO_4_ and analyzed by HPLC on chiral stationary phase.^10^

### 384-Well IRED Colorimetric Screen

A Mastermix reagent was made up to 25 mL, containing 0.125 mg mL^–1^ INT and 10 mM amine substrate in Tris-HCl (0.1 M) adjusted to pH 9.0. 50 μL of the Mastermix was aliquoted to each well of the plate. The plate was then spun down (1000 rpm, 1 min) and the 384-well pierceable seal (4titude, Surrey, UK) removed. The plate was incubated (in the dark) at 30 °C for 24 h. An absorbance reading at λ = 490 nm was taken at 0, 1, 4, and 24 h. False positives were detected in wells B02, D08, F12, H21, I02, I07, K16, O01, O08, and P17 from the reaction of the enzyme with INT to INT-formazan as previously described elsewhere.^11^ The same procedure was followed for the 384 IREDy-to-go blank plate, except no amine was added to the Mastermix.

### Instrumentation

All DiBT-MS experiments were performed on a SYNAPT G2 Si mass spectrometer (Waters Corporation, Manchester, UK) coupled with a DESI 2D Omni spray ion source (Prosolia, Indianapolis, IN).

### DESI Source

DESI solvent was composed of a methanol–water mixture (49:1) at a flow rate of 2.5 μL min^–1^. Nitrogen gas flow (3 bar) and a capillary voltage of 3.5 kV or 4.0 kV (+ve/–ve respectively) were used to generate an electrospray plume toward the surface of interest. The DESI sprayer position was then adjusted to ensure that optimum signal intensity was achieved from a solution of the product dried on a nylon membrane. The sampling area was defined by HD Imaging software V1.4 (Waters Corporation, Manchester, UK) with pixel size and stage speed defined as per the throughput of the experiment (Figure 3b).

### Mass Spectrometer

The mass spectrometer was operated using MassLynx v4.2 (Waters Corporation, Manchester, UK), with the source cone voltage optimized around 60 V and a source temperature of 150 °C. The trap and transfer cell voltages were set at 4 and 2 V, respectively. All mass spectrometry data were processed (0.2 Da window, 20 000 resolution, top 10 000 peaks) and heat maps analyzed using HD imaging software.

## Supporting Information Available

Supporting Information to accompany this article consists of three files. Supporting Information contains figures that are explicitly referred to in the main text. Supplementary data sets S1 and S2 contain additional characterization data (NMR and HPLC traces) for a number of substrates synthesized for this work and figures therein are not referred to explicitly in the main text. The Supporting Information is available free of charge at https://pubs.acs.org/doi/10.1021/jacsau.1c00027.

Figures that support the development of the DiBT-MS method including a photograph, structures, reaction screening results, linearity analysis, IRED ion mobility data analysis, a homology model, and LC-MS conversion values (PDF)
Data Set S1: Compound characterization data for arylacrylic acid substrates (PDF)
Data Set S2: Compound characterization data for isolated amino acid products (PDF)

## Accession Codes

DNA sequence information for AL-11 has been deposited with GenBank accession no. MW026687.

## Notes

Data availability: source data that support the findings of this study are available from the corresponding author upon request.
