# Supplementary material for: Rapid Screening of Diverse Biotransformations for Enzyme Evolution
Source: JACS Au. 2021 Apr 8;1(4):508–16. doi: 10.1021/jacsau.1c00027 (PMC8154213; doi:10.1021/jacsau.1c00027)
Supplement: Supplementary file 1 — au1c00027_si_001.pdf [file au1c00027_si_001.pdf]

SUPPORTING INFORMATION

Rapid screening of diverse biotransformations for enzyme evolution

Emily E. Kempa, [1, 5] James L. Galman, [1,5] Fabio Parmeggiani, [2] James R. Marshall, [1] Julien Malassis, [3] Clement Q. Fontenelle, [3] Jean-Baptiste Vendeville, [3] Bruno Linclau, [3] Simon J. Charnock, [4] Sabine L. Flitsch, [1,*] Nicholas J. Turner [1,*] and Perdita E. Barran. [1, *]

1. Manchester Institute of Biotechnology, School of Chemistry, The University of Manchester, 131 Princess Street, Manchester, M1 7DN, United Kingdom.

2. Department of Chemistry, Materials and Chemical Engineering "G. Natta", Politecnico di Milano, Via Mancinelli 7, 20131, Milano, Italy.

3. School of Chemistry, University of Southampton, Highfield, SO17 1BJ, Southampton, United Kingdom.

4. Prozomix Ltd., Building 4, West End Ind. Estate, Haltwhistle, Northumberland, NE49 9HA, United Kingdom.

5. These authors contributed equally: Emily E. Kempa, James L. Galman.

* Corresponding authors. perdita.barran@manchester.ac.uk , nicholas.turner@manchester.ac.uk

Contents

DiBT-MS Method Development 4

Figure S1: Photograph of DESI membrane 96 well plate, prepared on a PROSOLIA 96-well Teflon glass slide. The underside of the slide (i.e. non-Teflon coated side) has been used to adhere the membrane that was pre-cut to size (40 mm x 60 mm) with double sided tape. Once adhered, an 8 by 12 grid was etched into the membrane using a scalpel to produce 96 segments of ~25 mm^2 each into to which samples were to be deposited. 4

Kinases 5

Figure S2: Kinase reaction scheme and structures of fluorinated monosaccharide substrates used during biotransformation screening experiments. 5

Table S1: Chemical formula and exact masses of fluorinated substrates and products from kinase reactions. 6

Table S2: NMR conversion tables (%) for kinase reaction from range of substrates and enzymes (from ref.[S1]) 6

Figure S3: Kinase plate 1 – 88 reaction screening (8 substrates x 11 enzymes) DESI-MS heat map of m/z 261. Substrates consisted of 8 isomeric mono-fluorinated monosaccharides and hence the product produced all present at the same m/z value (261). Pixels were defined as 500 µm x 500 µm and analysed at a stage speed of 2000 µm/s. Total analysis time for these 88 samples was 61 minutes, equivalent to 42 s/sample. Reaction conversion data obtained from ^{19}F-NMR analysis has been overlain (white text) below the location of the corresponding reaction spot for comparison. *Note: two inconsistencies between the NMR and DiBT-MS data sets have been identified and indicated in red text below the corresponding reaction spot. These inconsistencies have been attributed to human errors when preparing a large number of samples for the 2 data sets by hand... 7

Figure S4: Kinase plate 2 – 77 reaction screening (7 substrates x 11 enzymes) heat maps (overlain m/z). Substrates consisted of a mixture of di-fluorinated, mono-deoxy-mono-fluorinated and tetra-fluorinated monosaccharides. Substrates are grouped via their expected product m/z, as indicated by the red, green and blue highlighted boxes. All m/z values are monitored simultaneously, and hence separate analyses of this plate for each substrate mass was not required. Pixels were defined as 500 µm x 500 µm and analysed at a stage speed of 500 µm/s. Total analysis time for these 77 samples was 174 minutes, equivalent to 2.26 minutes/sample. 8

Figure S5: Linearity analysis of galactose-1-phosphate and glucose-1-phosphate over 2 concentration ranges, 10 µM – 25 mM and 1 µM – 100 µM. Analysis of DESI-MS heat maps A and B yielded linearity graphs C and D respectively with both illustrating poor linearity for both product compounds over a large concentration range (10 µM - 25 mM). Heat maps E and F correspond to DESI-MS analysis over a smaller range of lower concentration standard solutions for each product with analysis yielding improved linearity over the range 10 µM – 100 µM as is observed in graphs G and H. 9

IREDs 10

Figure S6: IRED reaction schematic including structures of the substrate screened (dehydrosalsolidine) and product detected (salsolidine). 10

Figure S7: Overlay of IRED colorimetric screening results with IRED DESI-MS screening for the oxidation of **4** to **3**. Colorimetric results are denoted as the transparent squares, with the darker green colouring indicating a higher response obtained during the colorimetric screen. Circular regions

indicate locations on the 384-well plate in which DESI-MS screening detected presence of the product ion (m/z 208), with the brighter circular spots indicating a higher response detected. 10

Figure S8: 384-well plate DESI-MS heat maps of IRED starting material (m/z 206, (A), red) and product (m/z 208, (B), green). Both m/z values were monitored simultaneously. DESI-MS pixel sizes were set to 500 µm x 500 µm and analysed using a stage speed of 1500 µm/s. The total DESI-MS analysis time was equivalent to a throughout of 52 s/sample. 11

Table S3: Results of Singular IRED Biotransformations for the reduction of **3** to **4** as determined by HPLC. (HPLC method as per ref [S2]). 12

Figure S9: Additional IRED ion mobility data analysis, illustrating that the starting material and product can not only be separated on their m/z but also TWIMS drift time. This separates the product from the 2nd C13 isotope of the starting material, removing false positive results (see main paper). A) HD Imaging analysis of five wells of the IRED metagenomic plate showing the differing drift times, m/z values and heat maps between product and starting material. B) MassLynx analysis with the ion mobility drift profiles of each m/z value. 13

PALs 14

Figure S10: PAL reaction scheme and structures of substituted cinnamic acid substrates used in PAL whole cell reactions. 14

Table S4: Chemical formula and exact masses of cinnamic acid substrates and phenylalanine derived products used for PAL biotransformation screening. 15

Table S5: The conversions of a panel wild type ammonia lyases analysed by LC-MS.Not detected = n.d. Not tested = NT. 16

Figure S11: Homology model of the active site of AL-11 enzyme with ligand **5o** docked. Mechanism-related residues are shown in cyan, selectivity residues are shown in yellow, library A residues in orange, library B residues in pink, other residues in close contact in green, substrate in black. (A) Top view. (B) Side view. 17

Figure S12: DESI-MS heat map of screening results from PAL whole cell reactions (10 mutants x 15 substrates). The m/z heat maps for each product ion have been assigned differing colours and then collated to obtain a final image indicating locations of product yielding reactions. Reaction conversion data (%) obtained by HPLC-(UV) analysis or each reaction mixture has been overlain (white text) with each reaction location for comparison with DESI-MS results. DESI-MS pixel sizes were set to 500 µm x 500 µm and analysed using a stage speed of 1500 µm/s. This is equivalent to a throughout of 44 s/sample. Variants: 1 = wt (Q84-H85); 2 = Q84F, 3 = Q84H, 4 = Q84I; 5: Q84V; 6: Q84A; 7: Q84Y; 8: Q84Y-H85I; 9: Q84I-H85V; 10: Q84A-H85V. 18

Site-directed mutagenesis for generating AL-11 single and double mutant libraries 19

Figure S13: Conversion values (LC-MS) of additional analytical scale hydroamination reactions with AL-11 and newly identified mutants with trisubsituted cinnamic acid substrates. A) Substrate **5t**. B) Substrate **5u**. 20

DiBT-MS Method Development

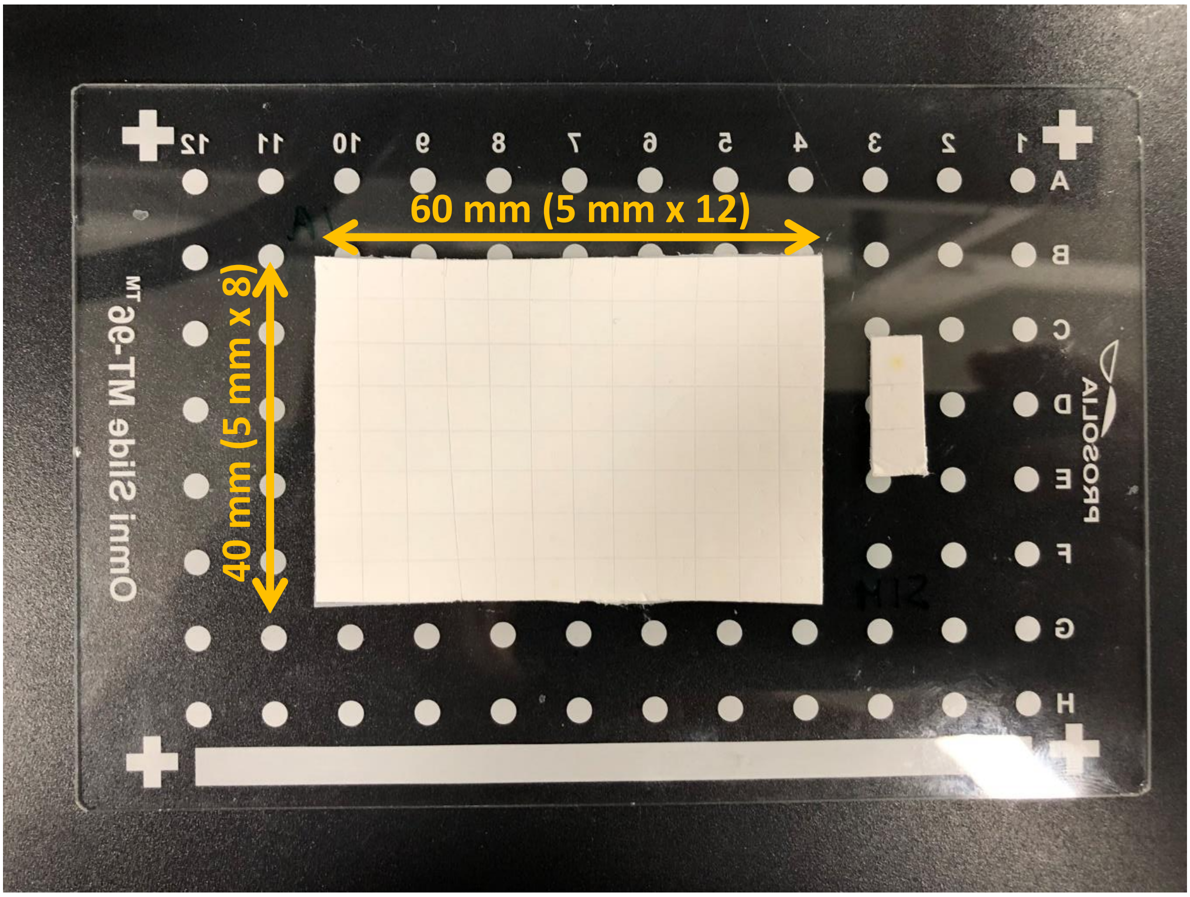

Figure S1: Photograph of DESI membrane 96 well plate, prepared on a PROSOLIA 96-well Teflon glass slide. The underside of the slide (i.e. non-Teflon coated side) has been used to adhere the membrane that was pre-cut to size (40 mm x 60 mm) with double sided tape. Once adhered, an 8 by 12 grid was etched into the membrane using a scalpel to produce 96 segments of ~25 mm^2 each into to which samples were to be deposited.

Kinases

KINASE REACTION

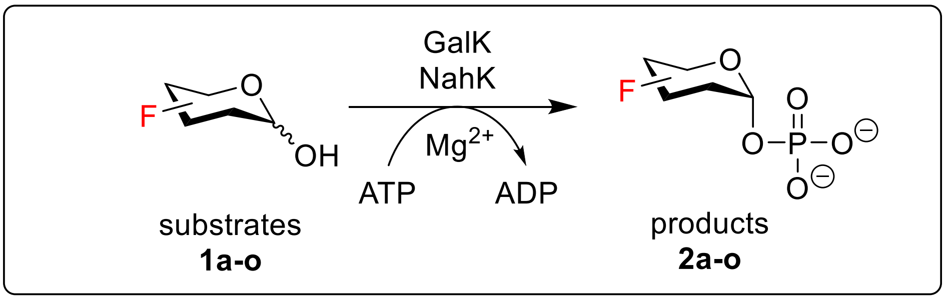

SUBSTRATES

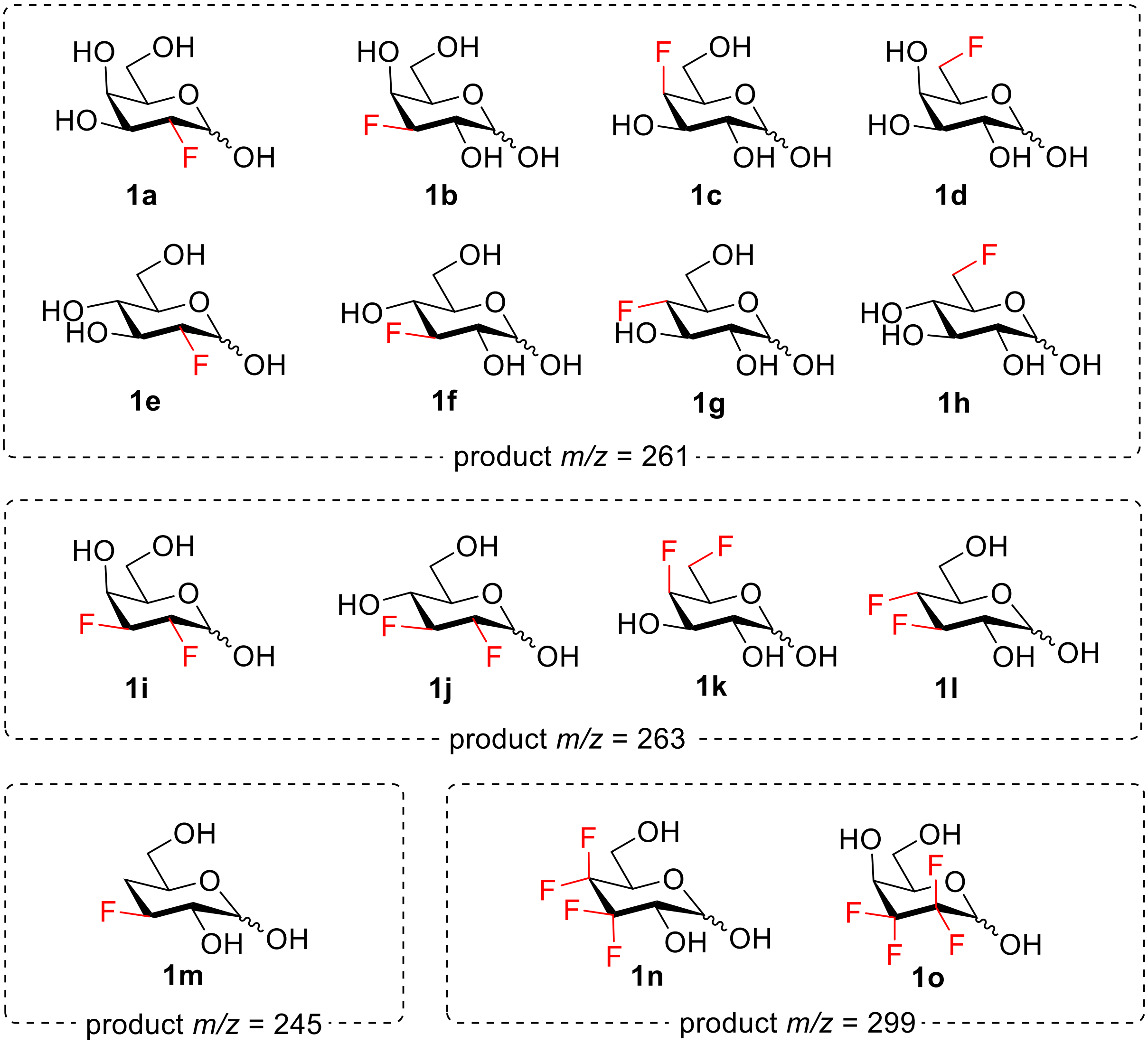

Figure S2: Kinase reaction scheme and structures of fluorinated monosaccharide substrates used during biotransformation screening experiments.

Table S1: Chemical formula and exact masses of fluorinated substrates and products from kinase reactions.

Substrates			Products		
**Subs.**	**Chemical formula**	**Exact mass**	**Prod.**	**Chemical formula**	**Exact mass**
**1a** (Gal2F)	$C_6H_{11}FO_5$	182.058	**2a**	$C_6H_{12}FO_8P$	262.025
**1b** (Gal3F)	$C_6H_{11}FO_5$	182.058	**2b**	$C_6H_{12}FO_8P$	262.025
**1c** (Gal4F)	$C_6H_{11}FO_5$	182.058	**2c**	$C_6H_{12}FO_8P$	262.025
**1d** (Gal6F)	$C_6H_{11}FO_5$	182.058	**2d**	$C_6H_{12}FO_8P$	262.025
**1e** (Glc2F)	$C_6H_{11}FO_5$	182.058	**2e**	$C_6H_{12}FO_8P$	262.025
**1f** (Glc3F)	$C_6H_{11}FO_5$	182.058	**2f**	$C_6H_{12}FO_8P$	262.025
**1g** (Glc4F)	$C_6H_{11}FO_5$	182.058	**2g**	$C_6H_{12}FO_8P$	262.025
**1h** (Glc6F)	$C_6H_{11}FO_5$	182.058	**2h**	$C_6H_{12}FO_8P$	262.025
**1i (**Gal23F$_2$)	$C_6H_{10}F_2O_4$	184.055	**2k**	$C_6H_{11}F_2O_7P$	264.021
**1j** (Glc23F$_2$)	$C_6H_{10}F_2O_4$	184.055	**2l**	$C_6H_{11}F_2O_7P$	264.021
**1k** (Gal46F$_2$)	$C_6H_{10}F_2O_4$	184.055	**2m**	$C_6H_{11}F_2O_7P$	264.021
**1l** (Glc34F$_2$)	$C_6H_{10}F_2O_4$	184.055	**2n**	$C_6H_{11}F_2O_7P$	264.021
**1m** (Gal3F4D)	$C_6H_{11}FO_4$	166.064	**2i**	$C_6H_{12}FO_7P$	246.030
**1n** (Gal3344F$_4$)	$C_6H_8F_4O_4$	220.036	**2o**	$C_6H_9F_4O_7P$	300.002
**1o** (Gal2233F$_4$)	$C_6H_8F_4O_4$	220.036	**2p**	$C_6H_9F_4O_7P$	300.002

Table S2: NMR conversion tables (%) for kinase reaction from range of substrates and enzymes (from ref.[S1])

	**Kinase**										
	**1**	**2**	**3**	**4**	**5**	**6**	**7**	**8**	**9**	**10**	**11**
**1a**	>95	>95	>95	>95	>95	<5	25	20	<5	0	5
**1b**	<5	<5	>95	83	74	0	0	0	0	0	0
**1c**	<5	<5	>95	72	91	29	7	>95	49	11	27
**1d**	<5	<5	>95	74	67	0	0	0	0	0	0
**1e**	0	0	0	0	0	26	64	30	0	0	7
**1f**	0	0	0	0	0	0	0	0	0	0	0
**1g**	0	0	0	0	0	<5	23	37	<5	0	<5
**1h**	0	0	0	0	0	0	0	0	0	0	0
**1i**	0	<5	>95	43	45	0	0	0	0	0	0
**1m**	0	0	0	0	0	<5	11	21	<5	0	<5
**1o**	0	0	12	0	0	0	0	0	0	0	0

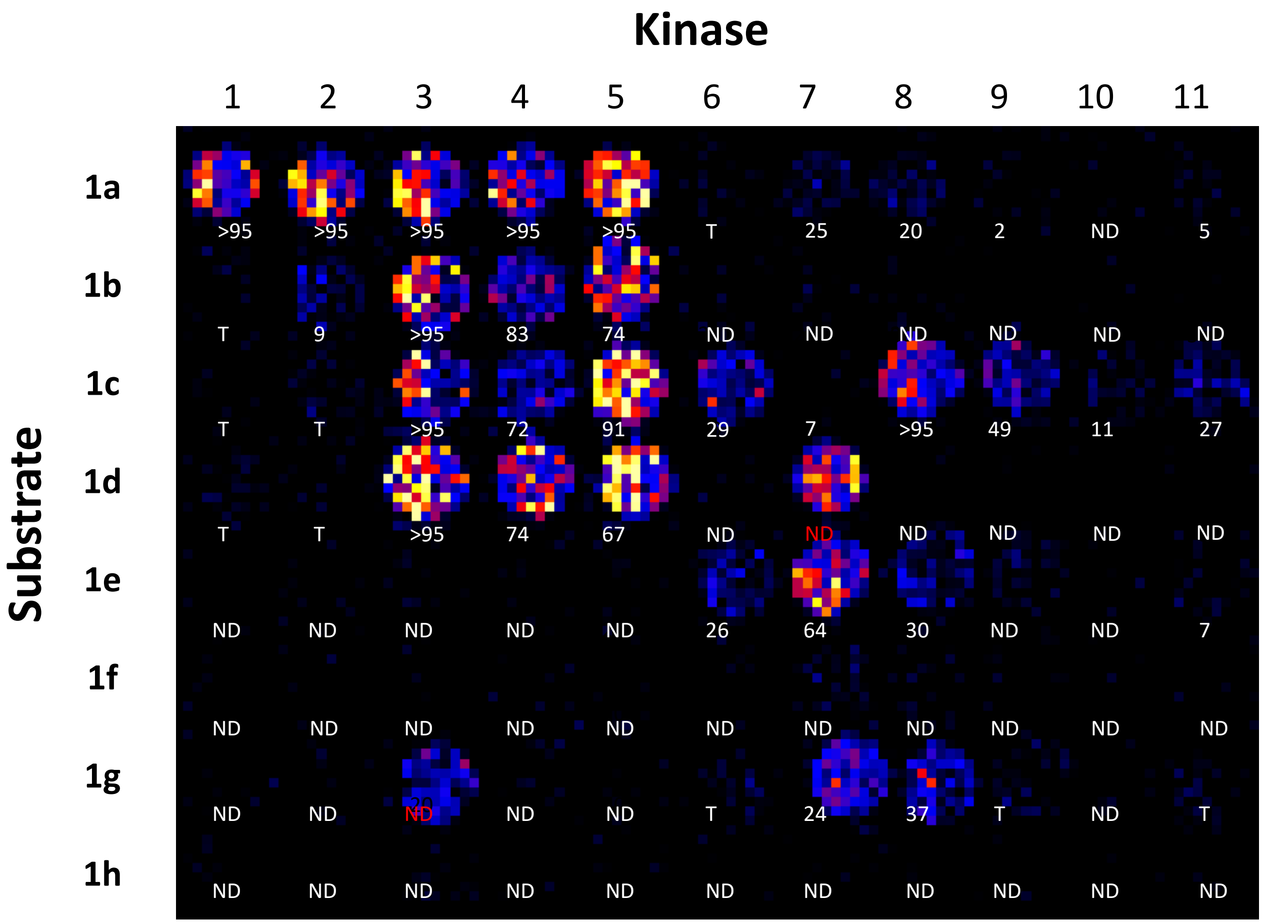

*Figure S3: Kinase plate 1 – 88 reaction screening (8 substrates x 11 enzymes) DESI-MS heat map of m/z 261. Substrates consisted of 8 isomeric mono-fluorinated monosaccharides and hence the product produced all present at the same m/z value (261). Pixels were defined as 500 µm x 500 µm and analysed at a stage speed of 2000 µm/s. Total analysis time for these 88 samples was 61 minutes, equivalent to 42 s/sample. Reaction conversion data obtained from ^{19}F-NMR analysis has been overlaid (white text) below the location of the corresponding reaction spot for comparison. ND = Not detected. T = Trace detected. *Note: two inconsistencies between the NMR and DiBT-MS data sets have been identified and indicated in red text below the corresponding reaction spot. These inconsistencies have been attributed to human errors when preparing a large number of samples for the 2 data sets by hand.*

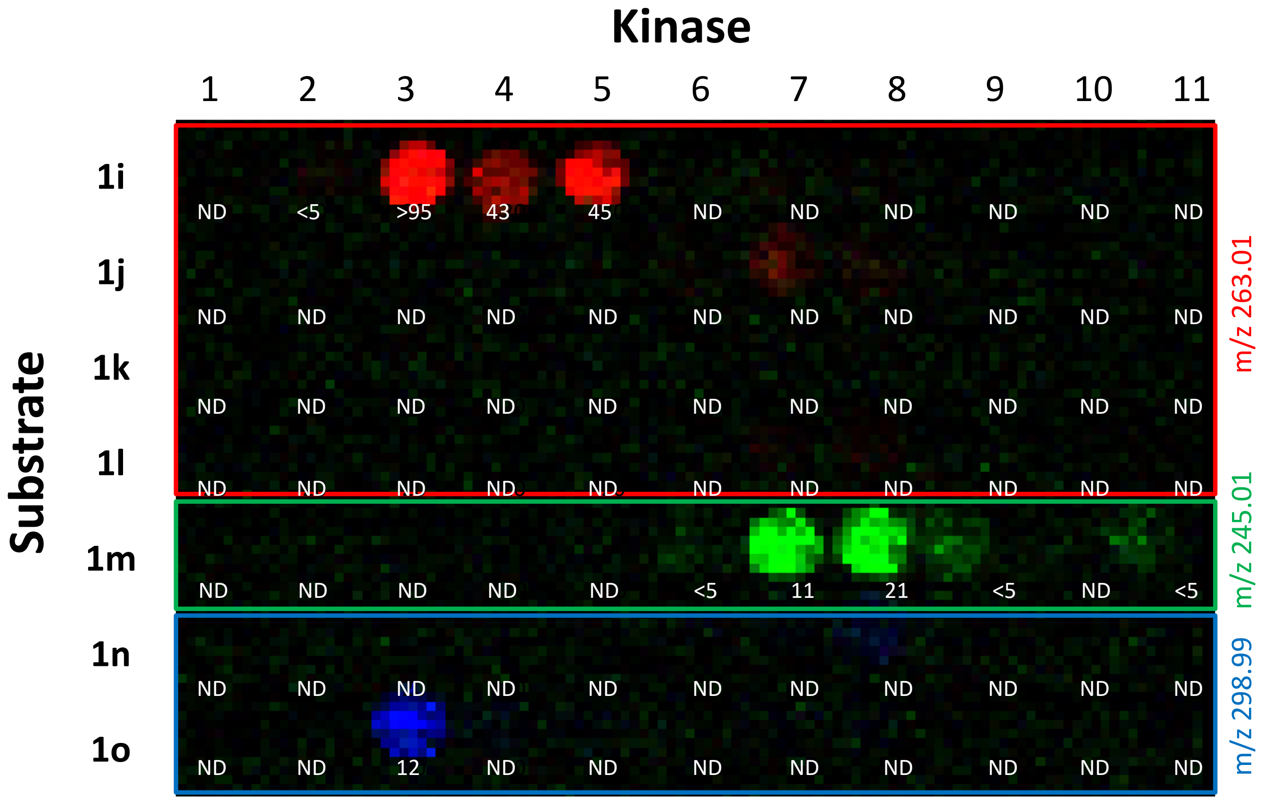

Figure S4: Kinase plate 2 – 77 reaction screening (7 substrates x 11 enzymes) heat maps (overlaid m/z). Substrates consisted of a range of di-fluorinated, mono-deoxy-mono-fluorinated and tetra-fluorinated monosaccharides. Substrates are grouped via their expected product m/z, as indicated by the red, green and blue highlighted boxes. All m/z values are monitored simultaneously, and hence separate analyses of this plate for each substrate mass was not required. Pixels were defined as 500 µm x 500 µm and analysed at a stage speed of 500 µm/s. Total analysis time for these 77 samples was 174 minutes, equivalent to 2.26 minutes/sample. ND = Not detected. Reaction conversion data obtained from ^{19}F-NMR analysis (from reference S1) has been overlaid (white text) below the location of the corresponding reaction spot for comparison.

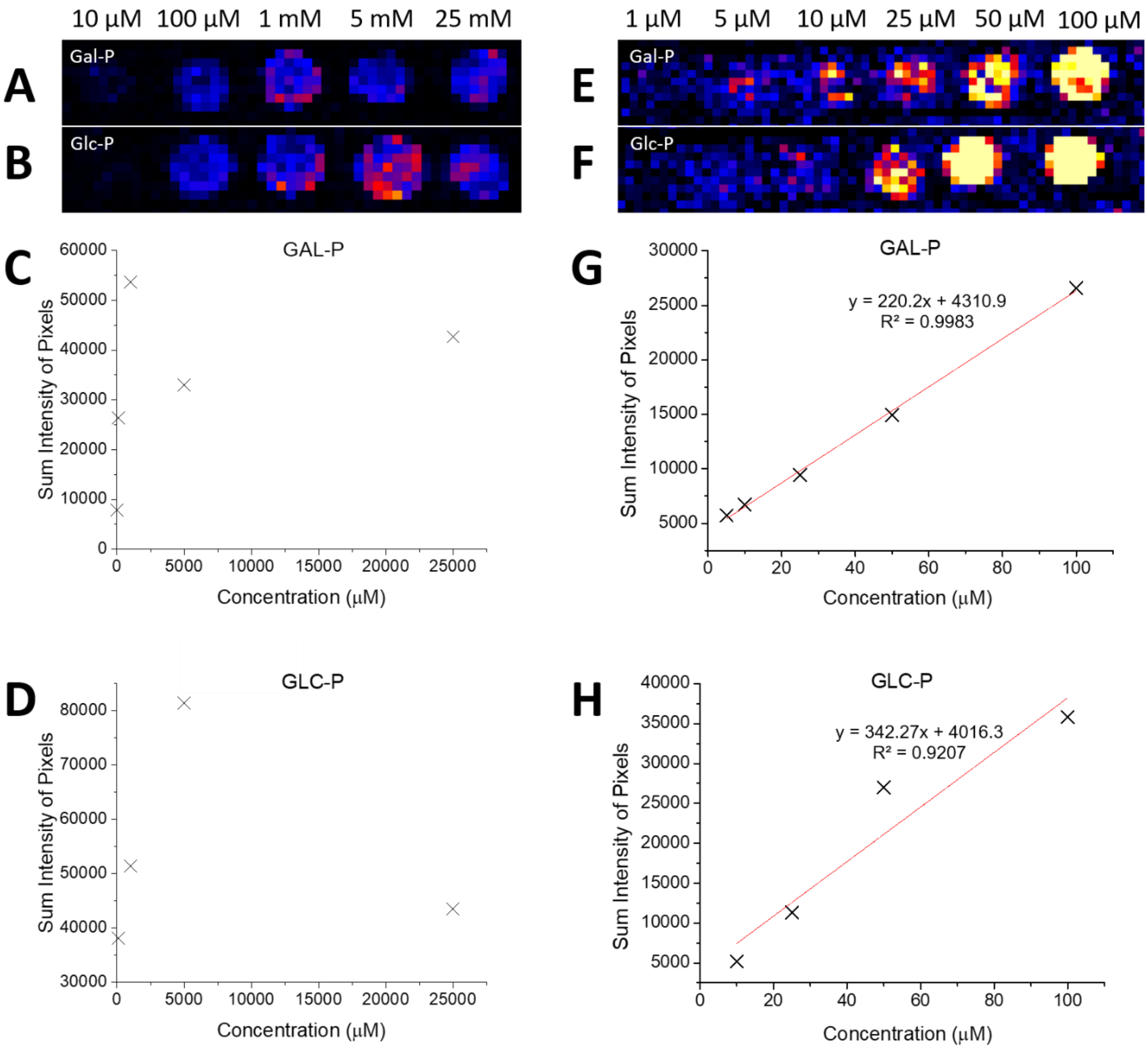

Figure S5: Linearity analysis of galactose-1-phosphate and glucose-1-phosphate over 2 concentration ranges, 10 µM – 25 mM and 1 µM – 100 µM. Analysis of DESI-MS heat maps A and B yielded linearity graphs C and D respectively with both illustrating poor linearity for both product compounds over a large concentration range (10 µM - 25 mM). Heat maps E and F correspond to DESI-MS analysis over a smaller range of lower concentration standard solutions for each product with analysis yielding improved linearity over the range 10 µM – 100 µM as is observed in graphs G and H.

IREDs

IRED REACTION

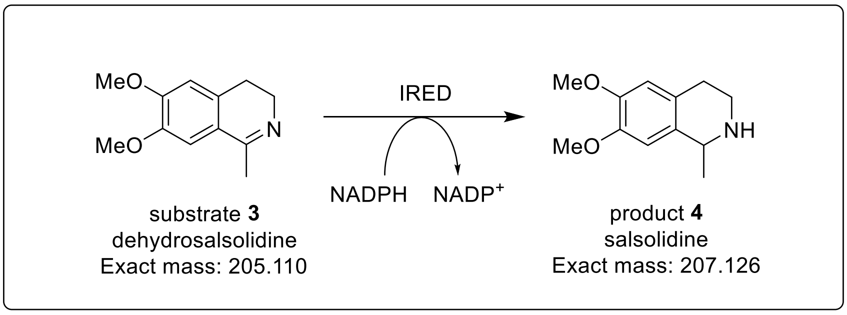

*Figure S6: IRED reaction schematic including structures of the substrate screened (dehydrosalsolidine, **3**) and product detected (salsolidine, **4**).*

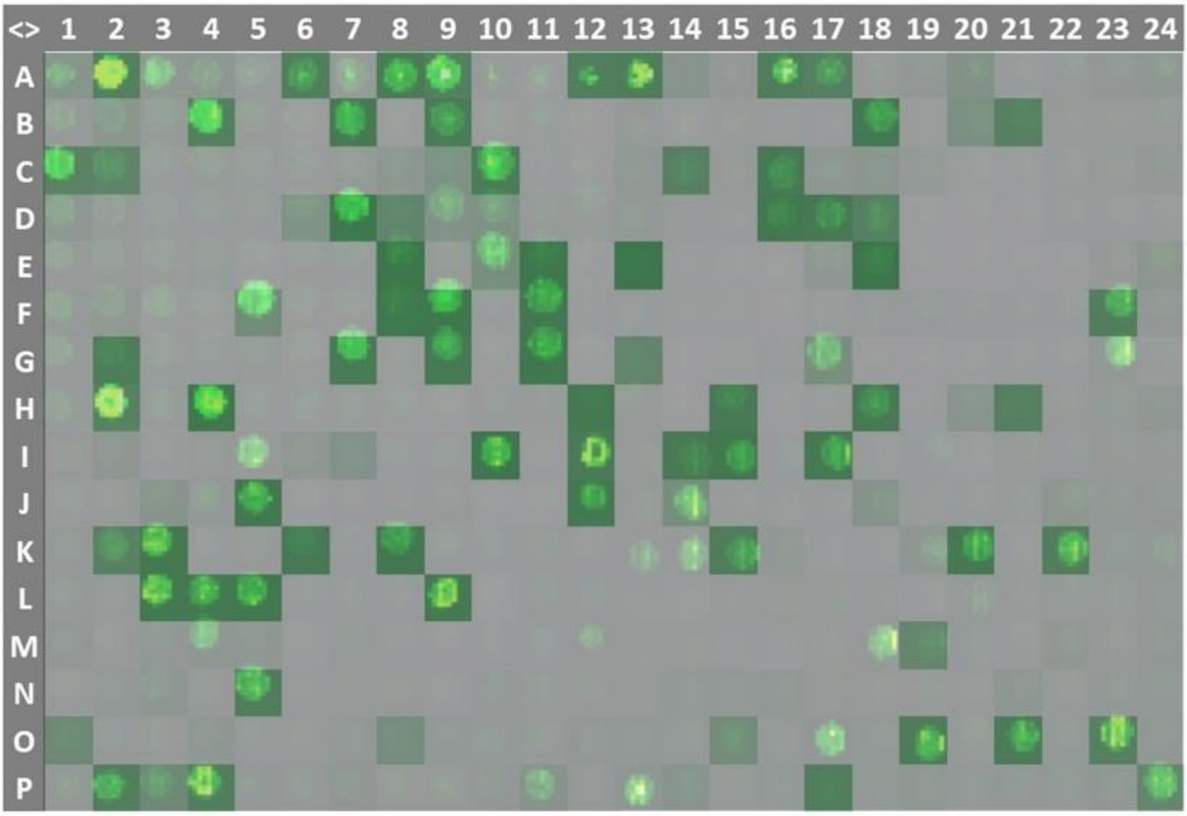

*Figure S7: Overlay of IRED colorimetric screening results with IRED DESI-MS screening for the oxidation of **4** to **3**. Colorimetric results are denoted as the transparent squares, with the darker green colouring indicating a higher response obtained during the colorimetric screen. Circular regions indicate locations on the 384-well plate in which DESI-MS screening detected presence of the product ion (m/z 208), with the brighter circular spots indicating a higher response detected.*

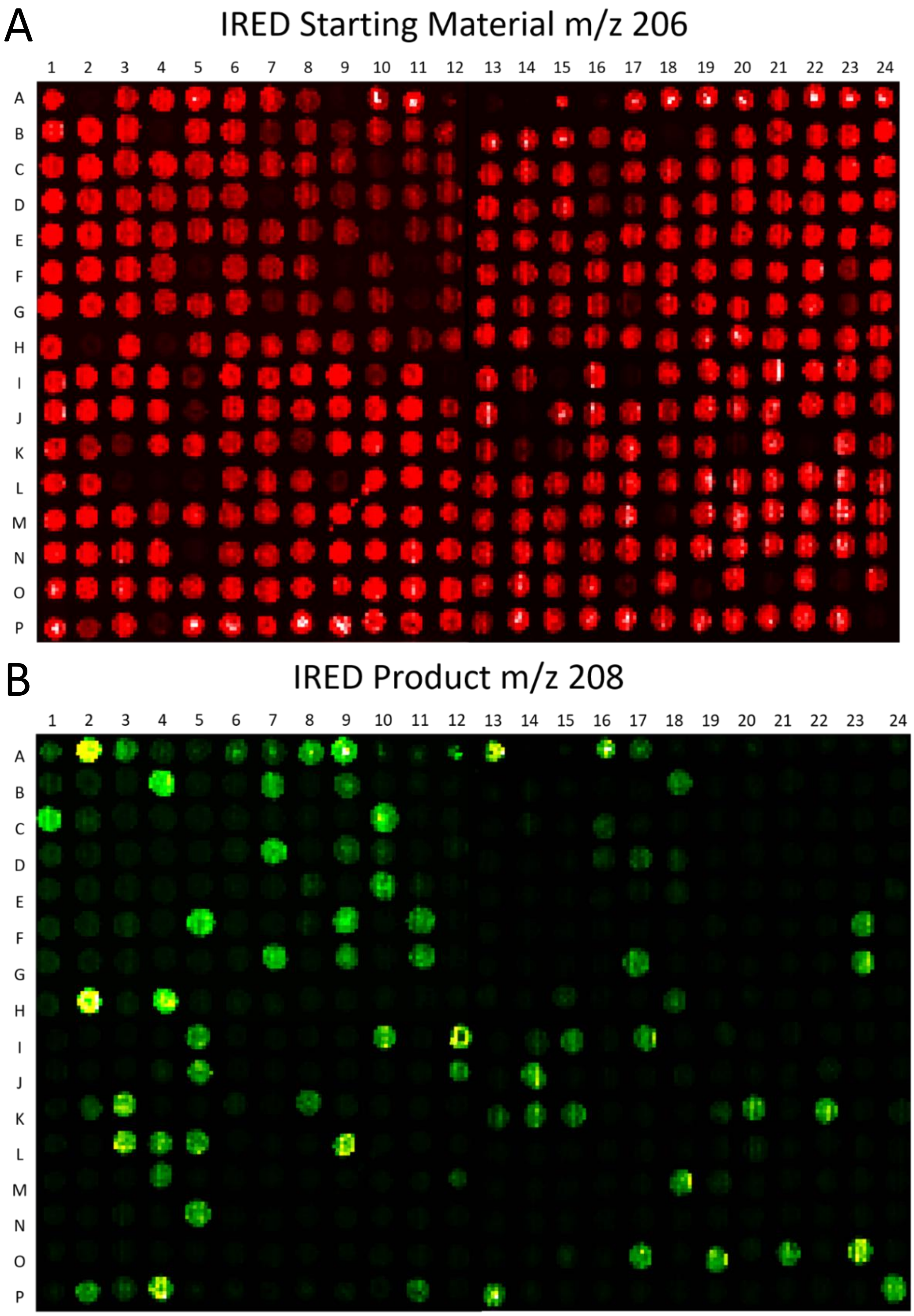

Figure S8: 384-well plate DESI-MS heat maps of IRED starting material (m/z 206, (A), red) and product (m/z 208, (B), green). Both m/z values were monitored simultaneously. DESI-MS pixel sizes were set to 500 µm x 500 µm and analysed using a stage speed of 1500 µm/s. The total DESI-MS analysis time was equivalent to a throughout of 52 s/sample.

*Table S3: Results of singular IRED biotransformations for the reduction of **3** to **4** as determined by HPLC (HPLC method as per ref [S2]).*

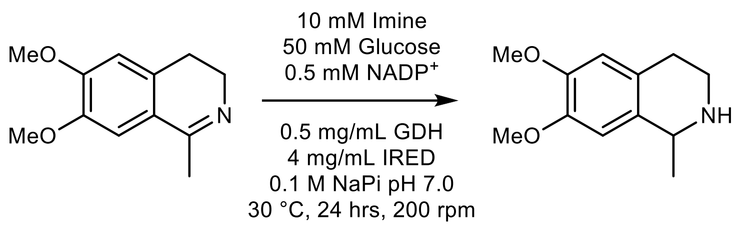

3 **4**

IRED No.	% conversion	% ee
**2**	>99%	>99%^{R}
**9**	>99%	>99%^{R}
**170**	>99%	>99%^{S}
**196**	>99%	>99%^{S}
**364**	>99%	>99%^{S}
***Asp*RedAm (control)**	>99%	>99%^{R}

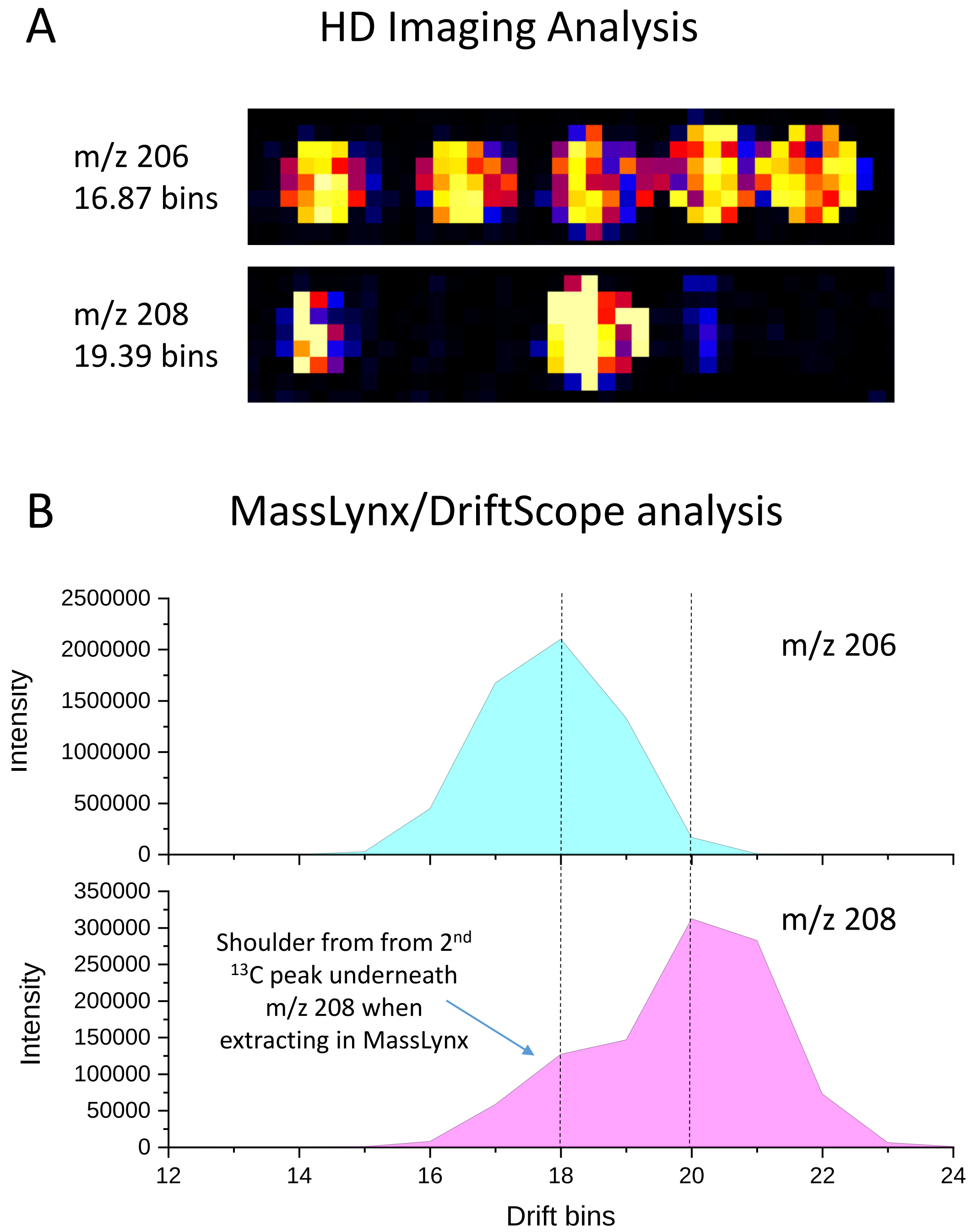

Figure S9: Additional IRED ion mobility data analysis, illustrating that the starting material and product can not only be separated on their m/z but also TWIMS drift time. This separates the product from the 2nd C13 isotope of the starting material, removing false positive results (see main paper). (A) HD Imaging analysis of five wells of the IRED metagenomic plate showing the differing drift times, m/z values and heat maps between product and starting material. (B) MassLynx analysis with the ion mobility drift profiles of each m/z value.

PALs

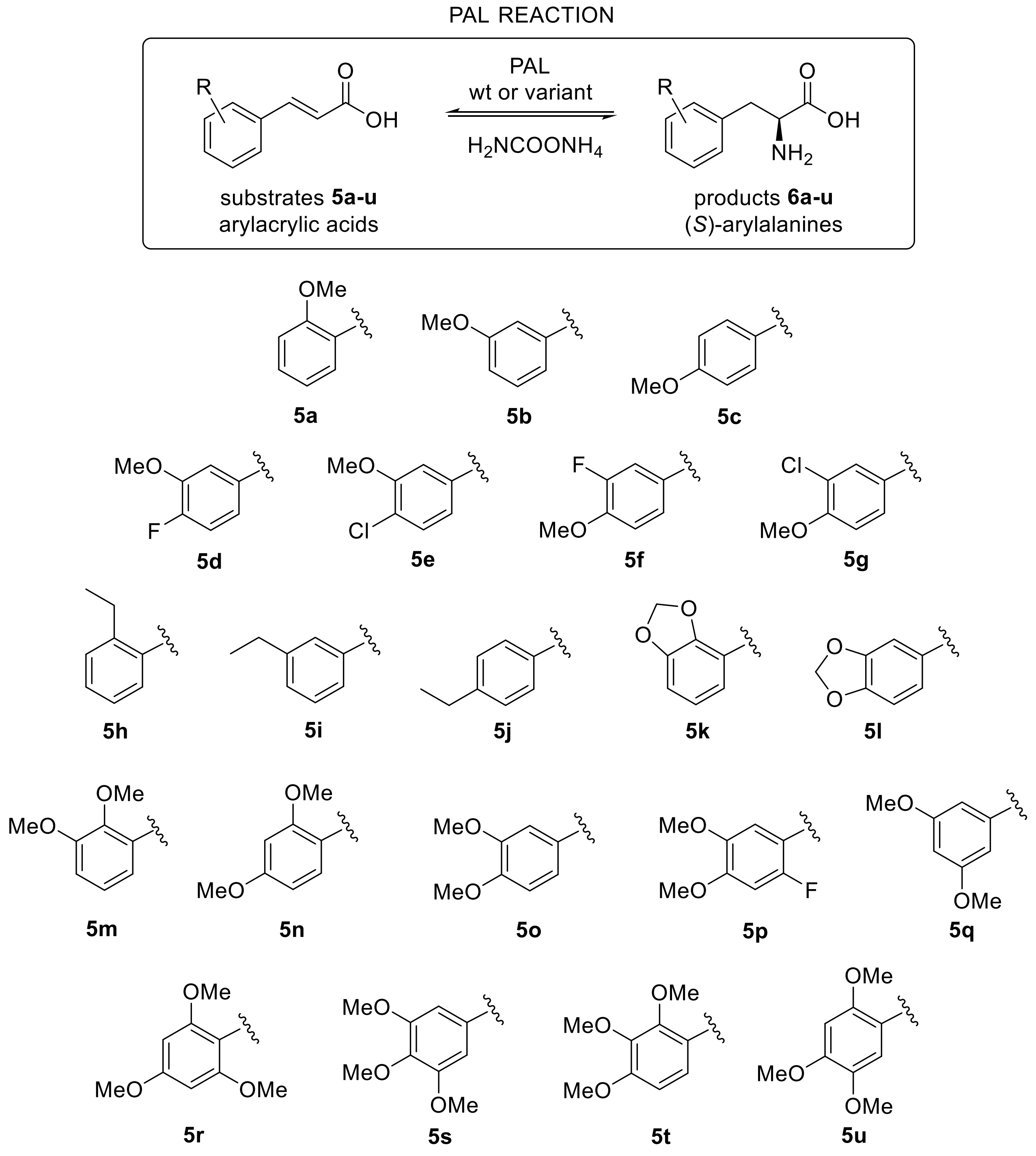

Figure S10: PAL reaction scheme and structures of substituted cinnamic acid substrates used in PAL whole cell reactions.

Table S4: Chemical formula and exact masses of cinnamic acid substrates and phenylalanine derived products used for PAL biotransformation screening.

Substrate	Substituents	Chemical formula	Exact mass	Product	Chemical formula	Exact mass
**5a**	2-MeO	$C_{10}H_{10}O_3$	178.063	**6a**	$C_{10}H_{13}NO_3$	195.090
**5b**	3-MeO	$C_{10}H_{10}O_3$	178.063	**6b**	$C_{10}H_{13}NO_3$	195.090
**5c**	4-MeO	$C_{10}H_{10}O_3$	178.063	**6c**	$C_{10}H_{13}NO_3$	195.090
**5d**	3-MeO-4-F	$C_{10}H_9O_3F$	196.054	**6d**	$C_{10}H_{12}NO_3F$	213.080
**5e**	3-MeO-4-Cl	$C_{10}H_9O_3Cl$	212.024	**6e**	$C_{10}H_{12}NO_3Cl$	229.051
**5f**	3-F-4-MeO	$C_{10}H_9O_3F$	196.054	**6f**	$C_{10}H_{12}NO_3F$	213.080
**5g**	3-Cl-4-MeO	$C_{10}H_9O_3Cl$	212.024	**6g**	$C_{10}H_{12}NO_3Cl$	229.051
**5h**	2-Et	$C_{11}H_{12}O_2$	176.084	**6h**	$C_{11}H_{15}NO_2$	193.110
**5i**	3-Et	$C_{11}H_{12}O_2$	176.084	**6i**	$C_{11}H_{15}NO_2$	193.110
**5j**	4-Et	$C_{11}H_{12}O_2$	176.084	**6j**	$C_{11}H_{15}NO_2$	193.110
**5k**	2,3-(OCH_2O)	$C_{10}H_8O_4$	192.042	**6k**	$C_{10}H_{11}NO_4$	209.069
**5l**	3,4-(OCH_2O)	$C_{10}H_8O_4$	192.042	**6l**	$C_{10}H_{11}NO_4$	209.069
**5m**	2,3-$(MeO)_2$	$C_{11}H_{12}O_4$	208.074	**6m**	$C_{11}H_{15}NO_4$	225.100
**5n**	2,4-$(MeO)_2$	$C_{11}H_{12}O_4$	208.074	**6n**	$C_{11}H_{15}NO_4$	225.100
**5o**	3,4-$(MeO)_2$	$C_{11}H_{12}O_4$	208.074	**6o**	$C_{11}H_{15}NO_4$	225.100
**5p**	3,4-$(MeO)_2$-6-F	$C_{11}H_{11}O_4F$	226.064	**6p**	$C_{11}H_{14}NO_4F$	243.091
**5q**	3,5-$(MeO)_2$	$C_{11}H_{12}O_4$	208.074	**6q**	$C_{11}H_{15}NO_4$	225.100
**5r**	2,4,6-$(MeO)_3$	$C_{12}H_{14}O_5$	238.084	**6r**	$C_{12}H_{17}NO_5$	255.111
**5s**	3,4,5-$(MeO)_3$	$C_{12}H_{14}O_5$	238.084	**6s**	$C_{12}H_{17}NO_5$	255.111
**5t**	2,3,4-$(MeO)_3$	$C_{12}H_{14}O_5$	238.084	**6t**	$C_{12}H_{17}NO_5$	255.111
**5u**	2,4,5-$(MeO)_3$	$C_{12}H_{14}O_5$	238.084	**6u**	$C_{12}H_{17}NO_5$	255.111

Table S5: The conversions of a panel wild type ammonia lyases analysed by LC-MS. Not detected = n.d. Not tested = NT. All conversion values are measured in %.

Subs.	EDG	RgPAL	AvPAL	PbPAL	DdPAL	RsTAL	AL-11
**5a**	2-MeO	15	2	27	10	<1	68
**5b**	3-MeO	35	21	92	45	59	61
**5c**	4-MeO	<1	<1	8	<1	2	5
**5d**	3-MeO-4-F	19	3	79	53	n.d.	91
**5e**	3-MeO-4-Cl	5	13	91	80	2	59
**5f**	3-F-4-MeO	<1	<1	40	<1	22	81
**5g**	3-Cl-4-MeO	2	2	38	2	26	87
**5h**	2-Et	84	18	48	28	n.d.	91
**5i**	3-Et	78	<1	30	<1	8	76
**5j**	4-Et	<1	<1	48	<1	3	5
**5k**	2,3-(OCH_2O)	96	87	92	90	n.d.	98
**5l**	3,4-(OCH_2O)	<1	<1	3	<1	2	11
**5m**	2,3-$(MeO)_2$	<1	<1	<1	<1	14	5
**5n**	2,4-$(MeO)_2$	<1	<1	<1	<1	<1	<1
**5o**	3,4-$(MeO)_2$	<1	<1	<1	<1	<1	92
**5p**	3,4-$(MeO)_2$-6-F	<1	<1	<1	<1	6	96
**5q**	3,5-$(MeO)_2$	<1	<1	<1	<1	26	98
**5r**	2,4,6-$(MeO)_3$	<1	<1	<1	<1	<1	<1
**5s**	3,4,5-$(MeO)_3$	<1	<1	<1	<1	n.d.	97
**5t**	2,3,4-$(MeO)_3$	NT	NT	NT	NT	<1	4
**5u**	2,4,5-$(MeO)_3$	NT	NT	NT	NT	<1	73

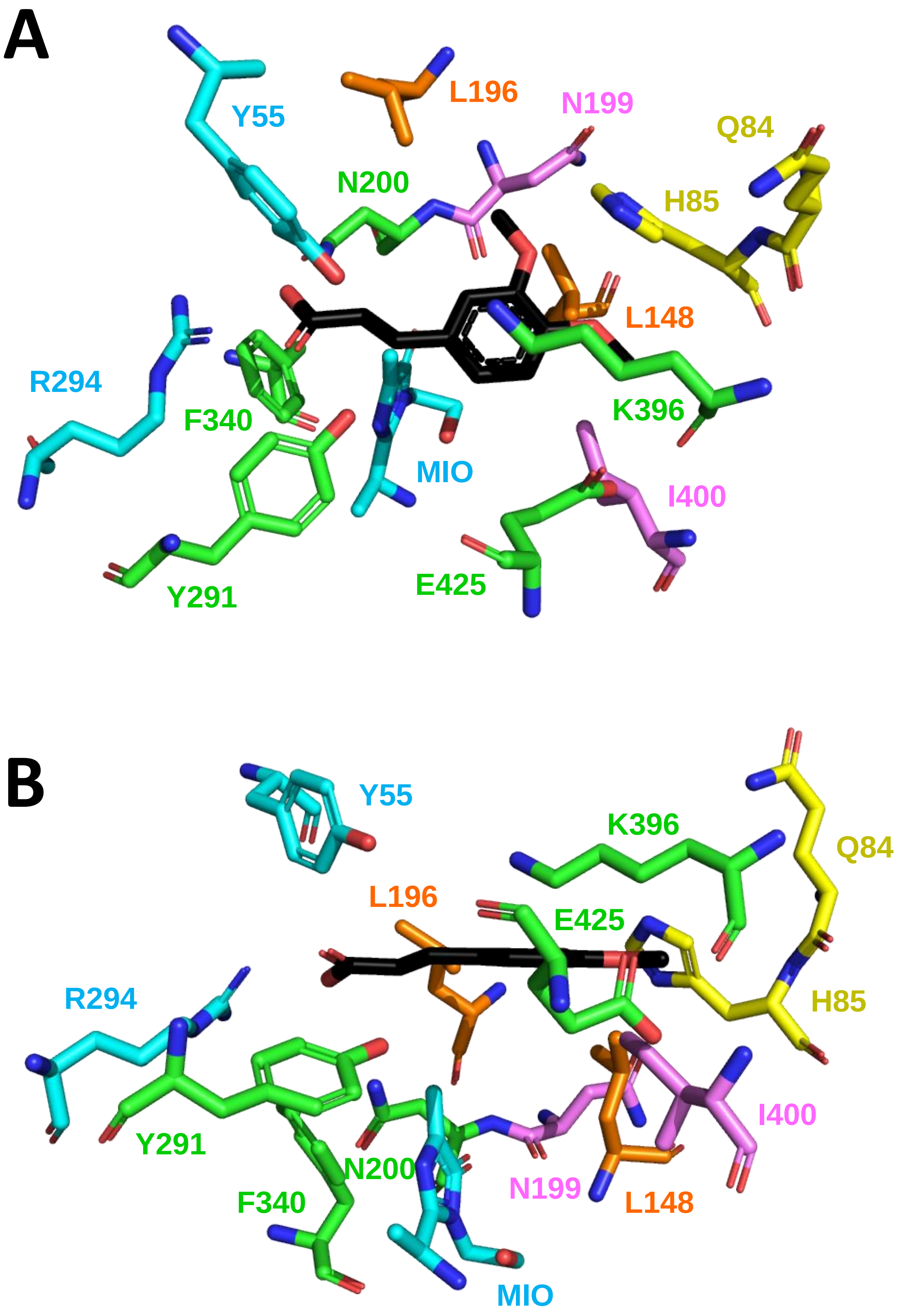

Figure S11: Homology model of the active site of AL-11 enzyme with ligand ***5o*** *docked. Mechanism-related residues are shown in cyan, selectivity residues are shown in yellow, library A residues in orange, library B residues in pink, other residues in close contact in green, substrate in black. (A) Top view. (B) Side view.*

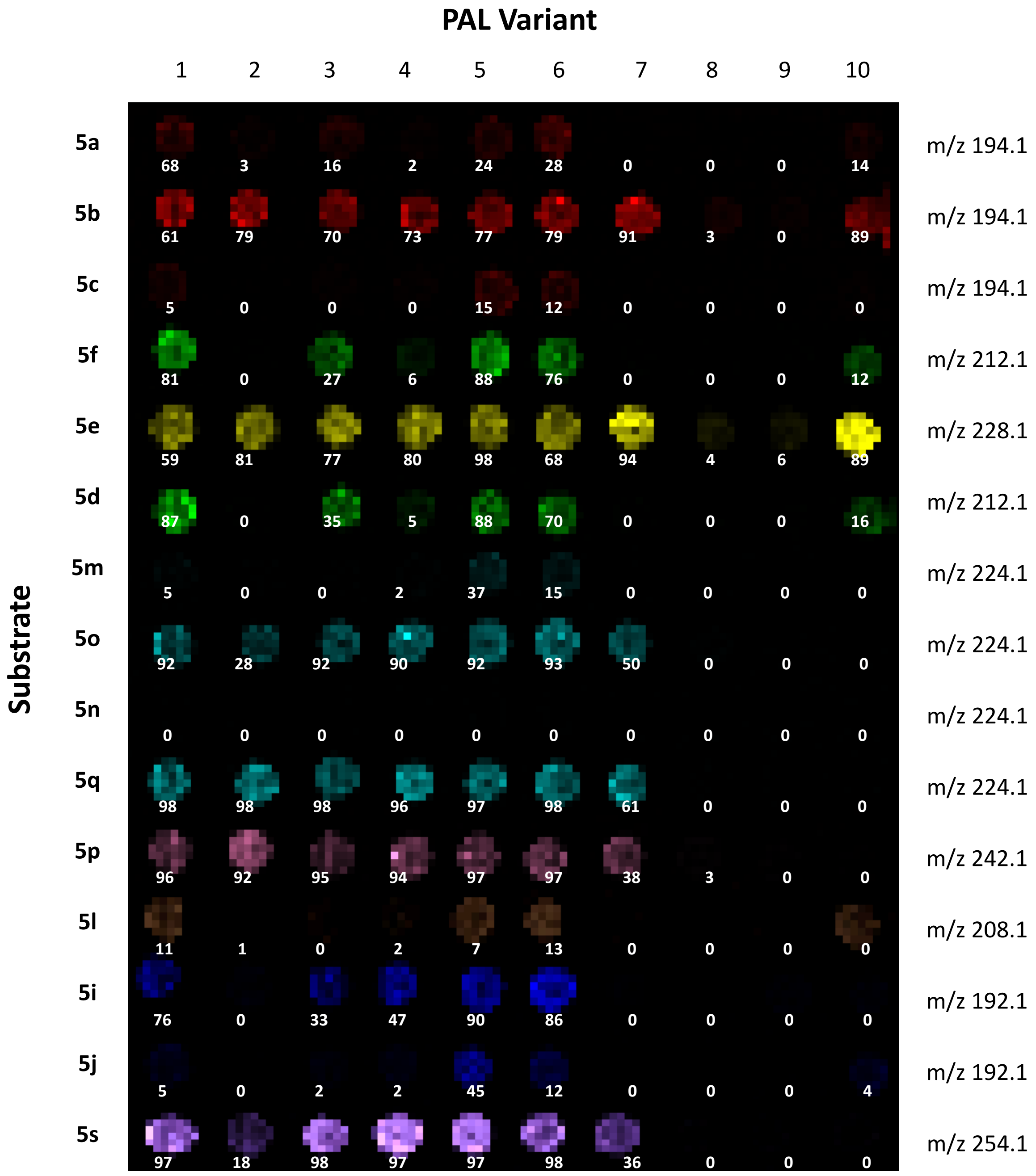

Figure S12: DESI-MS heat map of screening results from PAL whole cell reactions ((AL-11 and 9 mutants) x 15 substrates). The m/z heat maps for each product ion have been assigned differing colours and then collated to obtain a final image indicating locations of product yielding reactions. Reaction conversion data (%) obtained by HPLC-(UV) analysis or each reaction mixture has been overlain (white text) with each reaction location for comparison with DESI-MS results. DESI-MS pixel sizes were set to 500 µm x 500 µm and analysed using a stage speed of 1500 µm/s. This is equivalent to a throughout of 44 s/sample. Variants: 1 = AL-11 wt (Q84-H85); 2 = Q84F, 3 = Q84H, 4 = Q84I; 5: Q84V; 6: Q84A; 7: Q84Y; 8: Q84Y-H85I; 9: Q84I-H85V; 10: Q84A-H85V.

Site-directed mutagenesis for generating AL-11 single and double mutant libraries

Site directed mutagenesis libraries were introduced into the AL-11 gene cloned in the pET28b expression vector. Synthetic oligonucleotide primers were purchased from MWG Eurofins (Table S5) and designed to contain the desired mutations annealed to the plasmid DNA. Site directed mutagenesis procedure involved amplifying the template DNA using an inverse PCR technique with Q5® High-fidelity 2X DNA polymerase master mix from New England Biolabs (Ipswich, MA, USA). The following PCR protocol was used: 1 min denaturation at 98 °C and then 30 cycles of 30 s denaturation at 98 °C, 30 s annealing at 58 °C and 2 min 30 s elongation at 72 °C with a 5 min final extension time at 72 °C. The amplicons were treated with a Dpn1 digest for 1 h at 37 °C and subsequently purified. The purified amplicons (180 ng) were placed in a one-pot phosphorylation and ligation reaction mixture with T4 polynucleotide kinase (10 U) and T4 ligase (20 U) in the presence of ligase buffer containing ATP and left to incubate at room temperature for 1 h. The variants were transformed in *E. coli* DH5α cells and plated onto kanamycin agar plates (50 µg/mL) and left to incubate overnight at 37 °C. Colonies were picked and grown in LB media and the plasmids were subsequently isolated and sequenced to confirm desired mutations. Libraries L148RBT/N196RBT and N199RBT/I400RBT were created using the QuikChange protocol. Inverse PCR was used to create library L196RBT/N199RBT and was phosphorylated and ligated and then transformed in DH5α cells as above.

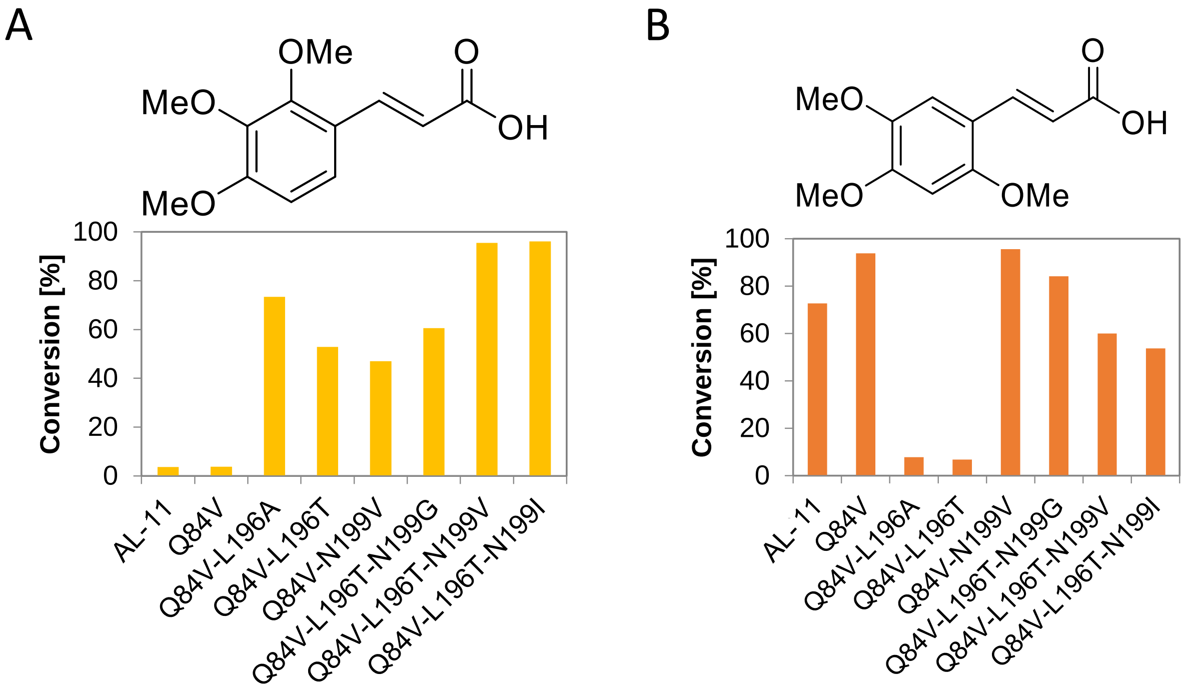

Figure S13: Conversion values (LC-MS) of additional analytical scale hydroamination reactions with AL-11 and newly identified mutants with trisubsituted cinnamic acid substrates. A) Substrate ***5t****. B) Substrate* ***5u****.*

Supporting Information References

[S1] Keenan, T. *et al*. Profiling Substrate Promiscuity of Wild-Type Sugar Kinases for Multi-fluorinated Monosaccharides. *Cell Chem. Biol.* 1–8 (2020). doi:10.1016/j.chembiol.2020.06.005.

[S2] Aleku, G. *et al*. A reductive aminase from *Aspergillus oryzae*. *Nature Chem* **9**, 961–969 (2017). doi:10.1038/nchem.2782
