# Supplementary material for: Rapid Screening of Diverse Biotransformations for Enzyme Evolution
Source: JACS Au. 2021 Apr 8;1(4):508–16. doi: 10.1021/jacsau.1c00027 (PMC8154213; doi:10.1021/jacsau.1c00027)
Supplement: Supplementary file 2 — au1c00027_si_002.pdf [file au1c00027_si_002.pdf]

Supplementary dataset S1
Compound characterisation data for arylacrylic acid substrates

Cinnamic acid derivatives **5a-5p** have been prepared and characterised previously.[S1]

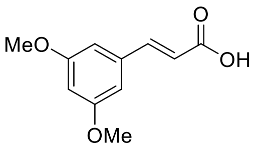

(*E*)-3-(3,5-dimethoxyphenyl)acrylic acid (5q)
White solid, 94% isolated yield.
^{1}H NMR (400 MHz, DMSO-d_6): δ 7.51 (d, *J* = 16.0, 1H), 6.86 (d, *J* = 1.8, 2H), 6.55 (d, *J* = 16.0, 1H), 6.53 (s, 1H), 3.77 (s, 6H).
^{13}C NMR (101 MHz, DMSO-d_6): δ 167.6, 160.7, 144.0, 136.2, 119.9, 106.1, 102.4, 55.4.

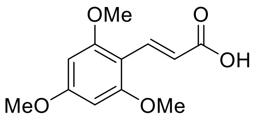

(*E*)-3-(2,4,6-trimethoxyphenyl)acrylic acid (5r)
White solid, 96% isolated yield.
^{1}H NMR (400 MHz, DMSO-d_6): δ 7.88 (d, *J* = 16.1, 1H), 6.56 (d, *J* = 16.1, 1H), 6.28 (s, 2H), 3.85 (s, 6H), 3.83 (s, 3H).
^{13}C NMR (101 MHz, DMSO-d_6): δ 169.2, 162.8, 160.8, 134.8, 117.5, 104.4, 90.9, 55.6, 55.5.

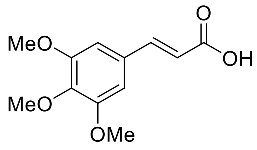

(*E*)-3-(3,4,5-trimethoxyphenyl)acrylic acid (5s)
White solid, 91% isolated yield.
^{1}H NMR (400 MHz, $CDCl_3$): δ 7.71 (d, *J* = 15.8, 1H), 6.78 (s, 2H), 6.36 (d, *J* = 15.8, 1H), 3.89 (s, 6H), 3.88 (s, 3H).
^{13}C NMR (101 MHz, $CDCl_3$): δ 172.6, 153.6, 147.2, 140.6, 129.6, 116.6, 105.6, 61.1, 56.3.

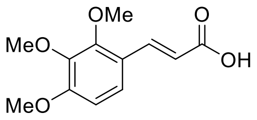

(*E*)-3-(2,3,4-trimethoxyphenyl)acrylic acid (5t)
White solid, 88% isolated yield.
^{1}H NMR (500 MHz, DMSO-d_6): δ 7.70 (d, *J* = 16.1, 1H), 7.48 (d, *J* = 8.9, 1H), 6.87 (d, *J* = 8.9, 1H), 6.43 (d, *J* = 16.1, 1H), 3.84 (s, 3H), 3.83 (s, 3H), 3.76 (s, 3H).
^{13}C NMR (125 MHz, DMSO-d_6): δ 168.4, 155.7, 152.9, 142.2, 138.8, 123.5, 120.9, 118.1, 108.9, 61.8, 60.9, 56.5.

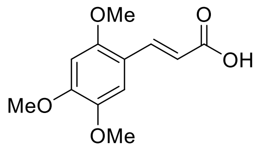

(*E*)-3-(2,4,5-trimethoxyphenyl)acrylic acid (5u)
Yellow solid, 95% isolated yield.
^{1}H NMR (500 MHz, DMSO-d_6): δ 7.81 (d, *J* = 16.0, 1H), 7.24 (s, 1H), 6.72 (s, 1H), 6.42 (d, *J* = 16.0, 1H), 3.86 (s, 3H), 3.85 (s, 3H), 3.76 (s, 3H).
^{13}C NMR (125 MHz, DMSO-d_6): δ 168.7,153.7, 152.6, 143.5, 138.9, 116.5, 114.2, 111.4, 56.8, 56.6, 56.2.

Copies of NMR spectra

(*E*)-3-(3,5-dimethoxyphenyl)acrylic acid (5q)
FP1267

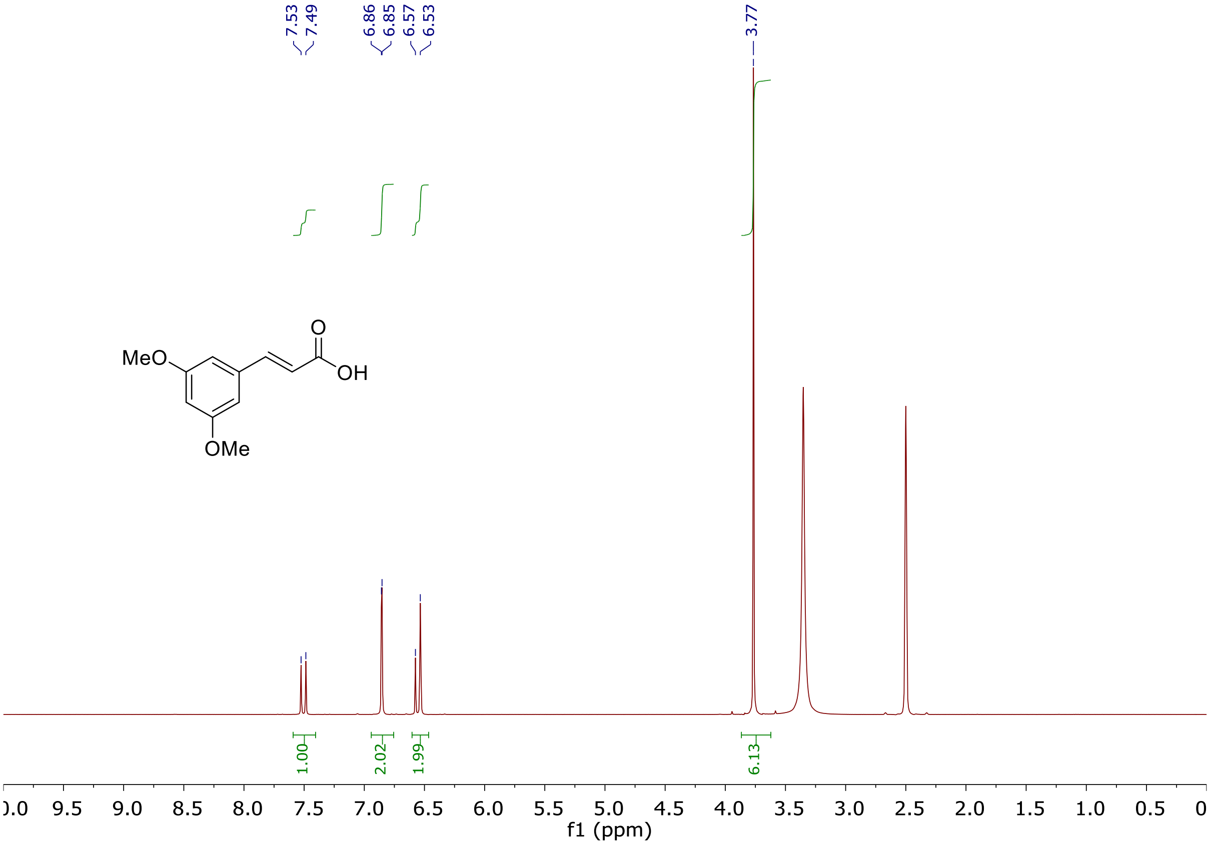

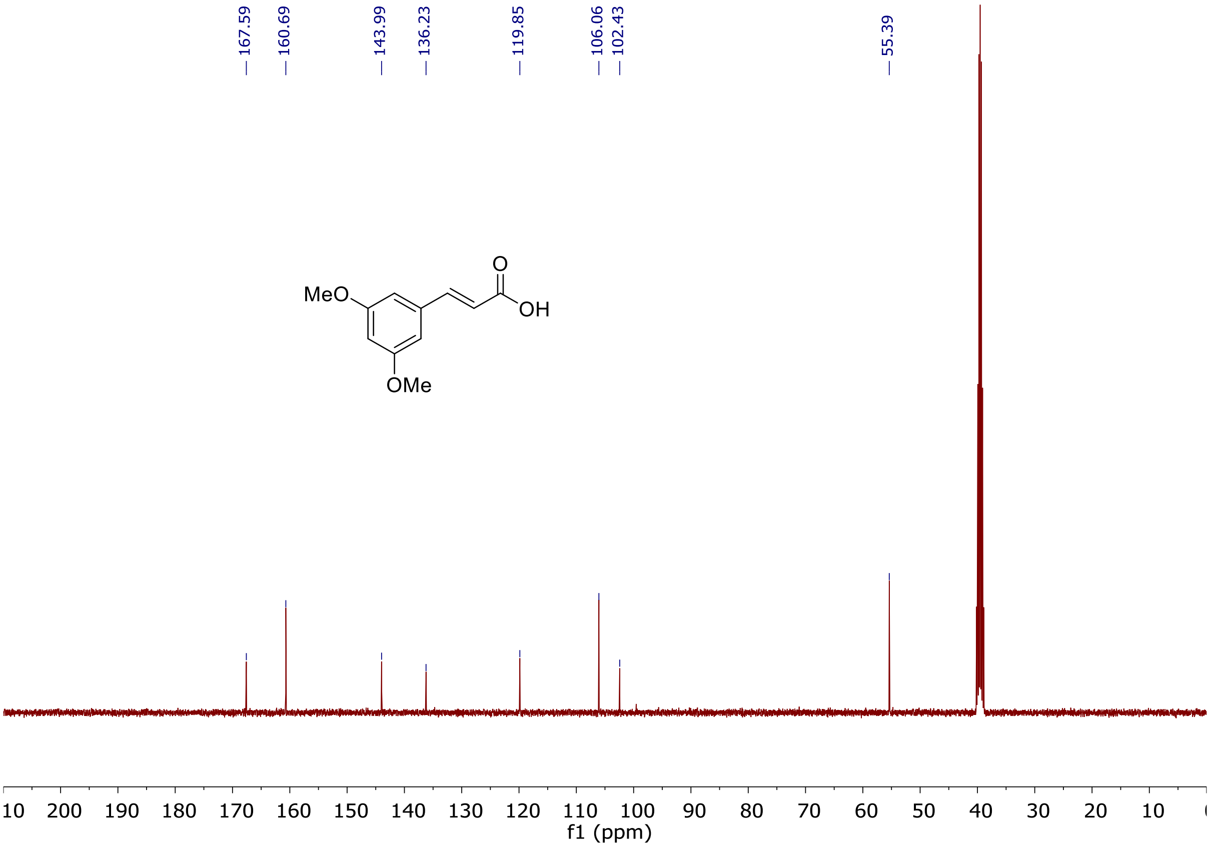

(*E*)-3-(2,4,6-trimethoxyphenyl)acrylic acid (5r)

FP1528

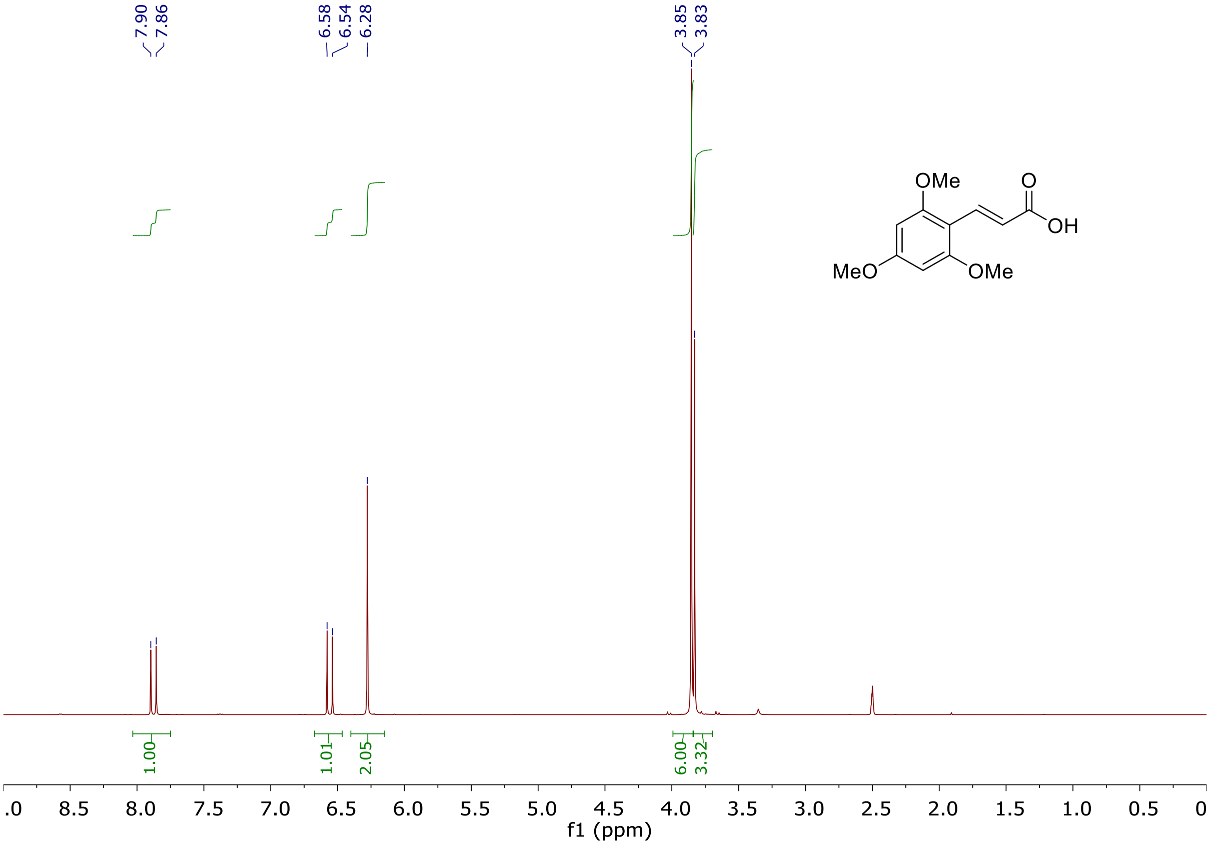

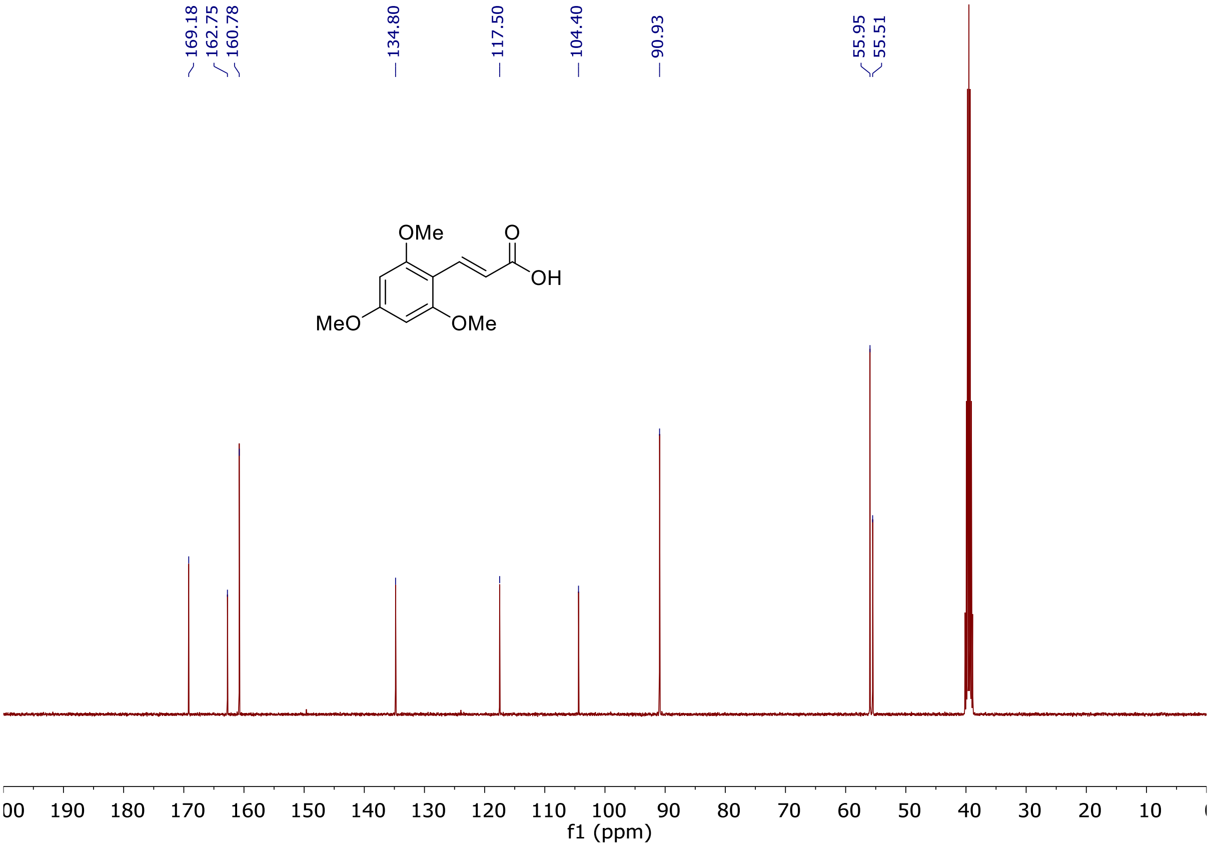

(*E*)-3-(3,4,5-trimethoxyphenyl)acrylic acid (5*s*)

FP1527

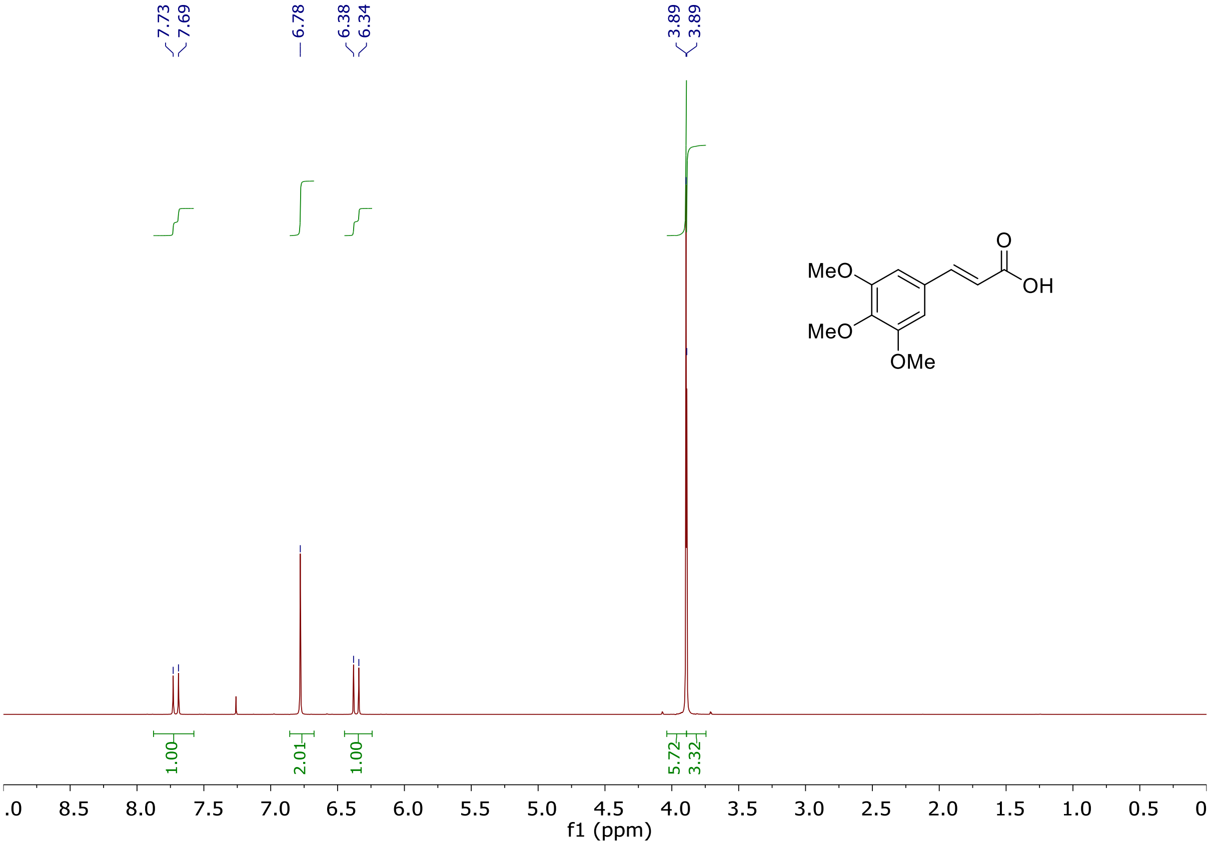

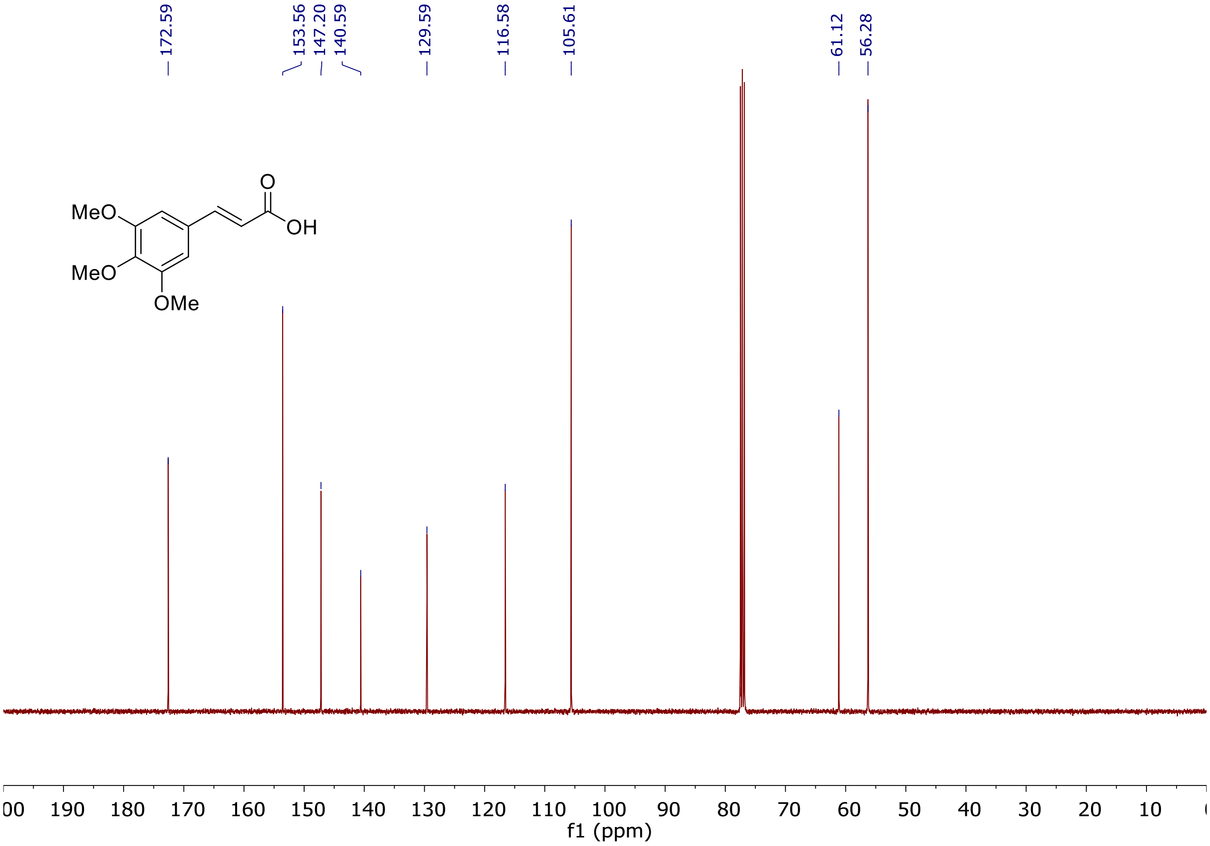

(*E*)-3-(2,3,4-trimethoxyphenyl)acrylic acid (5t)

FP1632

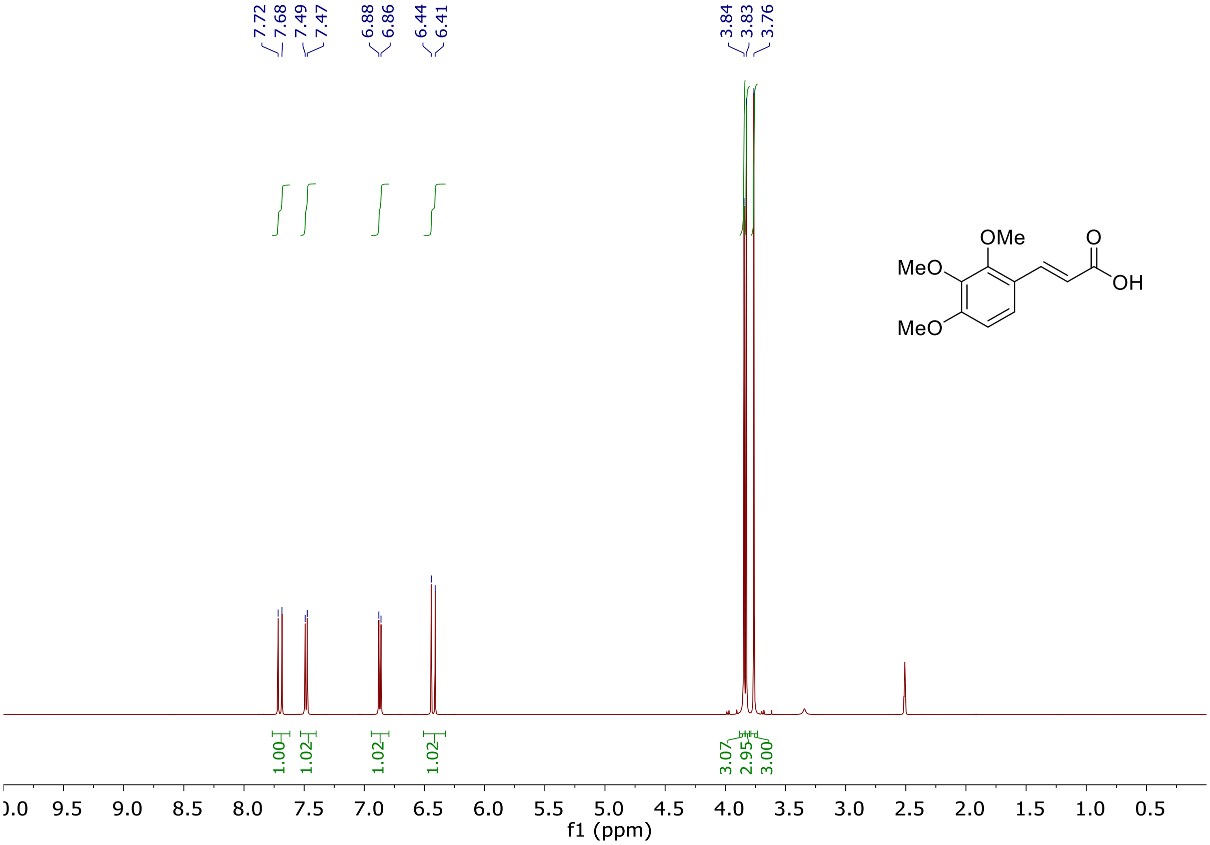

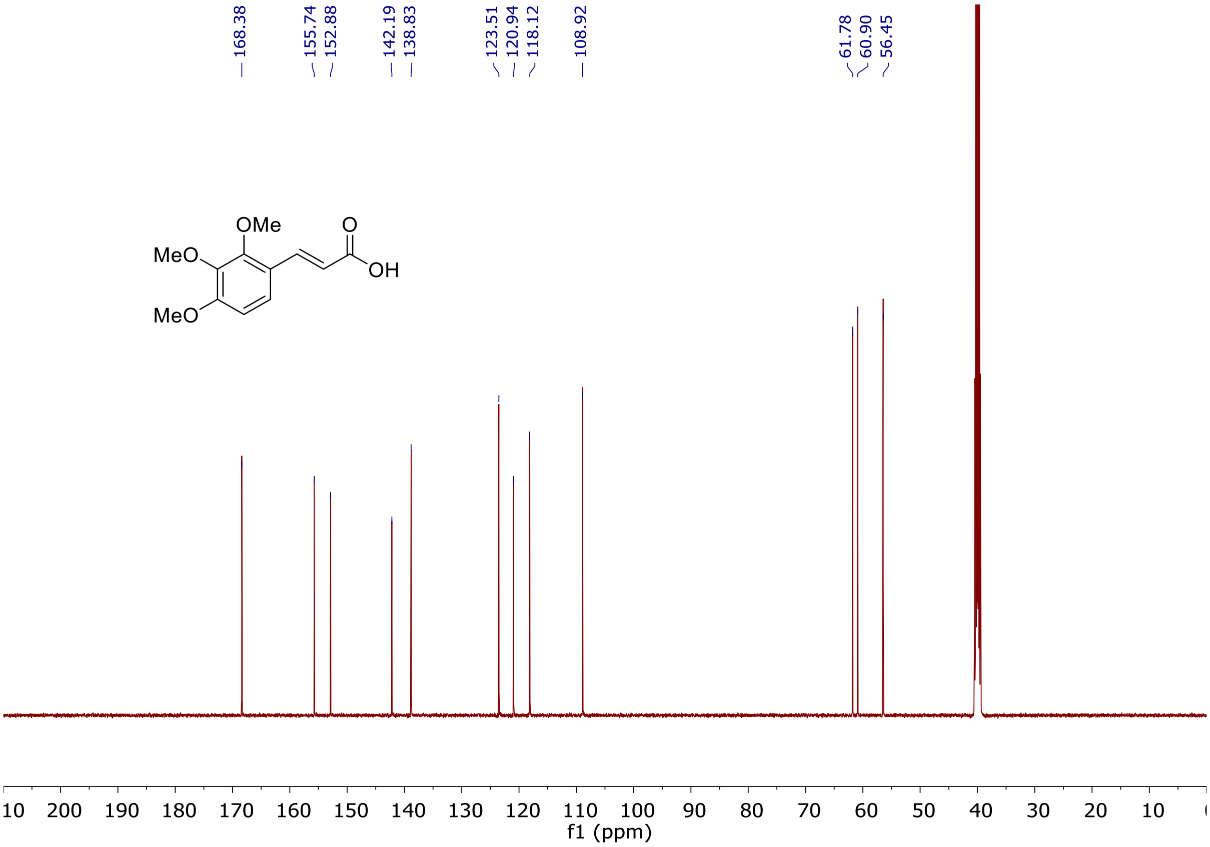

(*E*)-3-(2,4,5-trimethoxyphenyl)acrylic acid (5u)

FP1638

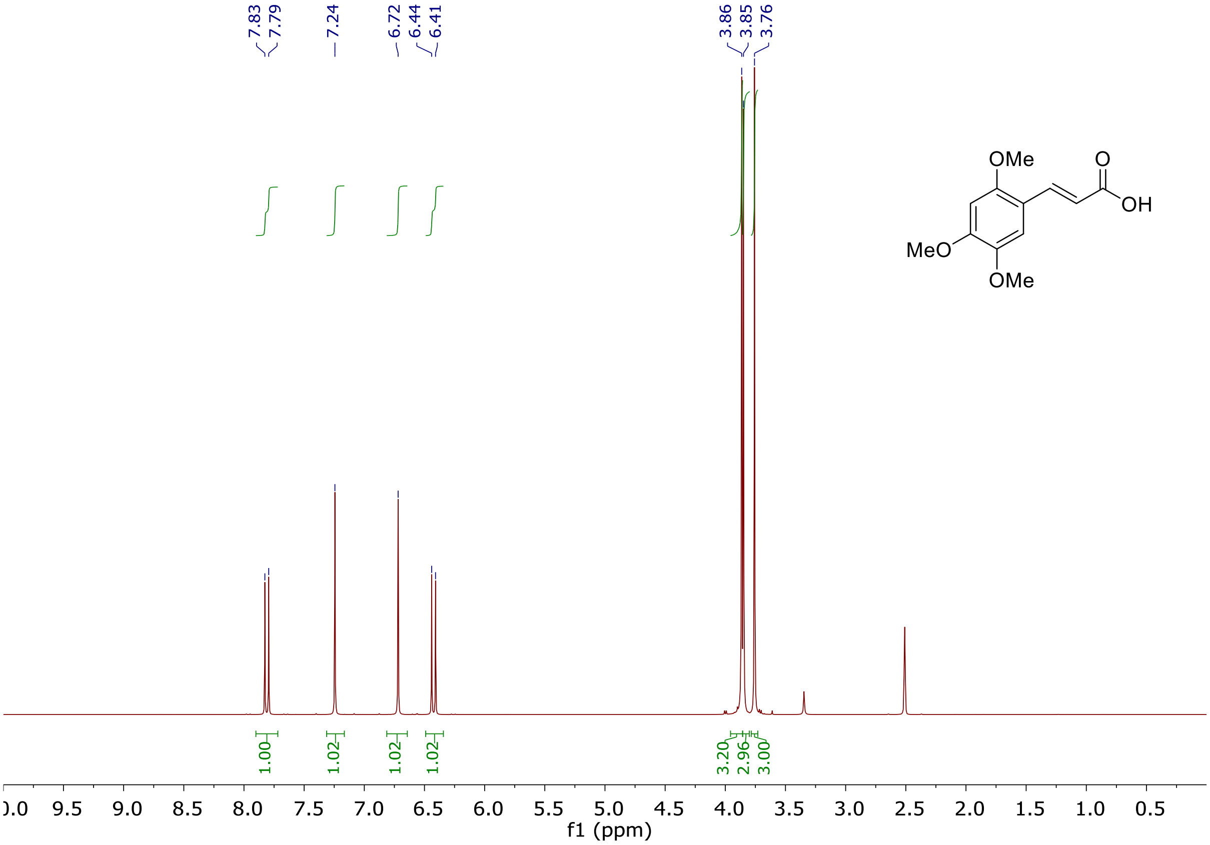

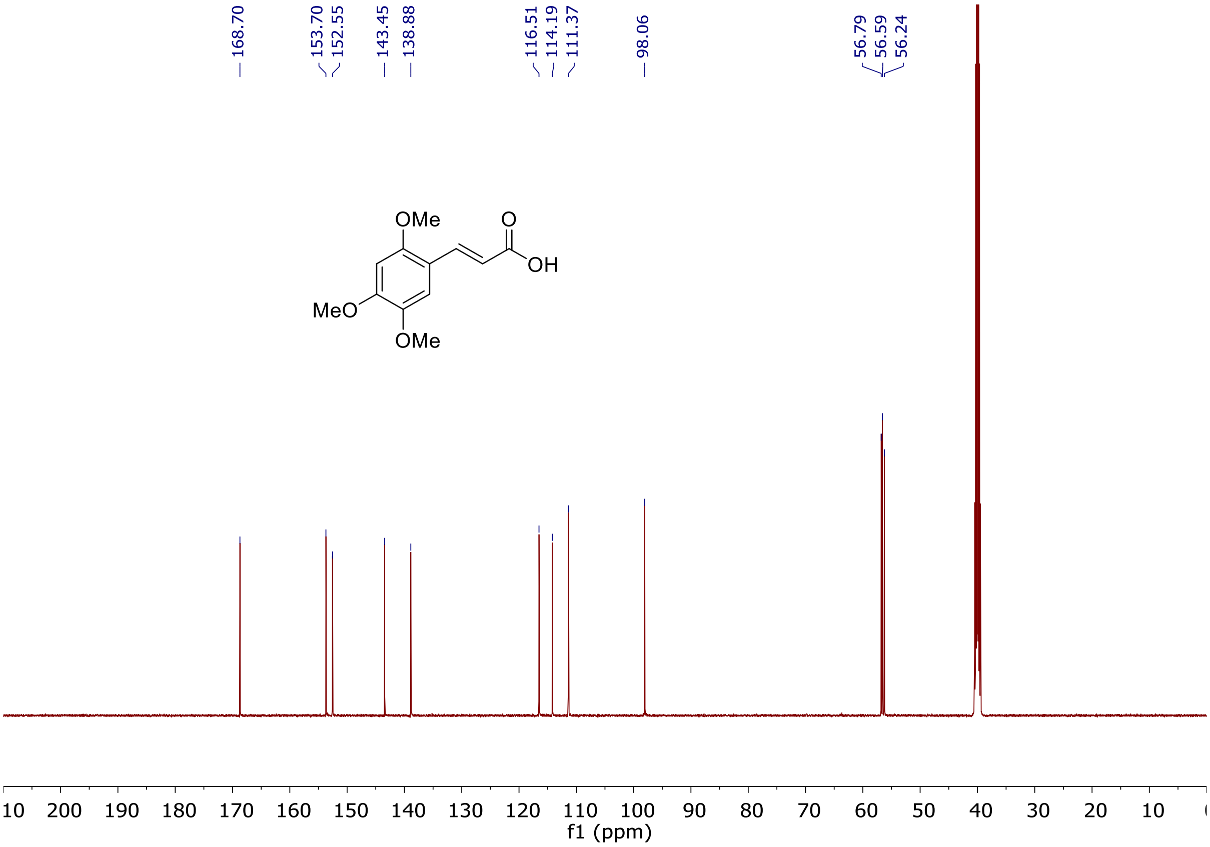

References

[S1] Ahmed S. T., Parmeggiani F., Weise N. J., Flitsch S. L., Turner N. J., Engineered Ammonia Lyases for the Production of Challenging Electron-Rich L-Phenylalanines. *ACS Catal.* **2018**, *8*, 3129-3132.
