# Supplementary material for: Rapid Screening of Diverse Biotransformations for Enzyme Evolution
Source: JACS Au. 2021 Apr 8;1(4):508–16. doi: 10.1021/jacsau.1c00027 (PMC8154213; doi:10.1021/jacsau.1c00027)
Supplement: Supplementary file 3 — au1c00027_si_003.pdf [file au1c00027_si_003.pdf]

Supplementary dataset S2
Compound characterisation data for isolated amino acid products

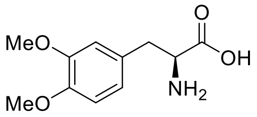

(*S*)-2-amino-3-(3,4-dimethoxyphenyl)propanoic acid (6o)
White solid, 81% isolated yield.
^{1}H NMR (400 MHz, D_2O+NaOH): δ 6.91 (d, *J* = 8.2, 1H), 6.84 (d, *J* = 1.8, 1H), 6.77 (dd, *J* = 8.2, 1.8, 1H), 3.78 (s, 3H), 3.76 (s, 3H), 3.40 (dd$_X$ of ABX, *J* = 7.1, 5.6, 1H), 2.86 (dd$_A$ of ABX, *J* = 13.6, 5.6, 1H), 2.73 (dd$_B$ of ABX, *J* = 13.6, 7.1, 1H).
^{13}C NMR (101 MHz, D_2O+NaOH): δ 182.4, 147.8, 146.6, 131.3, 121.9, 112.9, 111.8, 57.3, 55.6, 55.5, 40.2.
HRMS (ESI): *m/z* [M+H]$^+$ $C_{11}H_{16}NO_4^+$ calcd. 226.1074, found 226.1084.

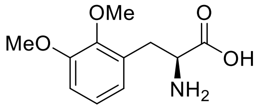

(*S*)-2-amino-3-(2,3-dimethoxyphenyl)propanoic acid (6m)
White solid, 75% isolated yield.
^{1}H NMR (400 MHz, D_2O+NaOH): δ 7.05 (dd, *J* = 8.3, 7.7, 1H), 6.93 (dd, *J* = 8.3, 1.3, 1H), 6.82 (dd, *J* = 7.7, 1.3, 1H), 3.80 (s, 3H), 3.72 (s, 3H), 3.41 (dd$_X$ of ABX, *J* = 8.4, 5.8, 1H), 2.95 (dd$_A$ of ABX, *J* = 13.5, 5.8, 1H), 2.72 (dd$_B$ of ABX, *J* = 13.5, 8.4, 1H).
^{13}C NMR (101 MHz, D_2O+NaOH): δ 182.5, 152.2, 146.6, 132.3, 124.8, 123.0, 111.6, 68.8, 57.1, 55.7, 34.9.
HRMS (ESI): *m/z* [M+H]$^+$ $C_{11}H_{16}NO_4^+$ calcd. 226.1074, found 226.1075.

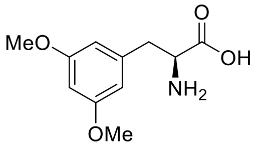

(*S*)-2-amino-3-(3,5-dimethoxyphenyl)propanoic acid (6q)
White solid, 79% isolated yield.
^{1}H NMR (400 MHz, D_2O+NaOH): δ 6.45 (d, *J* = 2.3, 2H), 6.39 (t, *J* = 2.3, 1H), 3.74 (s, 6H), 3.42 (dd$_X$ of ABX, *J* = 7.4, 5.5, 1H), 2.88 (dd$_A$ of ABX, *J* = 13.5, 5.5, 1H), 2.71 (dd$_B$ of ABX, *J* = 13.5, 7.4, 1H).
^{13}C NMR (101 MHz, D_2O+NaOH): δ 182.2, 160.0, 141.0, 107.7, 98.6, 57.2, 55.3, 40.9.
HRMS (ESI): *m/z* [M+H]$^+$ $C_{11}H_{16}NO_4^+$ calcd. 226.1074, found 226.1090.

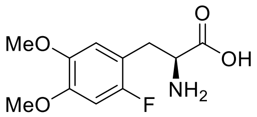

(*S*)-2-amino-3-(2-fluoro-4,5-dimethoxyphenyl)propanoic acid (6p)
White solid, 90% isolated yield.
^{1}H NMR (400 MHz, D_2O+NaOH): δ 6.71-6.80 (m, 2H), 3.75 (s, 3H+3H), 3.40 (dd$_X$ of ABX, *J* = 7.3, 6.0, 1H), 2.86 (ddd$_A$ of ABX, *J* = 13.7, 6.0, J_{CF} = 1.0, 1H), 2.73 (ddd$_B$ of ABX, *J* = 13.7, 7.3, J_{CF} = 1.0, 1H).
^{13}C NMR (101 MHz, D_2O+NaOH): δ 182.2, 155.4 (d, J_{CF} = 237.1), 147.4 (d, J_{CF} = 10.3), 143.9 (d, J_{CF} = 2.6), 115.8 (d, J_{CF} = 17.8), 113.9 (d, J_{CF} = 6.3), 100.2 (d, J_{CF} = 29.1), 56.7, 56.1, 55.8, 33.7.
HRMS (ESI): *m/z* [M+H]$^+$ $C_{11}H_{15}FNO_4^+$ calcd. 244.0980, found 244.0984.

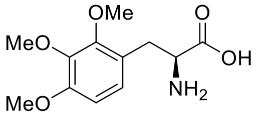

(*S*)-2-amino-3-(2,3,4-trimethoxyphenyl)propanoic acid (6t)
White solid, 88% isolated yield.
^{1}H NMR (400 MHz, D_2O+NaOH): δ 6.92 (d, *J* = 8.6, 1H), 6.77 (d, *J* = 8.6, 1H), 3.80 (s, 3H), 3.79 (s, 3H+3H), 3.37 (dd$_X$ of ABX, *J* = 8.2, 5.9, 1H), 2.88 (dd$_A$ of ABX, *J* = 13.7, 5.9, 1H), 2.65 (dd$_B$ of ABX, *J* = 13.7, 8.2, 1H).
^{13}C NMR (101 MHz, D_2O+NaOH): δ 182.5, 151.7, 151.3, 141.2, 125.8, 124.6, 108.5, 61.3, 60.9, 57.1, 56.0, 34.8.
HRMS (ESI): *m/z* [M+H]$^+$ $C_{12}H_{18}NO_5^+$ calcd. 256.1179, found 256.1180.

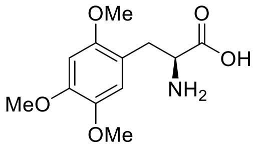

(*S*)-2-amino-3-(2,4,5-trimethoxyphenyl)propanoic acid (6u)
White solid, 68% isolated yield.
^{1}H NMR (400 MHz, D_2O+NaOH): δ 6.78 (s, 1H), 6.66 (s, 1H), 3.78 (s, 3H), 3.74 (s, 3H), 3.73 (s, 3H), 3.38 (dd_X of ABX, *J* = 7.8, 5.8, 1H), 2.86 (dd_A of ABX, *J* = 13.5, 5.8, 1H), 2.65 (dd_B of ABX, *J* = 13.5, 7.8, 1H).
^{13}C NMR (101 MHz, D_2O+NaOH): δ 182.6, 152.0, 147.4, 141.9, 118.6, 115.2, 98.7, 56.7, 56.6, 56.4, 55.9, 34.8.
HRMS (ESI): *m/z* $[M+H]^+$ $C_{12}H_{18}NO_5^+$ calcd. 256.1179, found 256.1180.

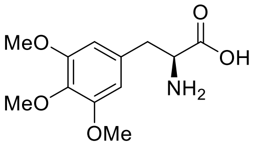

(*S*)-2-amino-3-(3,4,5-trimethoxyphenyl)propanoic acid (6s)
White solid, 74% isolated yield.
^{1}H NMR (400 MHz, D_2O+NaOH): δ 6.51 (s, 2H), 3.77 (s, 6H), 3.66 (s, 3H), 3.42 (dd_X of ABX, *J* = 7.3, 5.5, 1H), 2.86 (dd_A of ABX, *J* = 13.6, 5.5, 1H), 2.71 (dd_B of ABX, *J* = 13.6, 7.3, 1H).
^{13}C NMR (101 MHz, D_2O+NaOH): δ 181.8, 152.1, 135.1, 134.9, 106.6, 60.8, 57.2, 55.9, 40.8.
HRMS (ESI): *m/z* $[M+H]^+$ $C_{12}H_{18}NO_5^+$ calcd. 256.1179, found 256.1187.

Copies of NMR and HRMS spectra

(*S*)-2-amino-3-(2,3-dimethoxyphenyl)propanoic acid (6m)

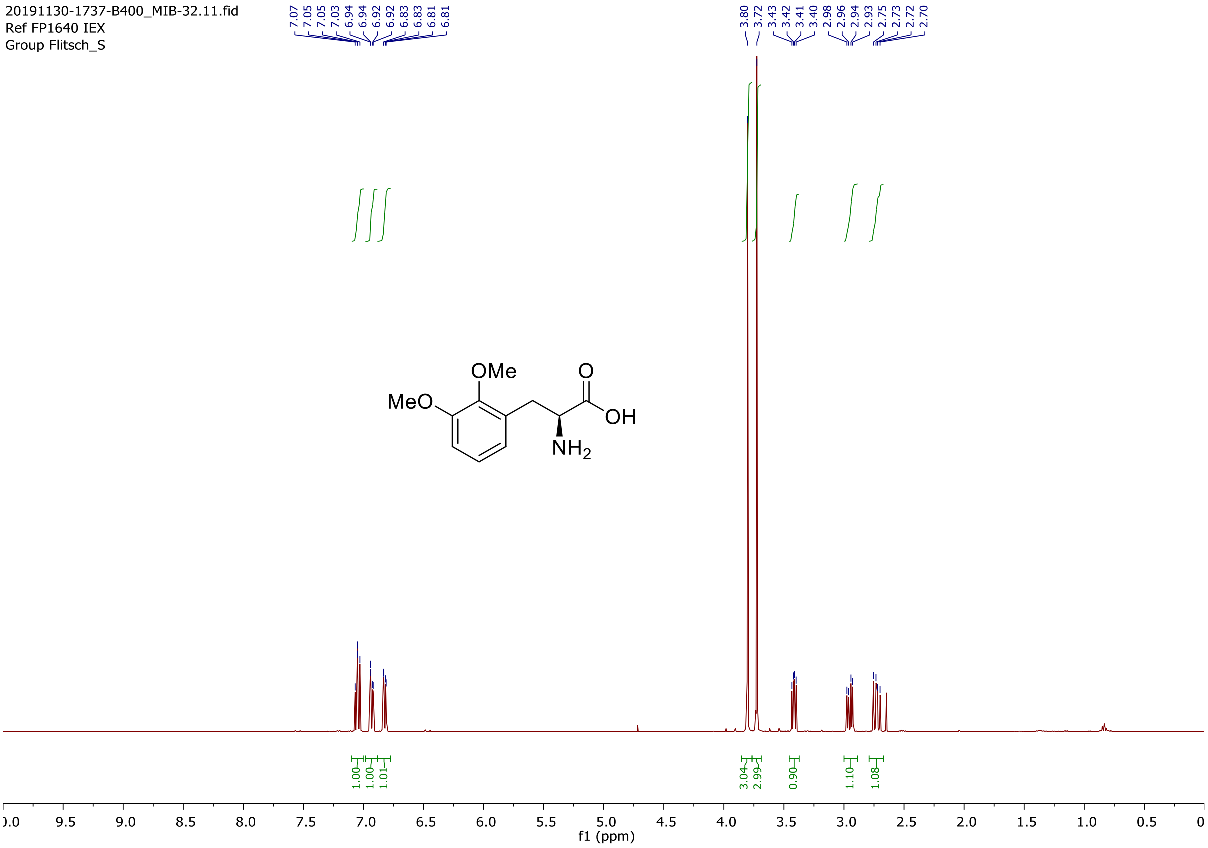

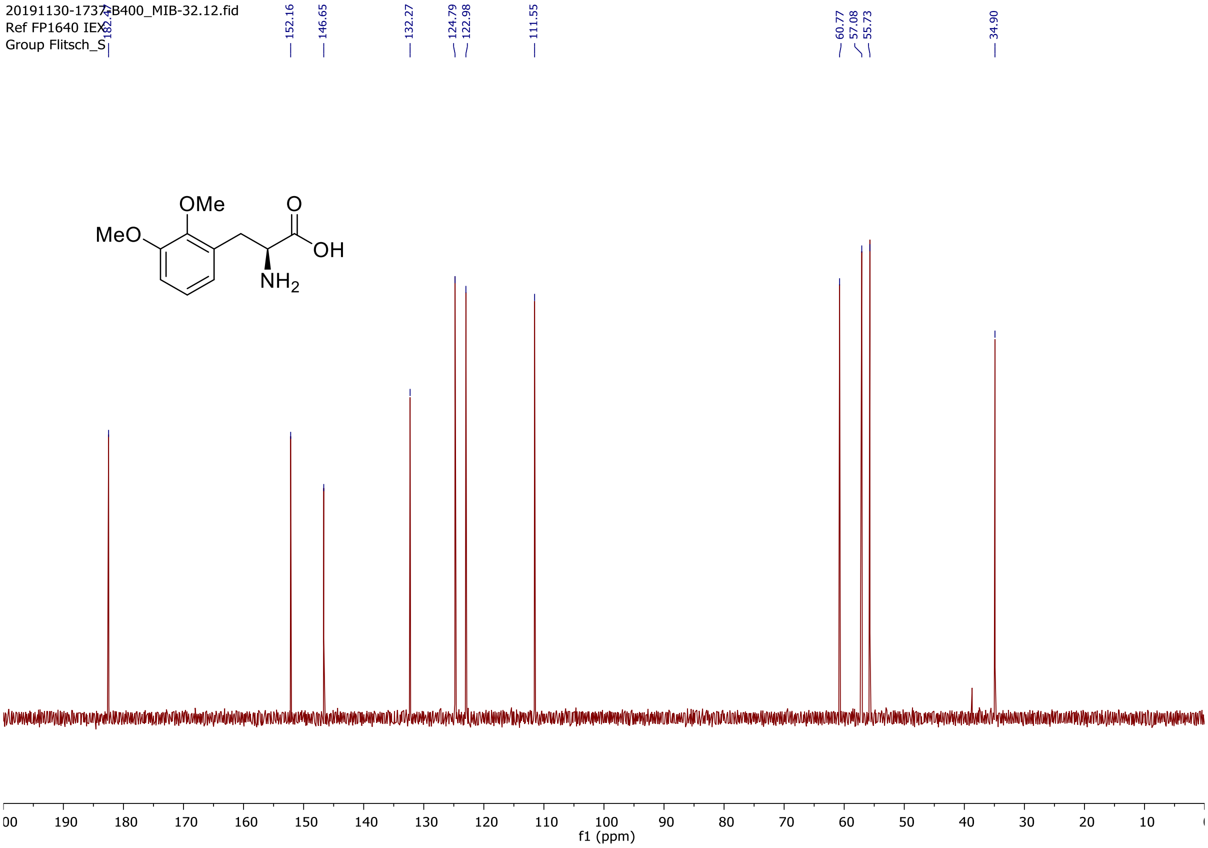

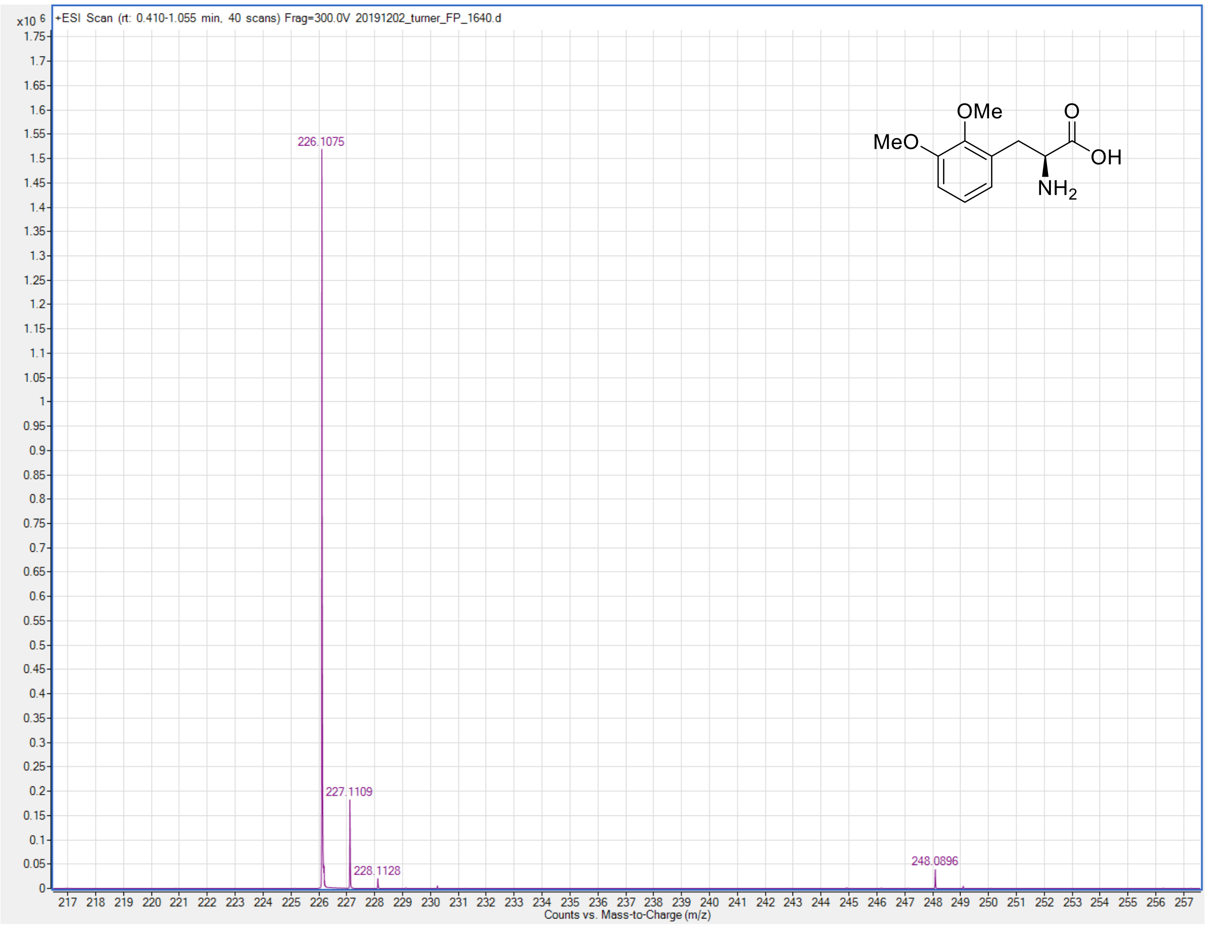

x10 6
+ESI Scan (rt: 0.410-1.055 min, 40 scans) Frag=300.0V 20191202_turner_FP_1640.d
226.1075
227.1109
228.1128
248.0896
Counts vs. Mass-to-Charge (m/z)
OMe
MeO
O
OH
NH2

(*S*)-2-amino-3-(3,4-dimethoxyphenyl)propanoic acid (6o)

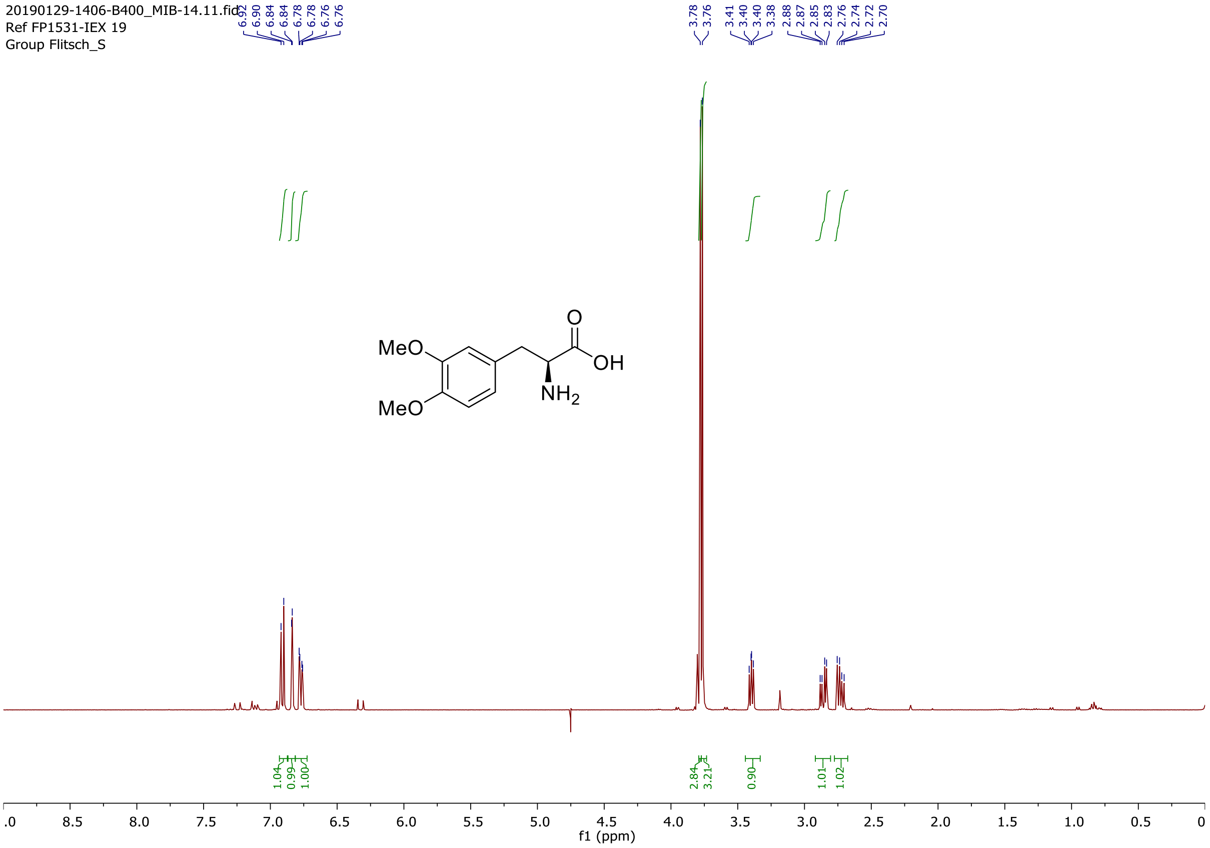

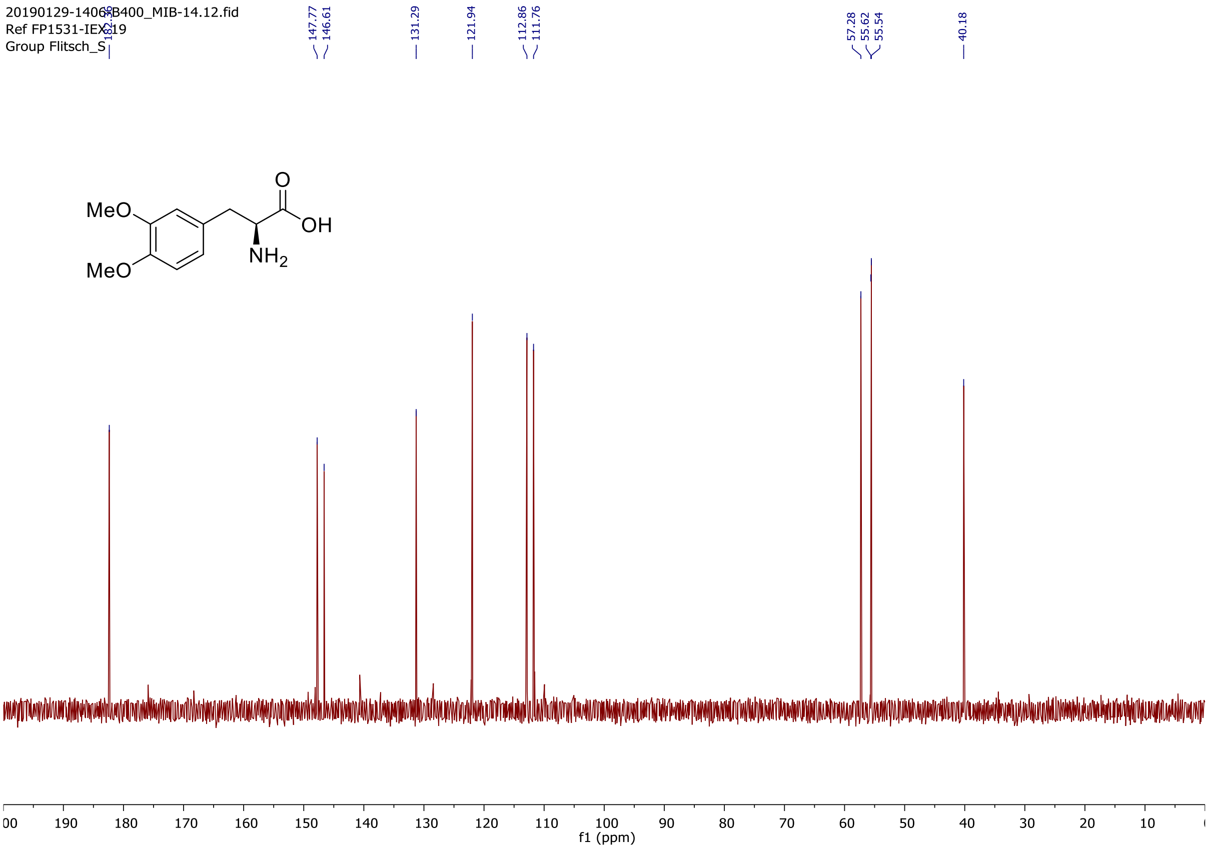

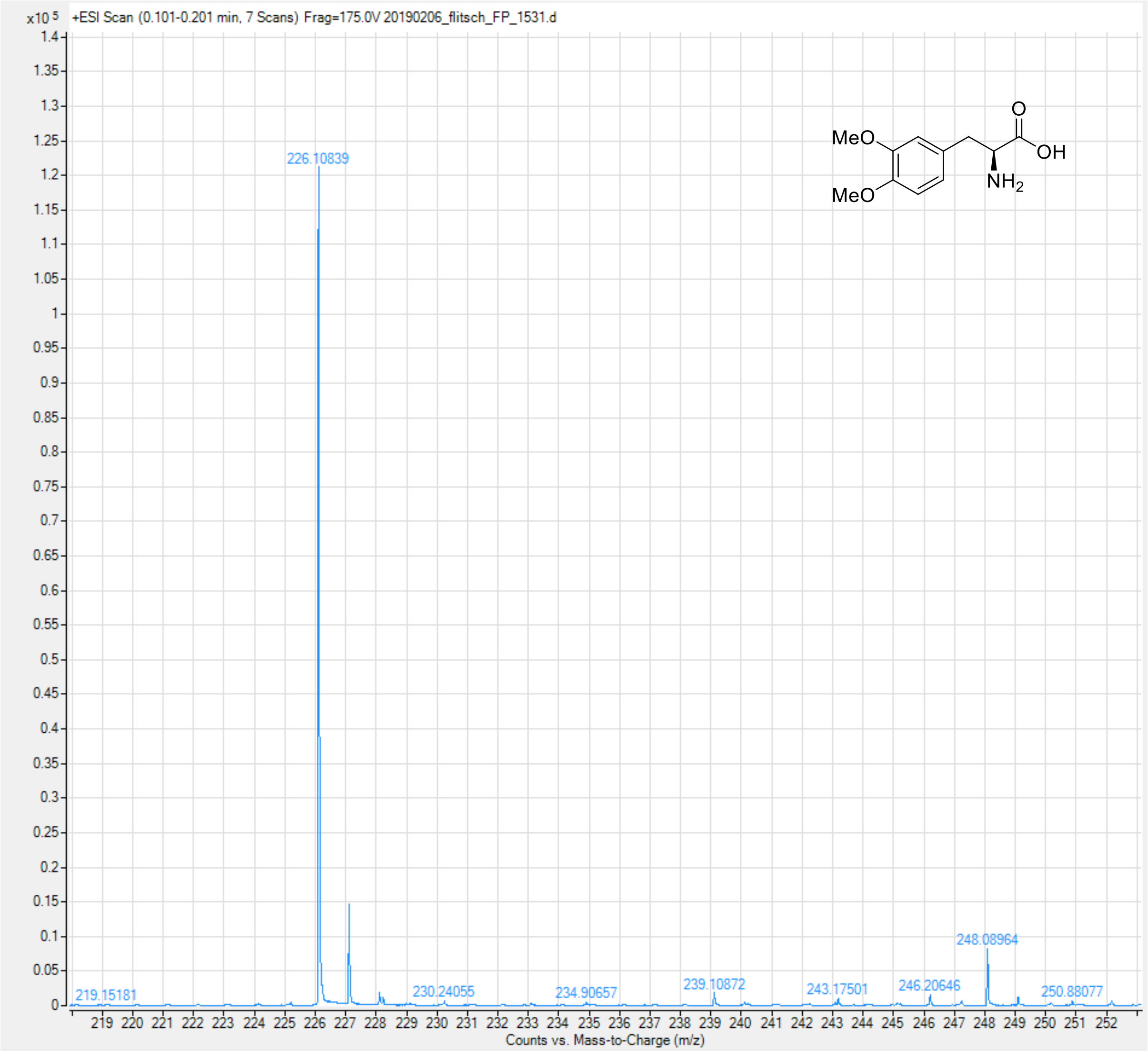
x10 5
+ESI Scan (0.101-0.201 min, 7 Scans) Frag=175.0V 20190206_flitsch_FP_1531.d
226.10839
230.24055
234.90657
239.10872
243.17501
246.20646
248.08964
250.88077
219.15181
MeO
MeO
O
OH
NH2
Counts vs. Mass-to-Charge (m/z)

(*S*)-2-amino-3-(2-fluoro-4,5-dimethoxyphenyl)propanoic acid (6p)

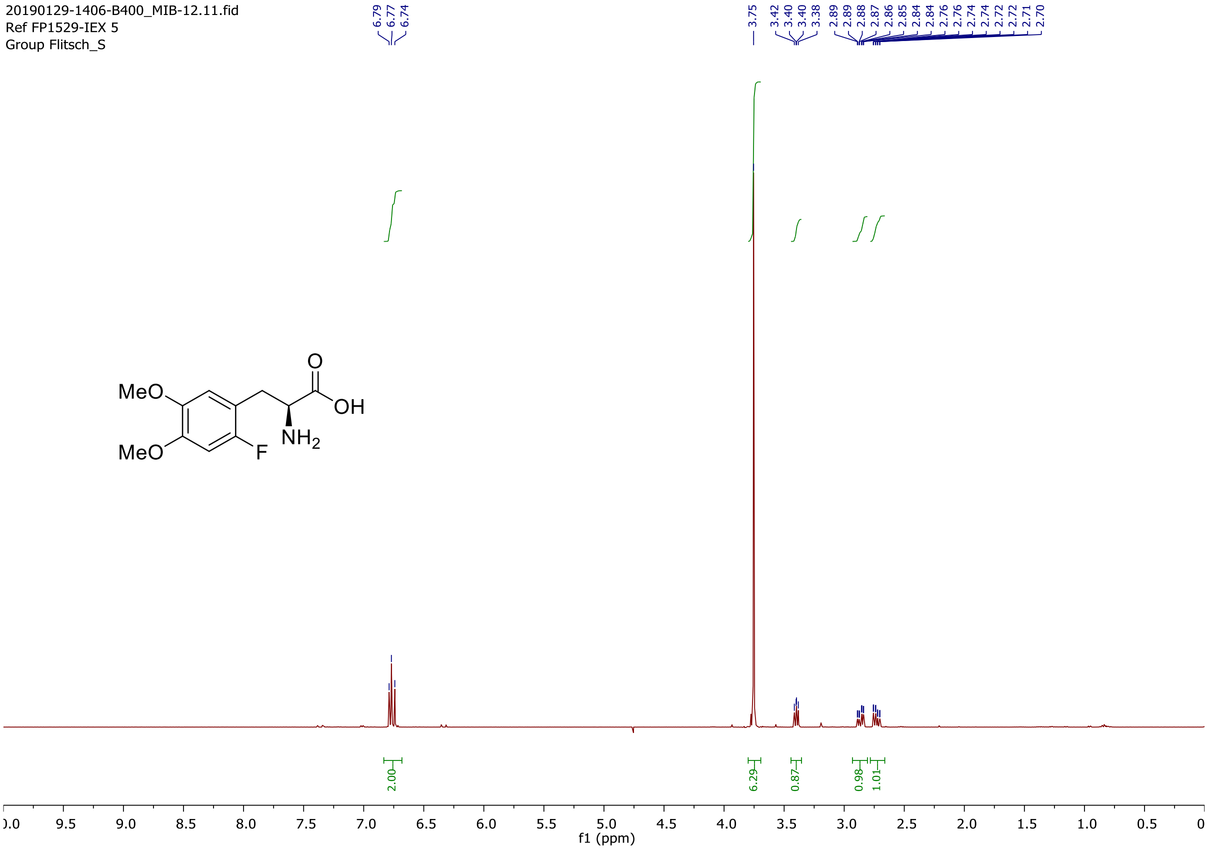

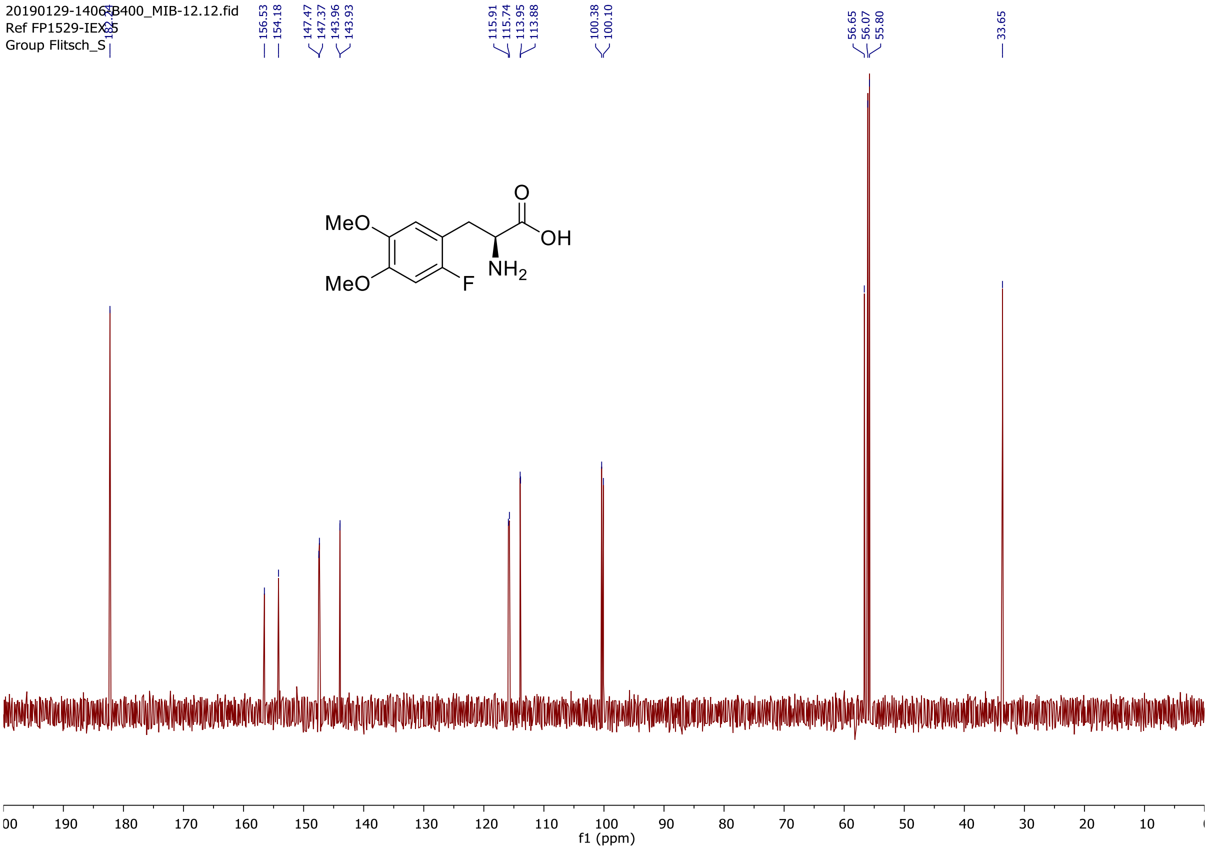

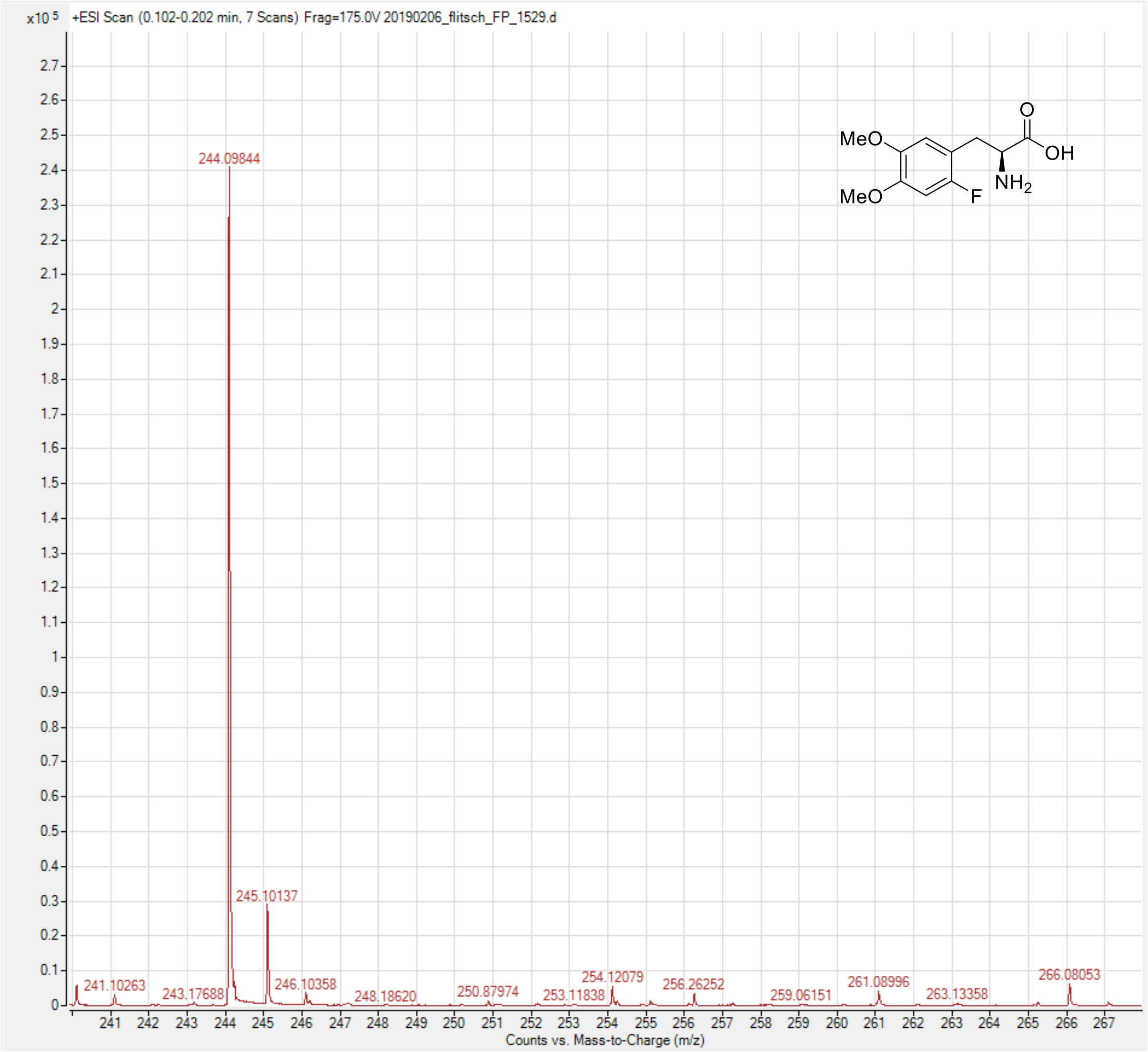
x10 5
+ESI Scan (0.102-0.202 min, 7 Scans) Frag=175.0V 20190206_flitsch_FP_1529.d
244.09844
245.10137
241.10263
243.17688
246.10358
248.18620
250.87974
253.11838
254.12079
256.26252
259.06151
261.08996
263.13358
266.08053
Counts vs. Mass-to-Charge (m/z)
MeO
MeO
F
NH2
OH
O

(*S*)-2-amino-3-(3,5-dimethoxyphenyl)propanoic acid (6q)

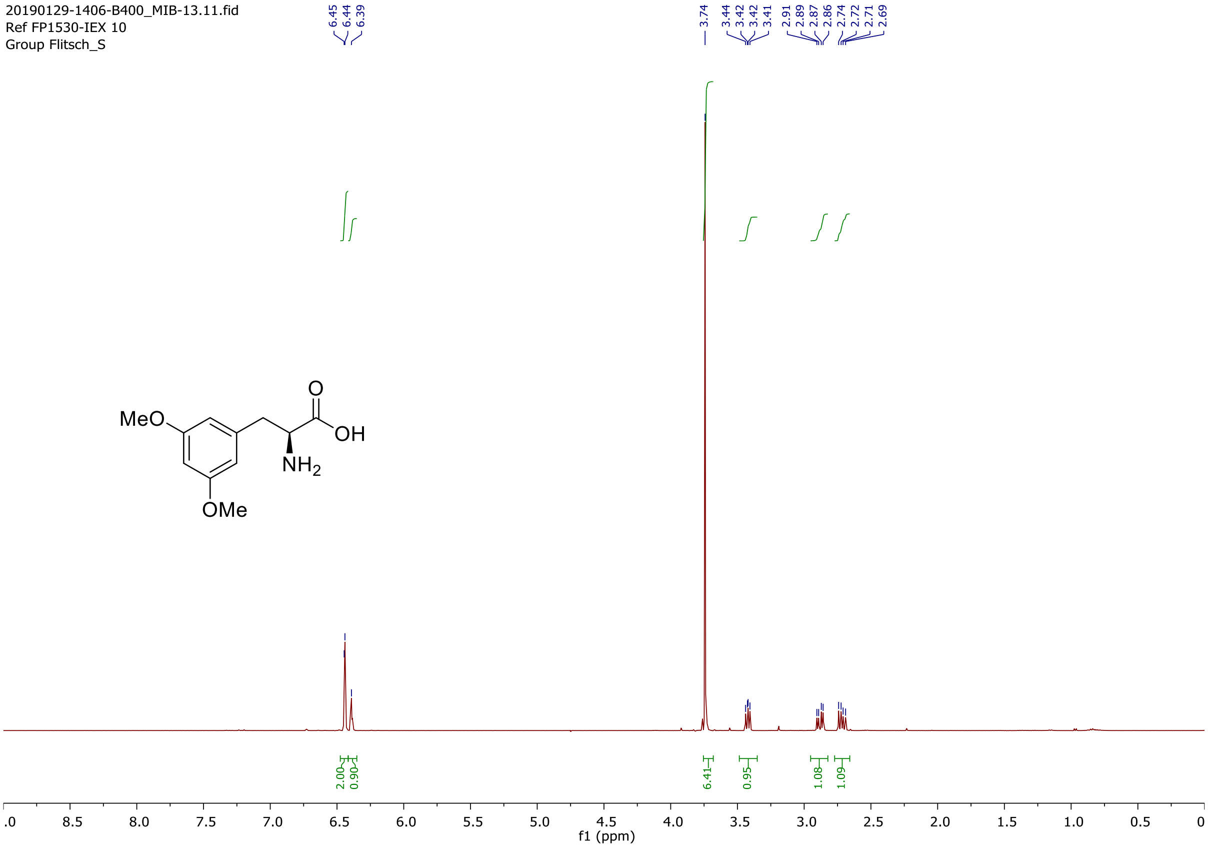

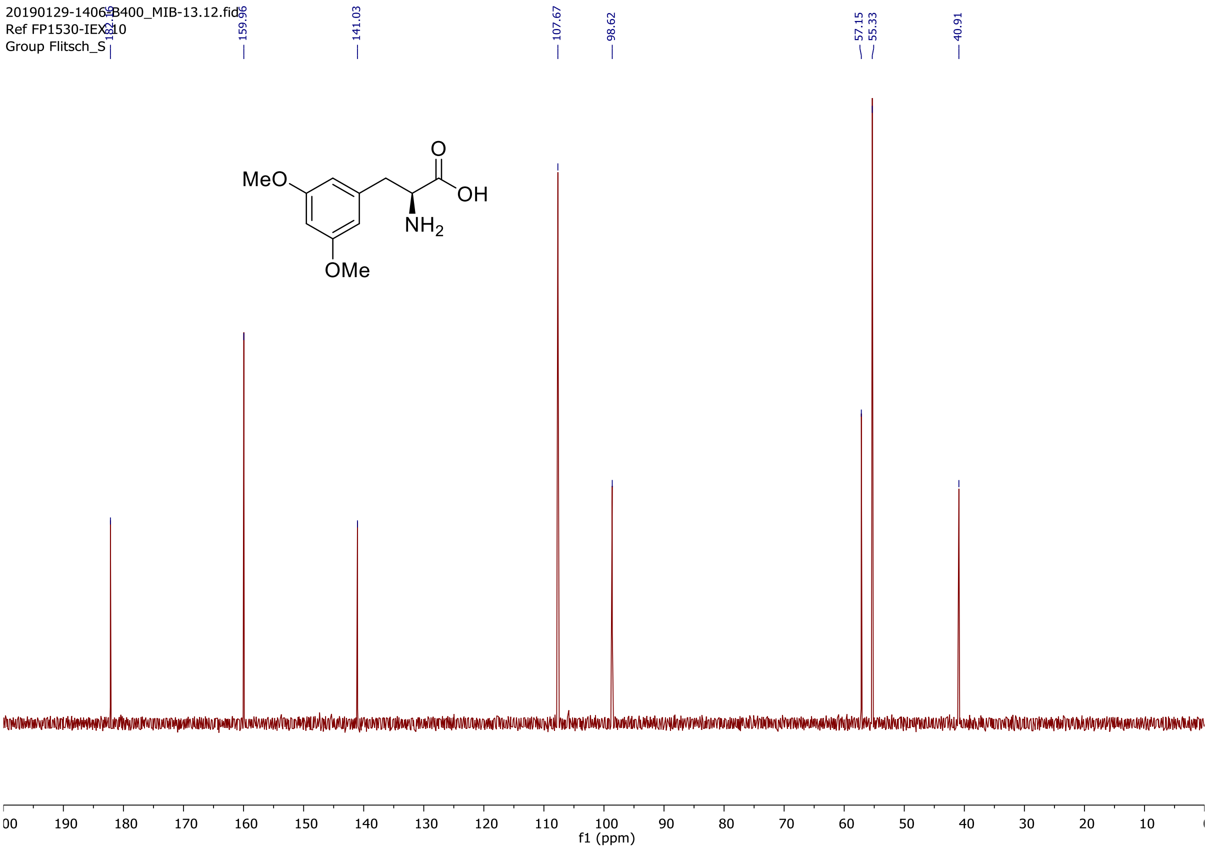

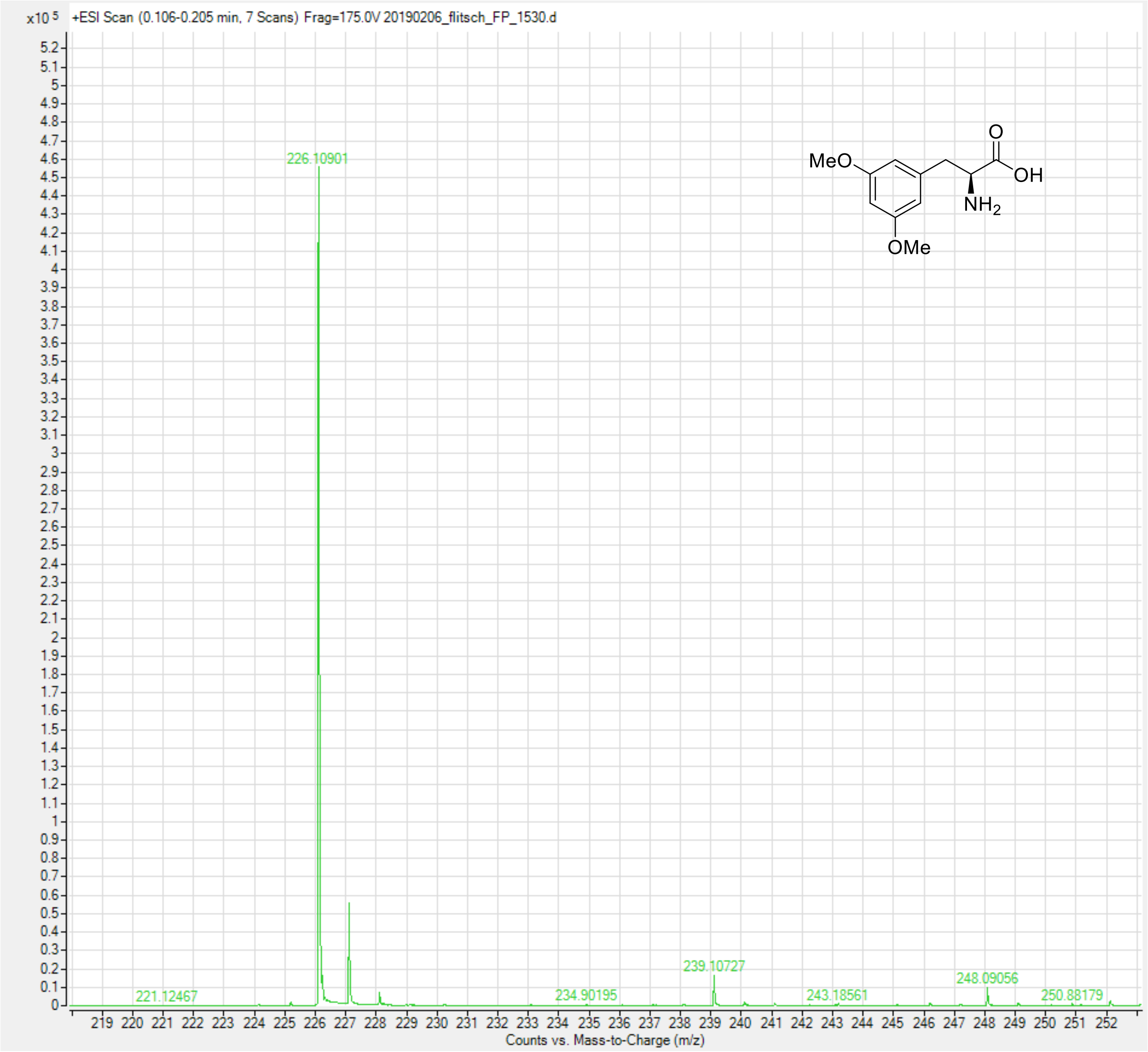
x10 5
+ESI Scan (0.106-0.205 min, 7 Scans) Frag=175.0V 20190206_flitsch_FP_1530.d
226.10901
239.10727
248.09056
221.12467
234.90195
243.18561
250.88179
Counts vs. Mass-to-Charge (m/z)
MeO
OMe
O
OH
NH2

(*S*)-2-amino-3-(3,4,5-trimethoxyphenyl)propanoic acid (6s)

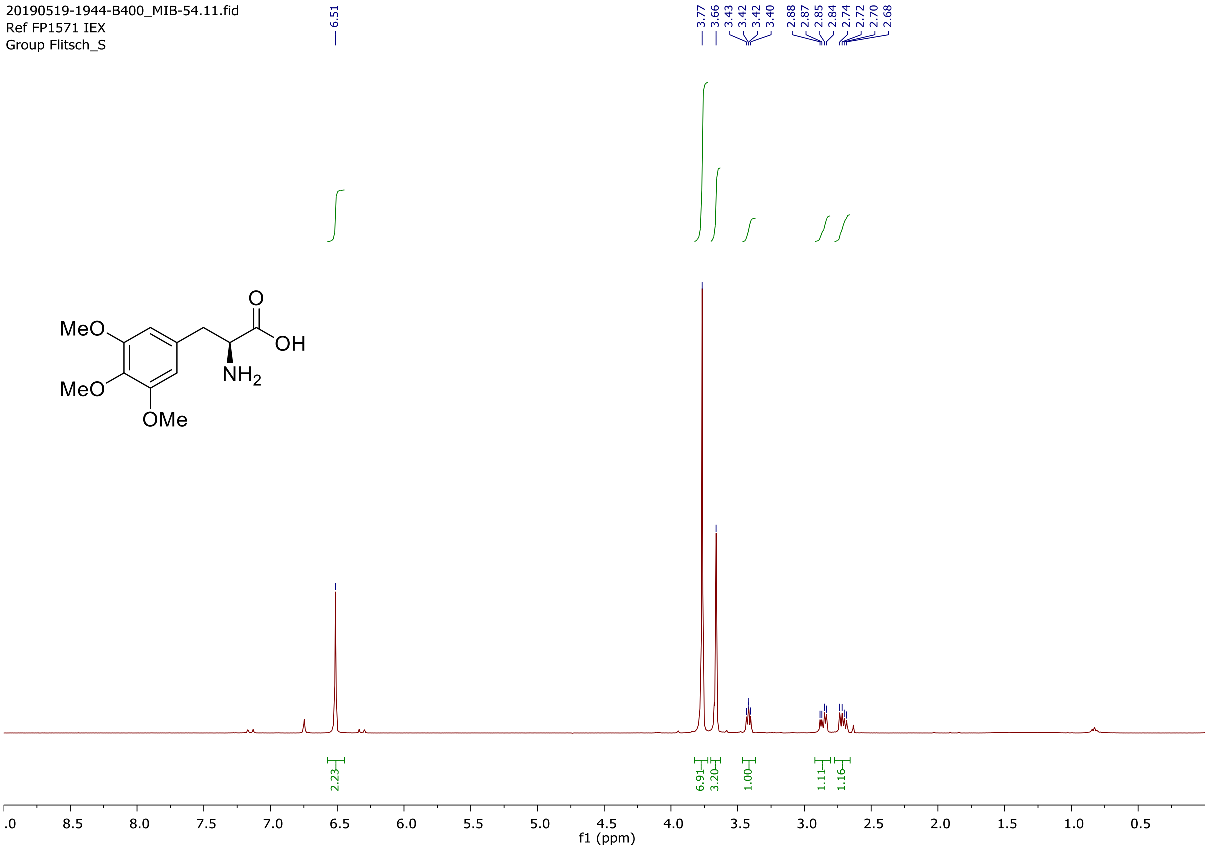

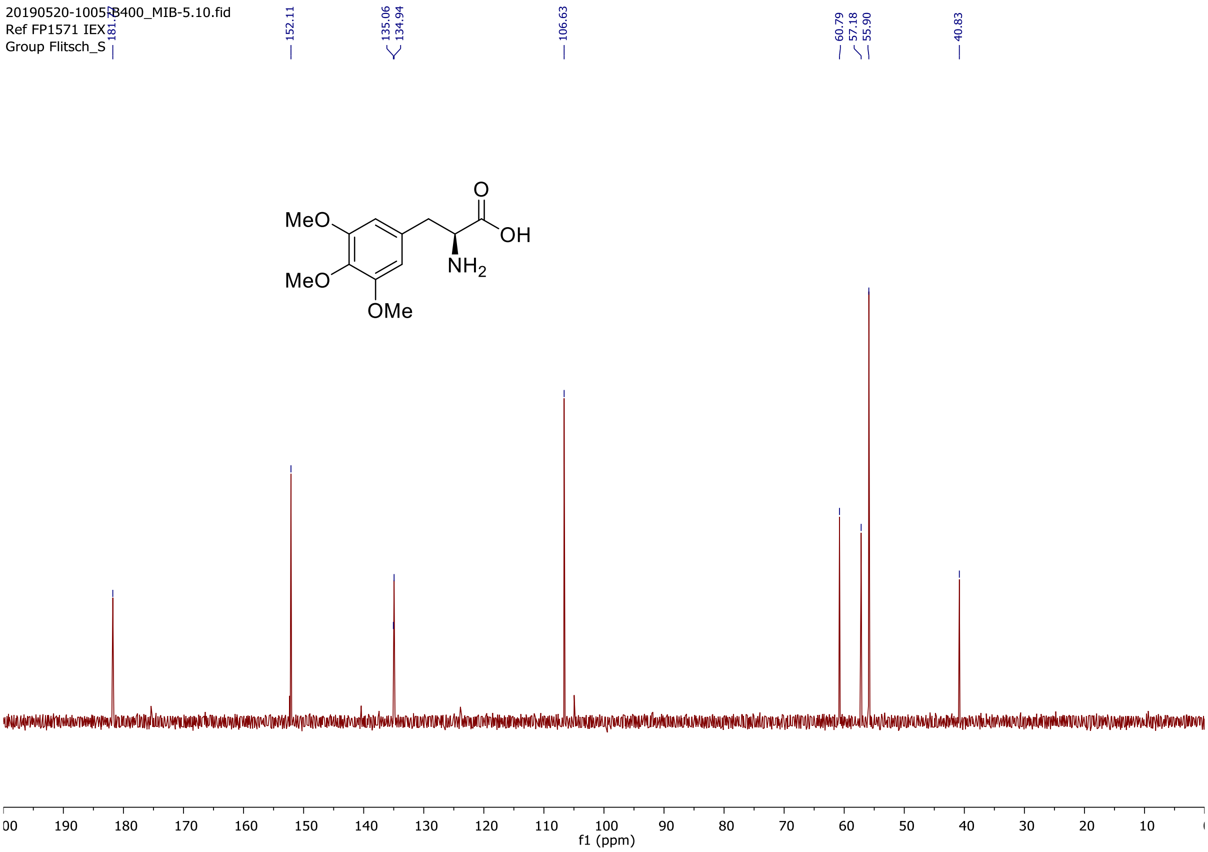

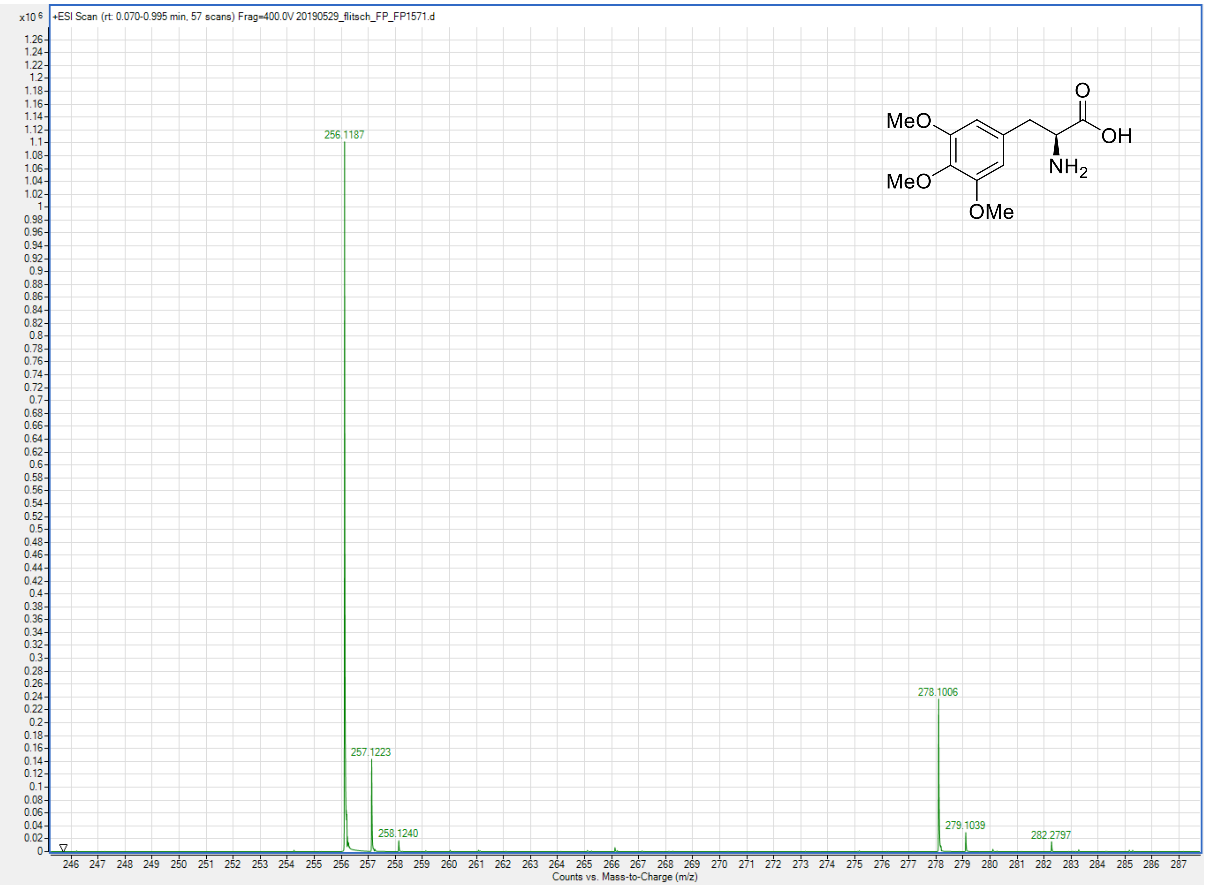

+ESI Scan (rt: 0.070-0.995 min, 57 scans) Frag=400.0V 20190529_flitsch_FP_FP1571.d
x10 6
256.1187
257.1223
258.1240
278.1006
279.1039
282.2797
Counts vs. Mass-to-Charge (m/z)
MeO
MeO
OMe
OH
O
NH2

(*S*)-2-amino-3-(2,3,4-trimethoxyphenyl)propanoic acid (6t)

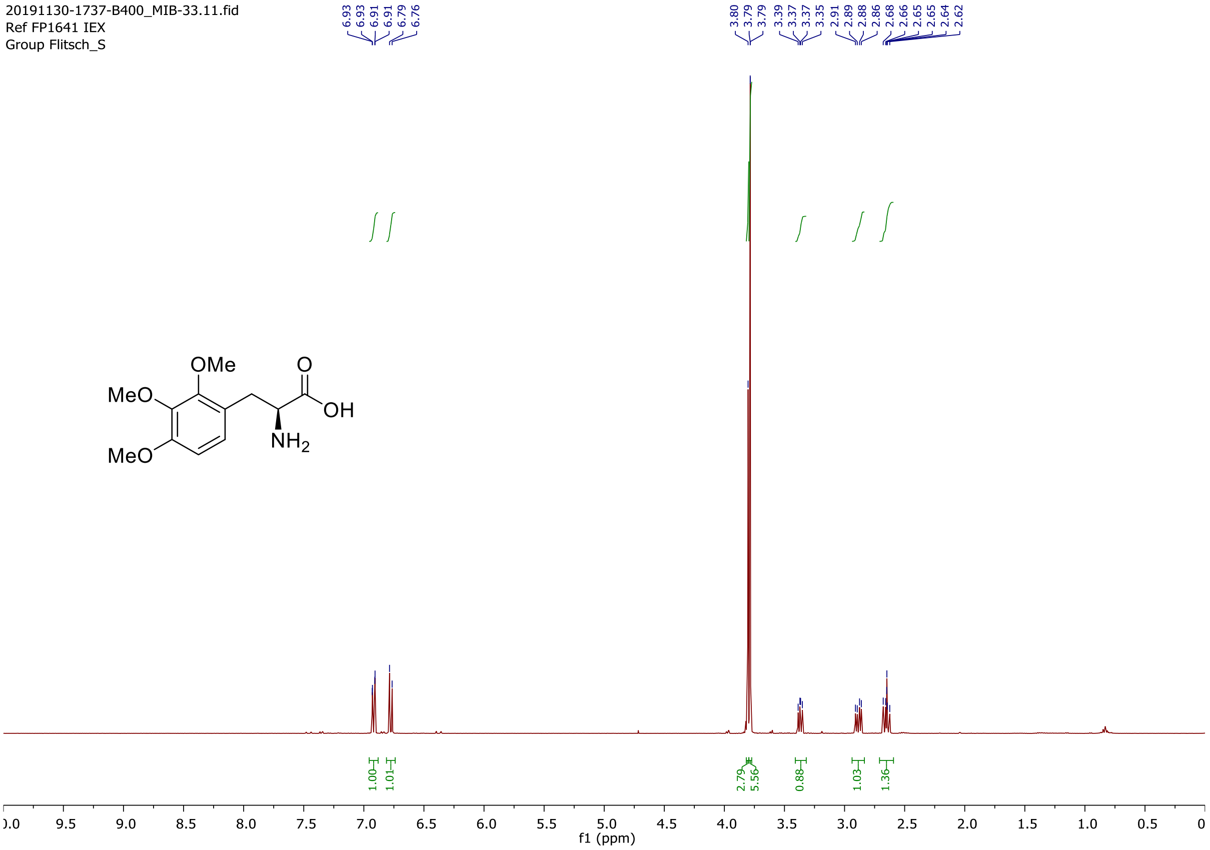

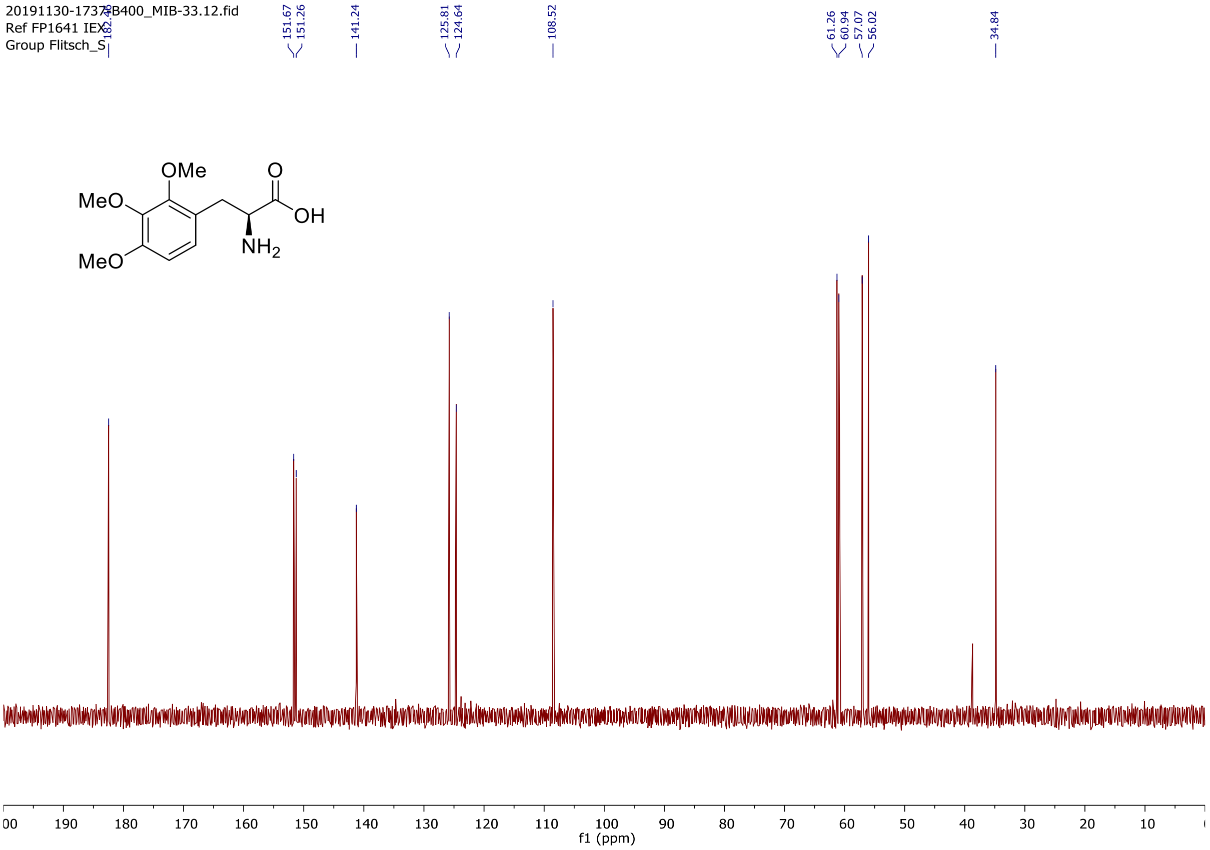

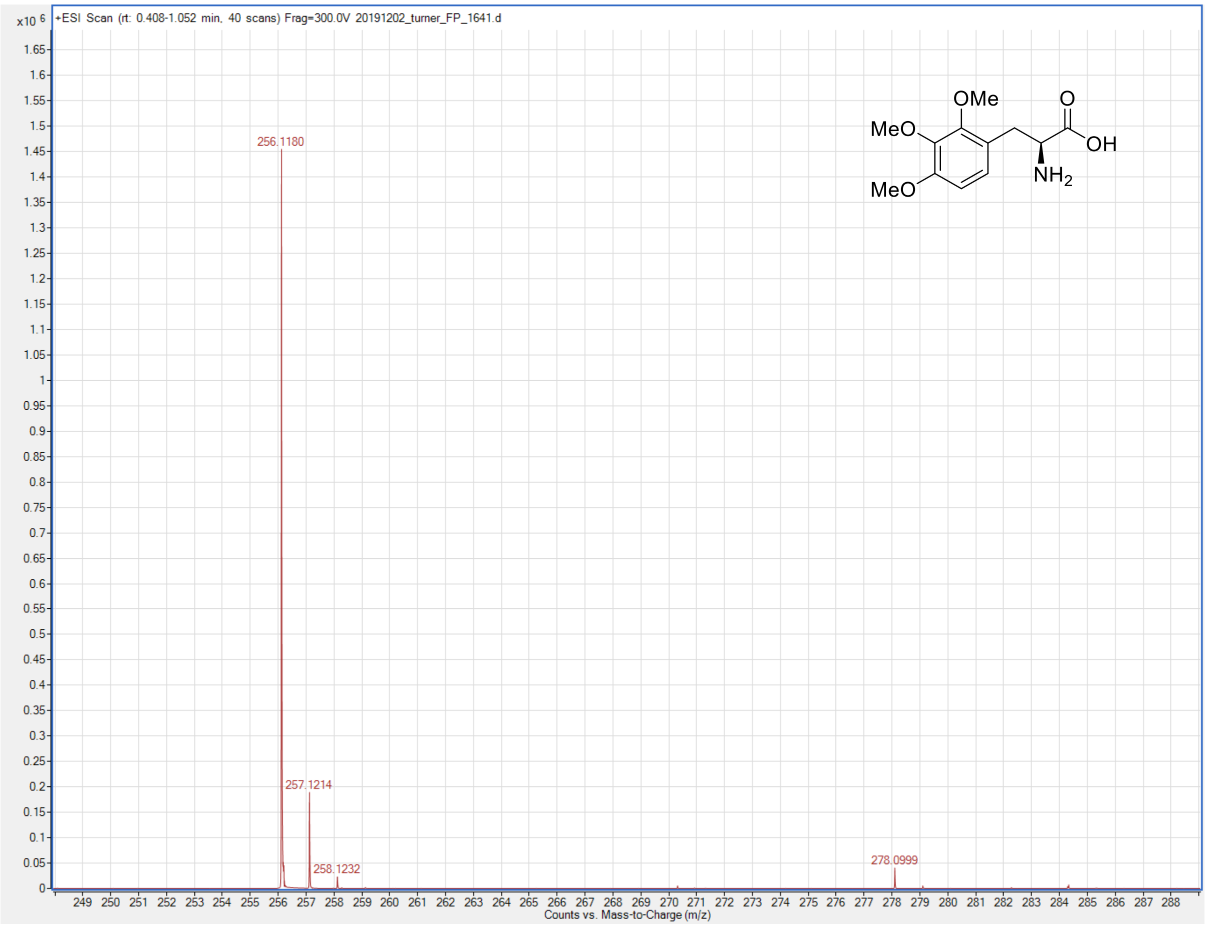

x10 6
+ESI Scan (rt: 0.408-1.052 min, 40 scans) Frag=300.0V 20191202_turner_FP_1641.d
256.1180
257.1214
258.1232
278.0999
OMe
MeO
MeO
O
OH
NH2
Counts vs. Mass-to-Charge (m/z)

(*S*)-2-amino-3-(2,4,5-trimethoxyphenyl)propanoic acid (6u)

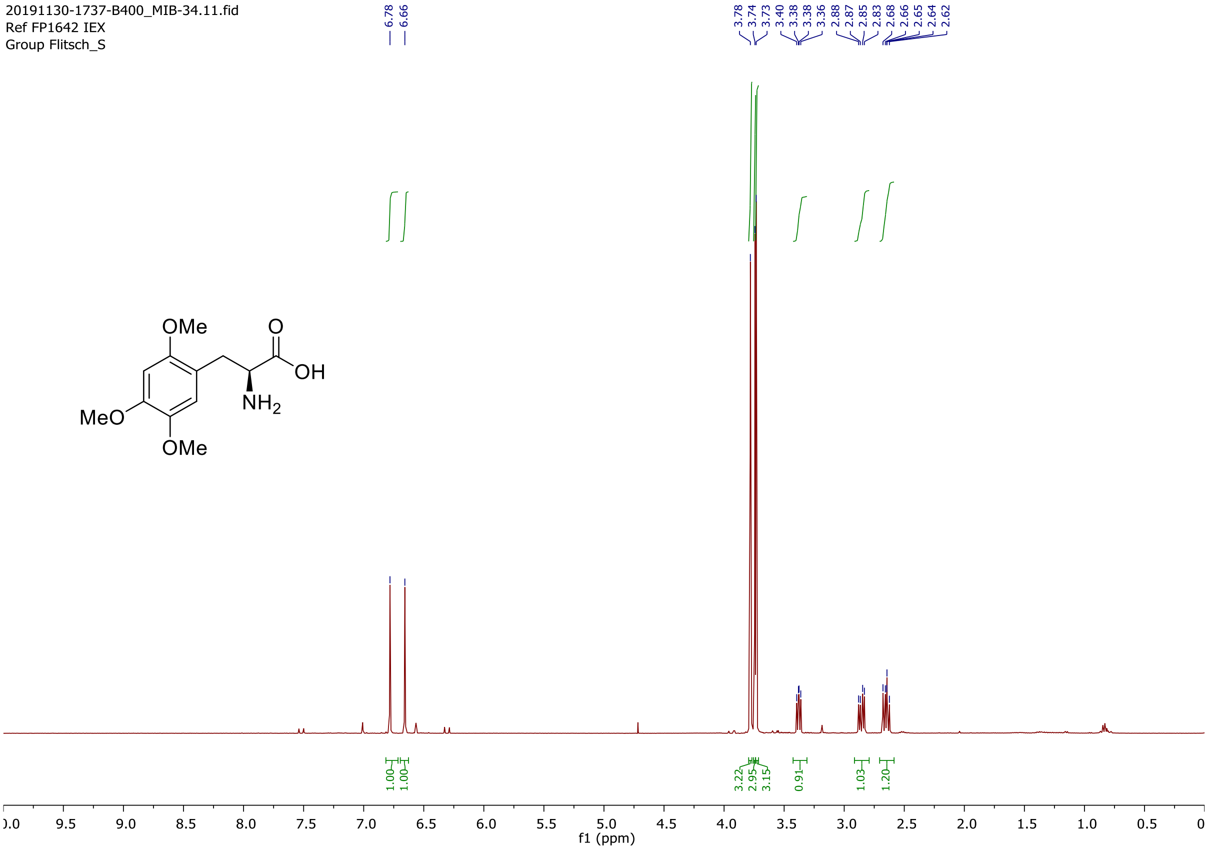

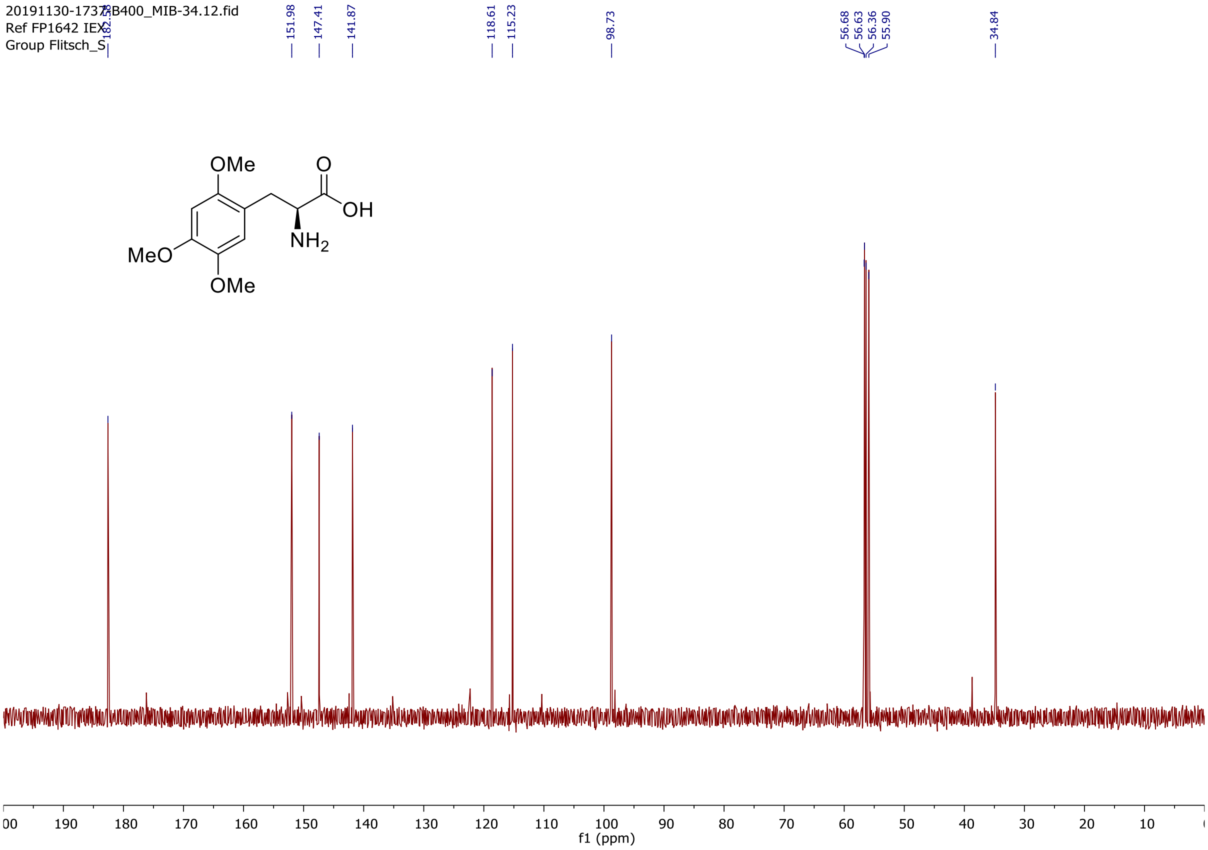

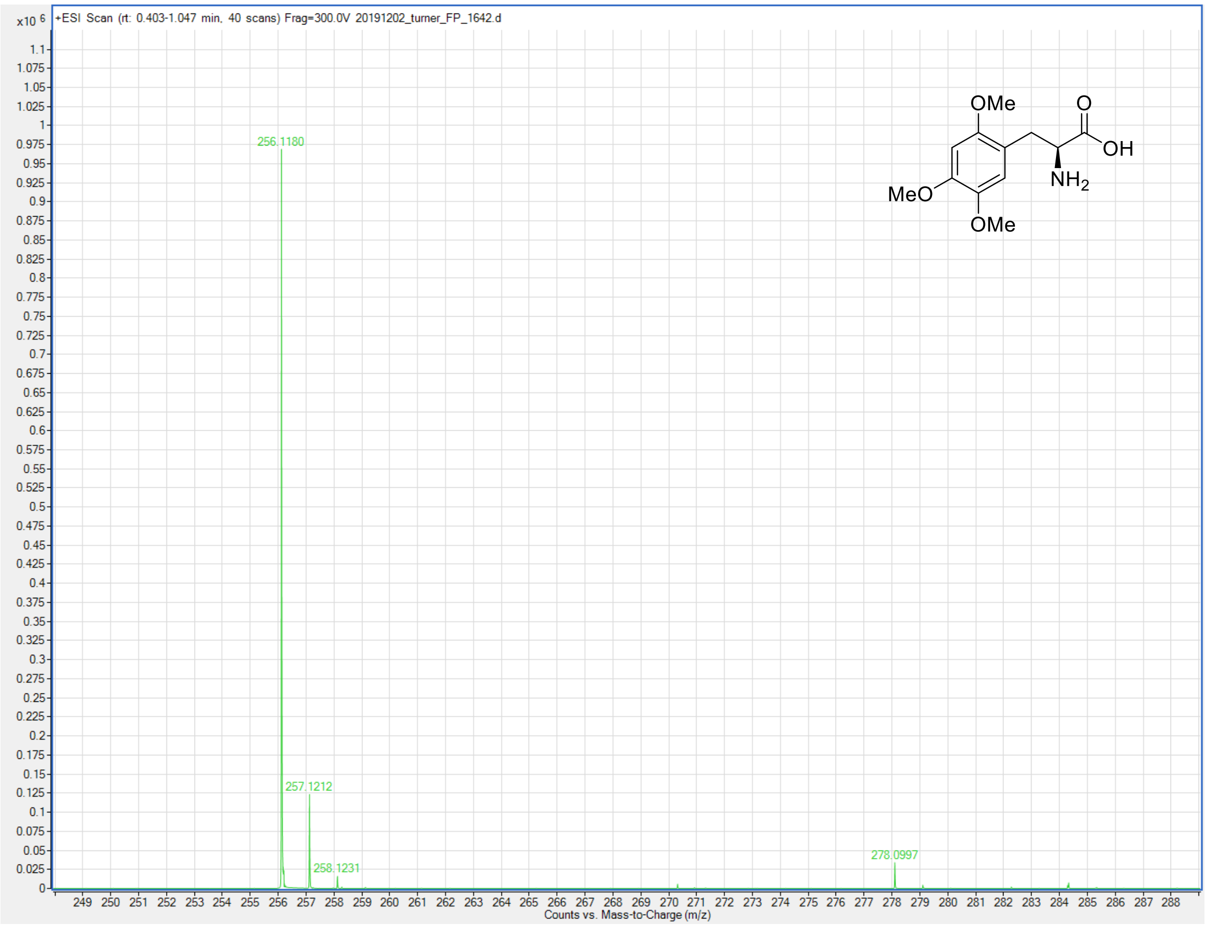

x10 6
+ESI Scan (rt: 0.403-1.047 min, 40 scans) Frag=300.0V 20191202_turner_FP_1642.d
256.1180
257.1212
258.1231
278.0997
OMe
O
OH
NH2
MeO
OMe
Counts vs. Mass-to-Charge (m/z)

HPLC traces for the isolated amino acids (*S*)-6

Determination of the *ee* values for isolated amino acids (*S*)-6

In the following figures, the upper trace shows the amino acid isolated from the PAL biotransformation, the lower trace shows a partially racemised sample to highlight the position of the D-enantiomer. The racemisation has been achieved by the addition of L-amino acid deaminase (LAAD) and a non-selective reducing agent (ammonia-borane complex), according to a previously reported method.[S1][S2]

Determination of the *ee* value of (*S*)-**6m**

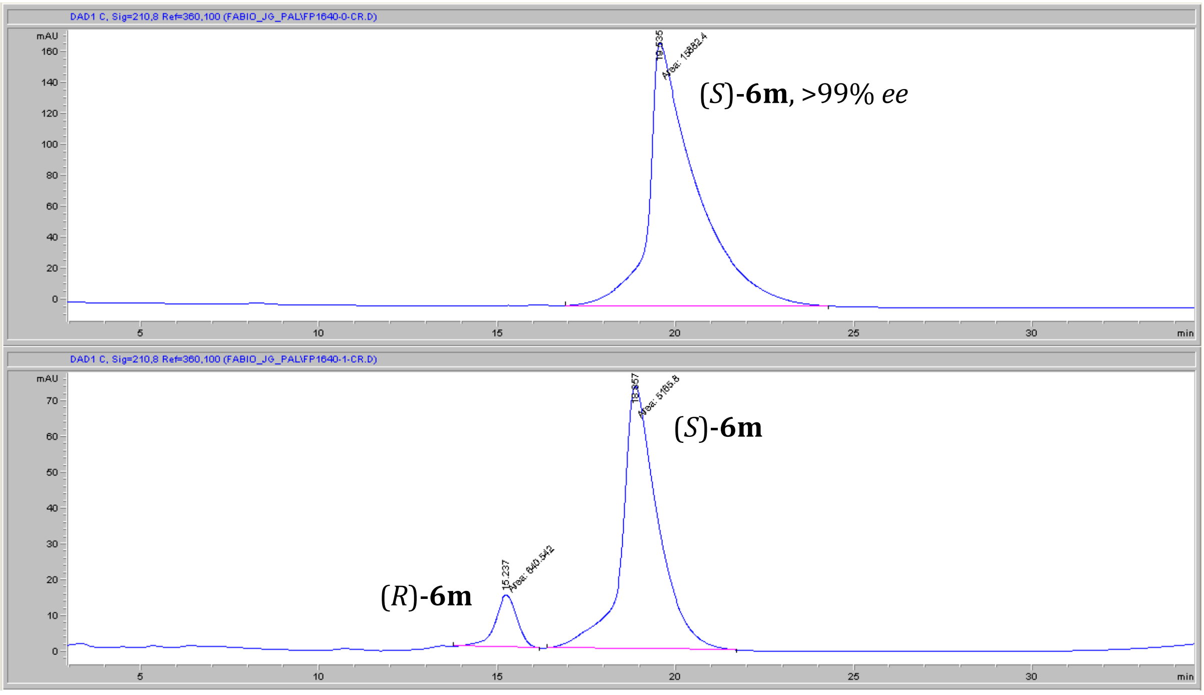

Determination of the *ee* value of (*S*)-**6o**

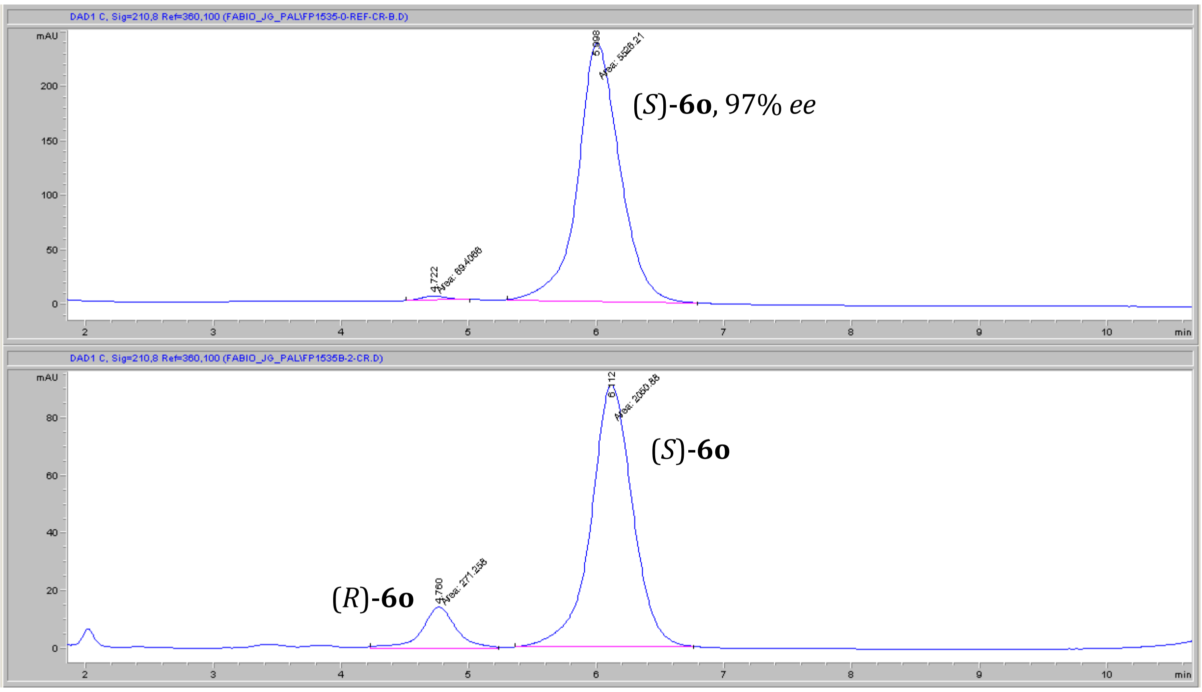

Determination of the *ee* value of (*S*)-**6p**

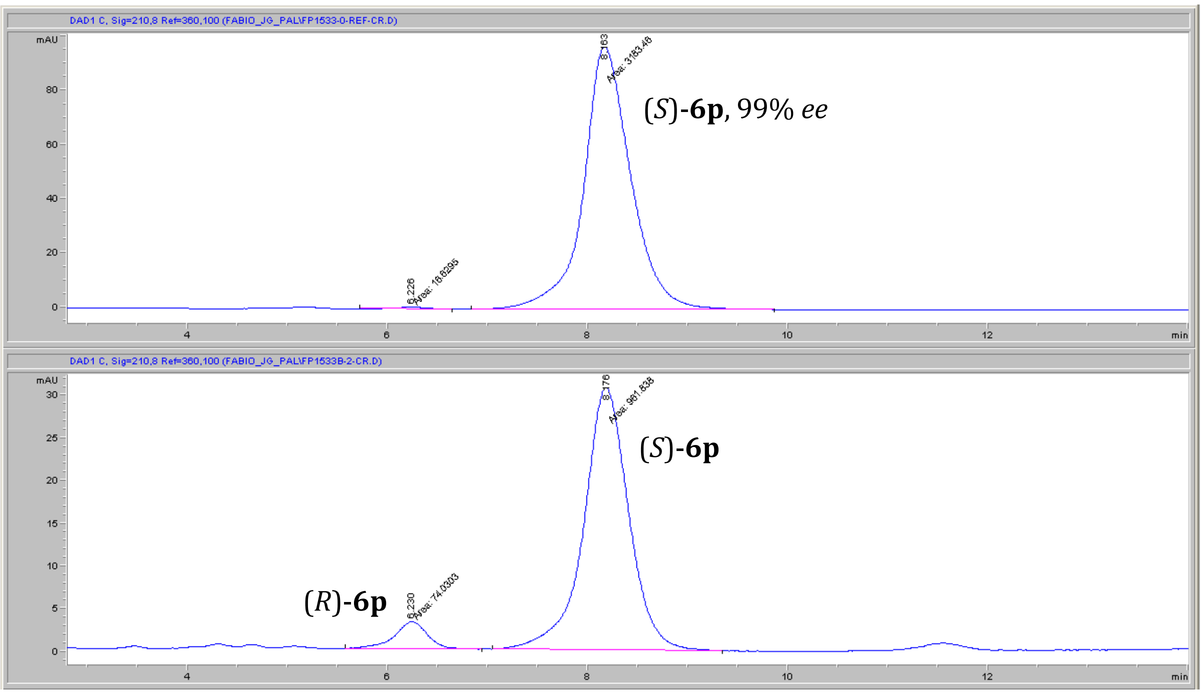

Determination of the *ee* value of (*S*)-**6q**

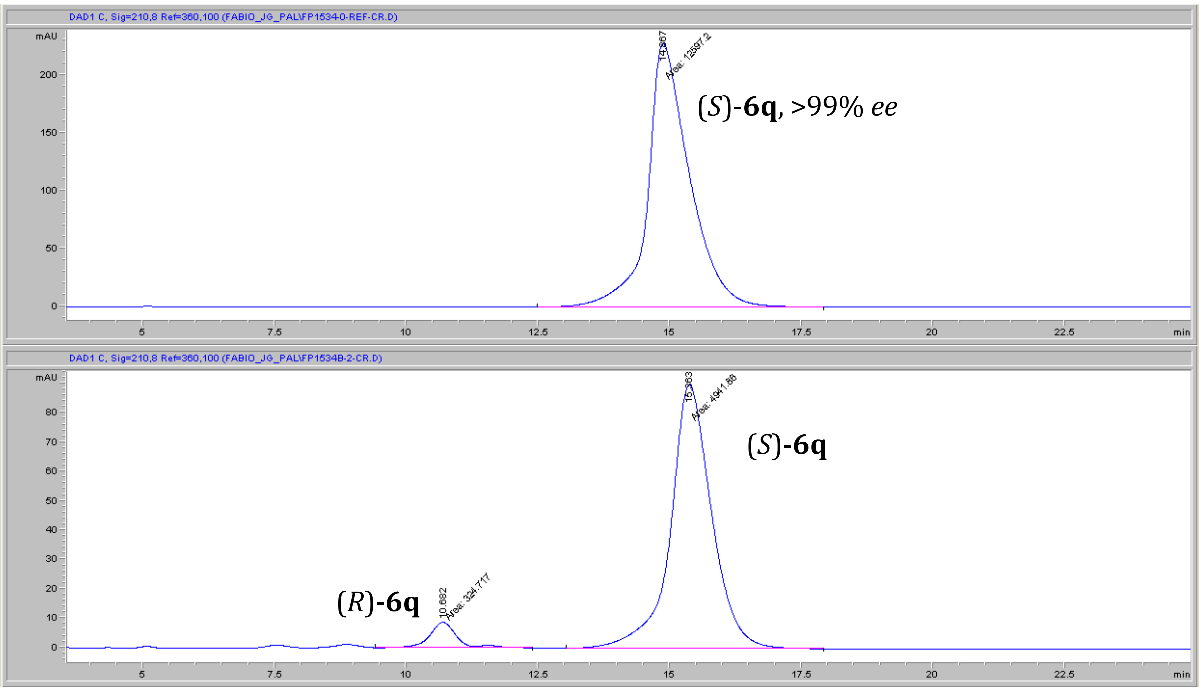

Determination of the *ee* value of (*S*)-**6s**

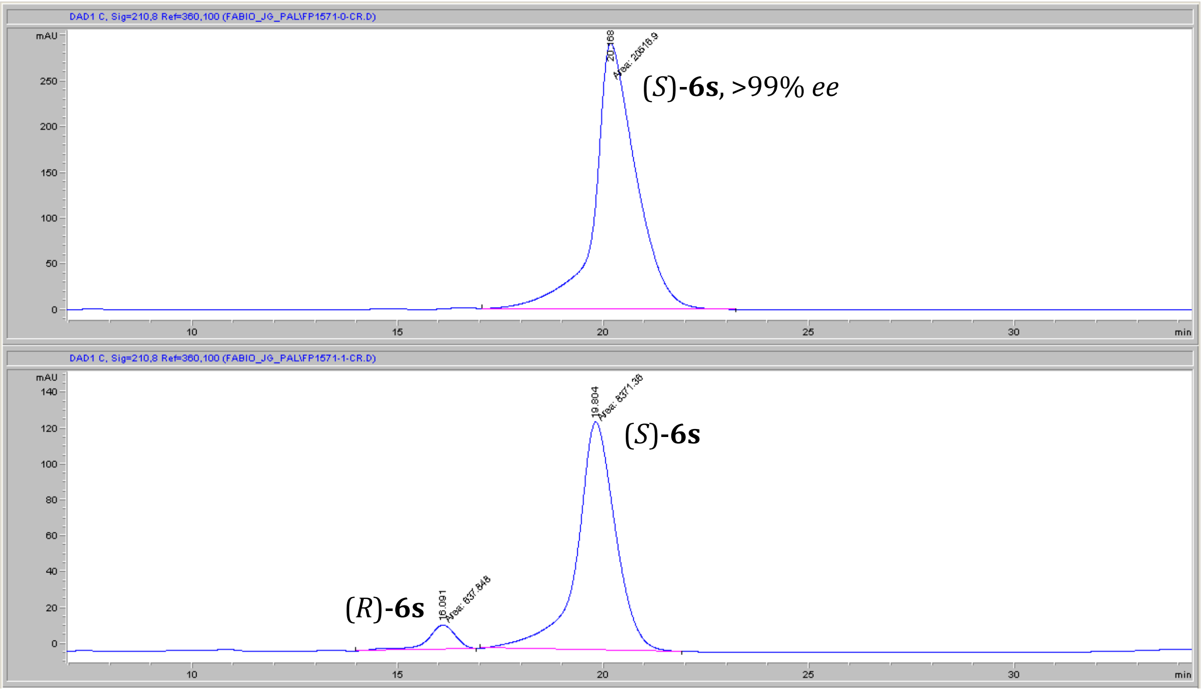

Determination of the *ee* value of (*S*)-**6t**

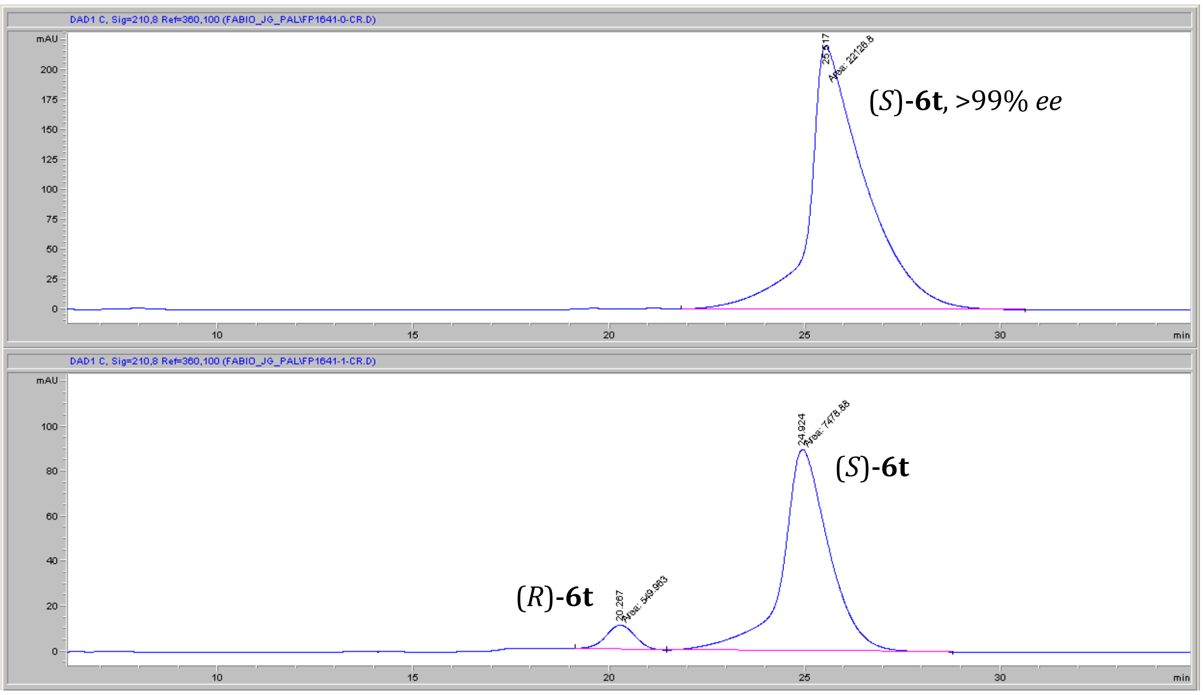

Determination of the *ee* value of (*S*)-**6u**

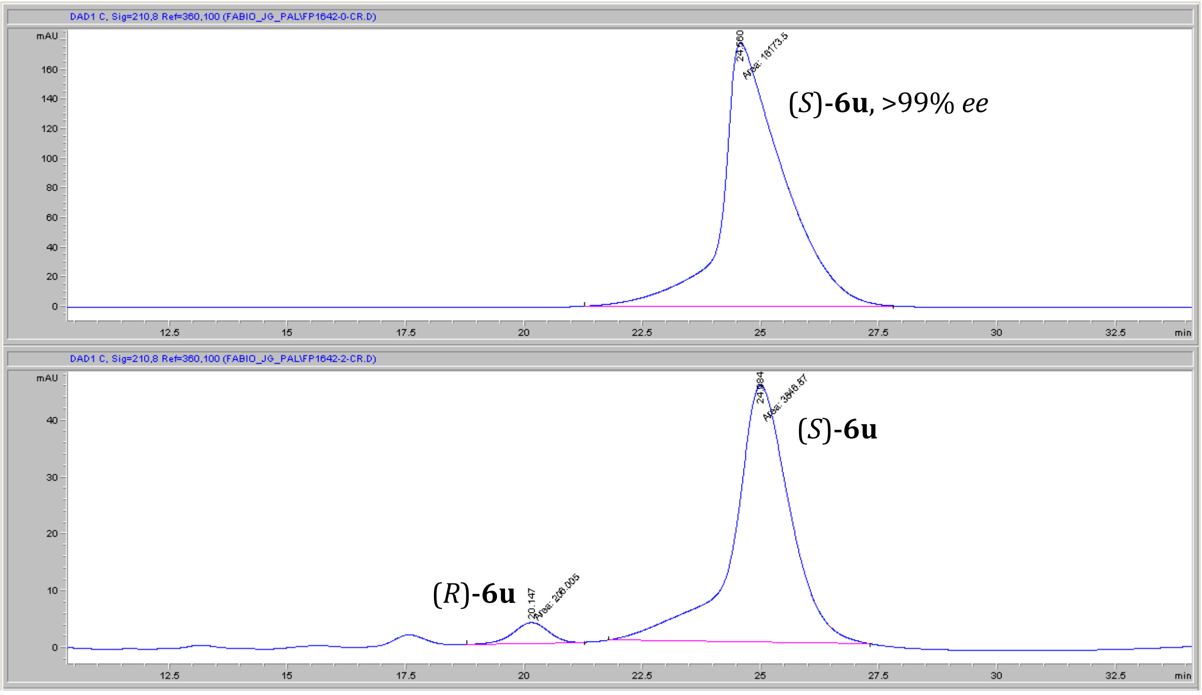

References

[S1] Parmeggiani F., Lovelock S. L., Weise N. J., Ahmed S. T., Turner N. J., Synthesis of D- and L-Phenylalanine Derivatives by Phenylalanine Ammonia Lyases: A Multienzymatic Cascade Process. *Angew. Chem. Int. Ed.* **2015**, *54*, 4608-4611.

[S2] Ahmed S. T., Parmeggiani F., Weise N. J., Flitsch S. L., Turner N. J., Synthesis of Enantiomerically Pure Ring-Substituted L-Pyridylalanines by Biocatalytic Hydroamination. *Org. Lett.* **2016**, *18*, 5468-5471.
